# Efficient Simulation of Higher-Order Transitions in Resonance Raman Spectroscopy and Its Chiral Extension

**DOI:** 10.1021/acs.jctc.6c01051

**Published:** 2026-08-29

**Authors:** Andrea Bianchi, Julien Bloino

**Affiliations:** 19004 Scuola Normale Superiore, Piazza dei Cavalieri 7, 56126 Pisa, Italy

## Abstract

We present an automated path-integral generator for resonance Raman (RR) calculations supporting Herzberg–Teller effects and Duschinsky rotation, and able to treat any type of vibrational transition, including overtones, combination bands, as well as hot bands. The method constructs the required tensor invariants directly from low-rank intermediates, avoiding both transition-specific manual derivations and the explicit construction of high-order correlation functions. The generated expressions are canonicalized symbolically and evaluated through optimized contraction paths. Compared to formulations based on explicit high-rank correlation tensors, this substantially lowers the asymptotic cost for several higher-order transition classes while providing a unified workflow for automatic expression generation. We validate the approach for multiple transition families, examine the influence of finite-temperature effects through hot-band contributions, and show how it can be straightforwardly extended to resonance Raman optical activity (RROA).

## Graphical abstract

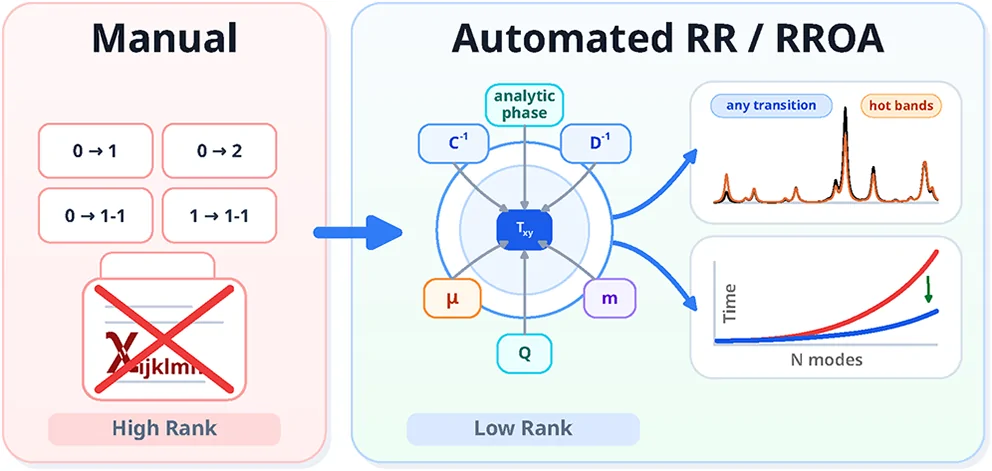

## 1 Introduction

In conditions of resonance, when the energy of the incident laser beam matches the electronic excitation energy of a molecule present in the sample, the Raman signal can be significantly, and selectively, enhanced, providing indirectly information on the electronic excited-state structure. Such characteristics are commonly used in the study of proteins or complex structures to probe specific centers, for instance metallic atoms, and the surrounding regions.^1,2^ The signal boost, which can reach up to a factor of 10^4^–10^6^,^3^ is critical for Raman optical activity (ROA) to compensate the weak chiroptical response.^4^ From a theoretical point of view, the resonance condition imposes a proper description of the intermediate state in the description of the polarizability tensor from the Kramers–Heisenberg–Dirac formulation of light scattering,^5^ and standard simplifications applied for conditions of incident light far from resonance, typically employed in electronic structure calculation packages, are not possible anymore. While pure electronic structure-based variants have been proposed to predict the molecular response close to resonance and avoid any singularity,^3,6,7^ a more accurate picture is achieved by properly accounting for the vibrational structure in electronic states within the so-called vibrationally resolved electronic formalism, often referred to as vibronic formalism.

The theoretical description of resonance Raman (RR) spectroscopy traces back to the vibronic framework of Albrecht.^8^ Modern computational approaches to RR and RROA generally fall into two categories: time-independent (TI) sum-over-states methods and time-dependent (TD) or path-integral formalisms.^9–14^ TI methods are conceptually straightforward, but their cost rises steeply with the size of the system because they typically require summations over a large number of intermediate vibronic states. Because the number of states to include is in theory infinite, reliable truncation schemes are necessary. While techniques exist for one-photon absorption (OPA), they are not readily applicable to Raman, owing to the fact that two transition integrals are involved (initial → intermediate and intermediate → final). Moreover, the form of the polarizability tensor does not allow building a metric to assess the impact of the truncation, as is commonly done in OPA with the use of analytic sum rules.^12,15^ The situation is even more intricate with RROA, which combines three properties, the electric dipole-electric dipole tensor (polarizability), the electric dipole-magnetic dipole tensor and electric dipole-electric quadrupole tensor.^16,17^ Path-integral methods avoid this combinatorial growth and the truncation issue altogether, by expressing the scattering cross-section as the Laplace transform of a time-correlation function. For harmonic potential energy surfaces, the required correlation functions can be obtained analytically.^18–20^

Recent works have woven a broad path-integral framework for simulating these spectra. Some of us developed a time-dependent approach for RR^13^ and later extended it to RROA,^16^ incorporating both Herzberg–Teller (HT) effects and mode mixing through the Duschinsky transformation.^21^ Closely related analytic time-dependent approaches were also developed by Petrenko, Neese, and co-workers.^22,23^ These methods were formulated primarily for fundamental transitions from the vibrational ground state. More recently, de Souza et al.^14^ extended the path-integral RR formalism to overtones and “1 + 1” binary combination bands. However, for any other transition, complete derivations are needed, and the explicit evaluation of transition-specific high-order correlation functions remains algebraically cumbersome and computationally demanding. In the formulation of de Souza et al., the corresponding contractions scale formally as $O\left(N^{6}\right)$ with the number of modes *N*, so numerical screening thresholds were introduced to discard negligible terms and make calculations feasible. A remaining challenge is treating complex, higher-order vibrational transitions while avoiding redundant transition-specific derivations, especially for finite-temperature simulations that require averaging over multiple thermally populated initial states.

Here we introduce an automated symbolic framework to generate path-integral RR tensor invariants on-the-fly, with a formulation that transfers directly to RROA properties. The method builds the required expressions from low-rank objects, canonicalizes equivalent contraction graphs symbolically, and evaluates the result through optimized contraction paths with intermediate reuse, avoiding manually derived correlation functions and explicit construction of high-rank intermediates. The principal gains are the elimination of transition-specific derivations and substantial reductions in computational cost for several transition classes, improving the asymptotic scaling of the contractions relative to explicit high-rank tensor expressions. We validate the method across several types of transitions, use it to examine finite-temperature effects through low-lying hot bands, and demonstrate its applicability to RROA on (*S*)-(+)-naproxen with two closely lying intermediate electronic states.

## 2 Theory

RR and RROA observables are determined by transition response tensors connecting an initial molecular state |Ψ_*i*_⟩ to a final molecular state |Ψ_*f*_⟩. For RR, the relevant quantity is the electric dipole-electric dipole polarizability tensor **α**_*if*_, and the observable intensity is obtained from the standard scalar invariants^5^

1 $$I_{i\to f}=f\left(a_{\mathrm{if}}^{2},g_{\mathrm{if}}^{2},d_{\mathrm{if}}^{2}\right)$$

2 $$a_{\mathrm{if}}^{2}=\frac{1}{9}{\left|\sum_{\rho }\alpha_{\mathrm{if},\rho \rho }\right|}^{2}$$

3 $$g_{\mathrm{if}}^{2}=\frac{1}{2}\left(3\sum_{\rho \sigma }|\alpha_{\mathrm{if},\rho \sigma }{|}^{2}-{\left|\sum_{\rho }\alpha_{\mathrm{if},\rho \rho }\right|}^{2}\right)$$

4 $$d_{\mathrm{if}}^{2}=\frac{3}{4}\left(\sum_{\rho \sigma }|\alpha_{\mathrm{if},\rho \sigma }{|}^{2}-\sum_{\rho \sigma }\alpha_{\mathrm{if},\rho \sigma }\alpha_{\mathrm{if},\sigma \rho }\right)$$

where the expression for *f*(*a*_*if*_^2^, *g*_*if*_^2^, *d*_*if*_^2^) depends on the experimental setup.^5^ For RROA, the differential scattering cross sections involve other properties, namely the electric dipole-magnetic dipole and electric dipole-electric quadrupole tensors, usually denoted ***G*** and ***A***, which enter into the observable through analogous scalar invariants built from contractions with **α**.^16,24^ Below, we derive the formalism for a generic tensor, denoted ***T***; the same construction applies to both RR and RROA tensors.

In the TI approach, a generic transition tensor element *T*_*if*,ρ*η*_ can be written as

5 $$T_{\mathrm{if},\rho \eta }=\frac{1}{\hbar }\sum_{m\neq i,f}\left(\frac{\left\langle \Psi_{f}\right|P_{\rho }^{A}\left|\Psi_{m}\right\rangle \left\langle \Psi_{m}\right|P_{\eta }^{B}\left|\Psi_{i}\right\rangle }{\omega_{\mathrm{mi}}-\omega_{I}-i\gamma_{m}}+\frac{\left\langle \Psi_{f}\right|P_{\eta }^{B}\left|\Psi_{m}\right\rangle \left\langle \Psi_{m}\right|P_{\rho }^{A}\left|\Psi_{i}\right\rangle }{\omega_{\mathrm{mi}}+\omega_{I}+i\gamma_{m}}\right)$$

where *P*_ρ_^*A*^ and *P*_η_^*B*^ are the relevant transition operators, the indices ρ and η go from *x* to *z* for the electric and magnetic dipole operators, or from *xx* to *zz* for the quadrupole operator; ω_*I*_ is the incident frequency, and ω_*mi*_ = ω_*m*_ – ω_*i*_ is the frequency difference between the intermediate (*m*) and initial states. For RR, ***P*** ^*A*^ = ***P*** ^*B*^ = **μ**, whereas for RROA they correspond to the appropriate combinations of electric dipole, magnetic dipole, and electric quadrupole operators. In the resonance regime, the first term dominates and can be recast within a time-dependent path-integral framework.

### 2.1 Path-Integral Formulation

Besides the Born–Oppenheimer approximation, we assume that the Eckart conditions are met, so the coupling between vibrations and rotations is minimized and the latter will be neglected in the following. In this setup, the integrals in eq 5 occur between vibrational states of distinct electronic states. Furthermore, we adopt the harmonic approximation for both the initial and resonant electronic states. In the resonant, two-state picture considered here, also known as single electronic state formalism, we treat one intermediate electronic state explicitly. Extension to multiple intermediate states is straightforward: the total tensor in eq 5 is obtained as the sum of the individual electronic state-specific contributions.

Let ***Q̅*** and $\overset{®}{\mathrm{Q̅}}$ be the mass-weighted normal coordinates of the initial and resonant electronic states, respectively. Their vibrational Hamiltonians are

6 $$\mathrm{H̅}=-\frac{\hbar^{2}}{2}{\left(\frac{\partial }{\partial \mathrm{Q̅}}\right)}^{T}\left(\frac{\partial }{\partial \mathrm{Q̅}}\right)+\frac{1}{2}{\mathrm{Q̅}}^{T}{\mathrm{\Omega ̅}}^{2}\mathrm{Q̅}$$

7 $$\overset{®}{\mathrm{H̅}}=-\frac{\hbar^{2}}{2}{\left(\frac{\partial }{\partial \overset{®}{\mathrm{Q̅}}}\right)}^{T}\left(\frac{\partial }{\partial \overset{®}{\mathrm{Q̅}}}\right)+\frac{1}{2}{\overset{®}{\mathrm{Q̅}}}^{T}{\overset{®}{\mathrm{\Omega ̅}}}^{2}\overset{®}{\mathrm{Q̅}}+E_{\mathrm{ad}}$$

where **Ω̅** and $\overset{®}{\mathrm{\Omega ̅}}$ are diagonal matrices of harmonic frequencies and *E*_ad_ is the adiabatic electronic excitation energy.

For a transition from an initial vibrational state |ψ_*i*_⟩ to a final vibrational state |ψ_*f*_⟩, the resonant contribution to the tensor element is written as a Laplace transform of a time-correlation function

8 $$T_{\mathrm{if},\rho \eta }=\frac{i}{\hbar^{2}}\int_{0}^{+\infty }dt\left\langle {\mathrm{\psi ̅}}_{f}\right|P_{\rho }^{A,e}e^{-i/\hbar \overset{®}{\mathrm{H̅}}t}P_{\eta }^{B,e}\left|{\mathrm{\psi ̅}}_{i}\right\rangle e^{-\mathrm{it}\left(\omega_{\mathrm{ad}}-\omega_{I}-i\gamma \right)}$$

Here, ω_ad_ is the transition frequency associated with *E*_ad_, *P*_ρ_^*A,e*^ = ⟨ϕ_*i*_|*P*_ρ_^*A*^|ϕ_*m*_⟩ and *P*_η_^*B,e*^ = ⟨ϕ_*m*_|*P*_η_^*B*^|ϕ_*f*_⟩ are the electronic matrix elements of the transition operators. Since |ϕ_*i*_⟩ = |ϕ_*f*_⟩, the transition moments refer to the same electronic states, which were omitted in the shortened notation for convenience. The damping factor γ_*m*_ is considered constant for all the vibrational states of a given electronic state,^25,26^ and so, in the two-states picture considered here, the subscript has been dropped.

In the absence of an analytic form of the dependence of ***P*** ^*X*,*e*^ with respect to the nuclear coordinates, the transition operators are usually expanded as Taylor series about one of the equilibrium geometries

9 $$P^{X,e}\left(Q\right)=P^{X,e}\left(Q^{\mathrm{eq}}\right)+\nabla P^{X,e}\left(Q^{\mathrm{eq}}\right)\cdot Q+\cdot \cdot \cdot$$

The zeroth-order term gives the Franck–Condon (FC) contribution, whereas the term linear in the normal coordinates corresponds to the Herzberg–Teller (HT) contribution. In this work, the acronym FCHT will be used to refer to an expansion up to the first order. In standard approaches where the potential energy surfaces (PESs) of the initial (typically ground) and intermediate states are computed at their respective minimum, the excited-state normal modes are used as reference since the expansion terms are readily accessible from electronic structure calculations. For so-called vertical methods, where the intermediate-state PES is expanded about the ground-state equilibrium geometry, the expansion is carried out with respect to the initial state. To facilitate implementations, a simple strategy is to build the formalism for a given set of coordinates and convert from the other if necessary, assuming that the expansion up to the first order is exact.

Applying the expansion from eq 9 on ***P*** ^*A*,*e*^ and ***P*** ^*B*,*e*^ and inserting them into eq 8 generates a hierarchy of correlation functions associated with the FC, mixed FC/HT, and pure HT terms. We focus on the contribution obtained from the product of the linear terms in the two expansions, denoted as

10 $$\chi_{\mathrm{if}}^{11}\left(t\right)=\left\langle {\mathrm{\psi ̅}}_{f}\right|\overset{®}{\mathrm{Q̅}}e^{-i/\hbar \overset{®}{\mathrm{H̅}}t}\overset{®}{\mathrm{Q̅}}\left|{\mathrm{\psi ̅}}_{i}\right\rangle$$

which identifies the pure HT contribution. The other terms can be treated in the same way, but involve lower-degree polynomials, so they will not be discussed in the following.

The reduction of the representative Herzberg–Teller term χ_*if*_^11^(*t*) to Gaussian moments proceeds by inserting coordinate-space resolutions of the identity for both the initial-state and resonant-state normal coordinates

11 $$\chi_{\mathrm{if}}^{11}\left(t\right)=\int d\overset{®}{\mathrm{Q̅}}d{\overset{®}{\mathrm{Q̅}}}^{\prime }d\mathrm{Q̅d}{\mathrm{Q̅}}^{\prime }\left\langle {\mathrm{\psi ̅}}_{f}\right|{\mathrm{Q̅}}^{\prime }\rangle \left\langle {\mathrm{Q̅}}^{\prime }\right|{\overset{®}{\mathrm{Q̅}}}^{\prime }\rangle \left\langle {\overset{®}{\mathrm{Q̅}}}^{\prime }\right|\overset{®}{\mathrm{Q̅}}e^{-\left(i/\hbar \right)\overset{®}{\mathrm{H̅}}t}\overset{®}{\mathrm{Q̅}}\left|\overset{®}{\mathrm{Q̅}}\right\rangle \left\langle \overset{®}{\mathrm{Q̅}}\right|\mathrm{Q̅}\rangle \left\langle \mathrm{Q̅}\right|{\mathrm{\psi ̅}}_{i}\rangle$$

The resonant-state propagator is evaluated analytically through the multidimensional Mehler kernel,^27,28^

$$\left\langle {\overset{®}{\mathrm{Q̅}}}^{\prime }\right|\overset{®}{\mathrm{Q̅}}e^{-i/\hbar \overset{®}{\mathrm{H̅}}t}\overset{®}{\mathrm{Q̅}}\left|\overset{®}{\mathrm{Q̅}}\right\rangle =\sqrt{\frac{\det\overset{®}{\mathrm{a̅}}}{{\left(2\pi \right)}^{N}}}{\overset{®}{\mathrm{Q̅}}}^{\prime }\exp\left[i\left(\frac{1}{2}{\overset{®}{\mathrm{Q̅}}}^{\prime T}\overset{®}{\mathrm{b̅}}{\overset{®}{\mathrm{Q̅}}}^{\prime }+\frac{1}{2}{\overset{®}{\mathrm{Q̅}}}^{T}\overset{®}{\mathrm{b̅}}\overset{®}{\mathrm{Q̅}}-{\overset{®}{\mathrm{Q̅}}}^{T}\overset{®}{\mathrm{a̅}}{\overset{®}{\mathrm{Q̅}}}^{\prime }\right)\right]\overset{®}{\mathrm{Q̅}}$$ 12

with

13 $$\overset{®}{\mathrm{a̅}}=\overset{®}{\mathrm{\Omega ̅}}\mathrm{csch}\left(i\overset{®}{\mathrm{\Omega ̅}}t\right)$$

14 $$\overset{®}{\mathrm{b̅}}=\overset{®}{\mathrm{\Omega ̅}}\coth\left(i\overset{®}{\mathrm{\Omega ̅}}t\right)$$

The overlap between the two sets of normal coordinates is represented through the Duschinsky transformation^21^

15 $$\mathrm{Q̅}=J\overset{®}{\mathrm{Q̅}}+K$$

where ***J*** is the Duschinsky matrix, associated with the mode mixing, and ***K*** is the shift vector.

No hypothesis is made on the form of ***J***, be it its orthogonality or sparsity. Indeed, while the matrix is orthogonal for Cartesian-based normal coordinates considered in this work, this is not true for internal coordinates.^29^ The method proposed here can thus be applied directly in any condition where the Duschinsky transformation can be reliably applied.

The initial-state harmonic eigenfunctions are written in terms of Gaussian factors and Hermite polynomials

16 $$\left\langle \mathrm{Q̅}\right|{\mathrm{\psi ̅}}_{k}\rangle =\frac{(\det\mathrm{\Omega ̅}{)}^{1/4}}{\pi^{N/4}}\exp\left(-\frac{1}{2}{\mathrm{Q̅}}^{T}\mathrm{\Omega ̅}\mathrm{Q̅}\right)\prod_{i}\frac{1}{\sqrt{2^{k_{i}}k_{i}!}}H_{k_{i}}(\left({\mathrm{\Omega ̅}}^{1/2}\mathrm{Q̅}{)}_{i}\right)$$

After inserting eqs 12 and 16, together with the Duschinsky relation, into the coordinate representation of χ_*if*_^11^(*t*), one obtains a linear combination of auxiliary integrals

17 $${\mathrm{\chi ̃}}_{\mathrm{pq}}\left(t\right)=\int d\overset{®}{\mathrm{Q̅}}d{\overset{®}{\mathrm{Q̅}}}^{\prime }e^{-\Phi \left(\overset{®}{\mathrm{Q̅}},{\overset{®}{\mathrm{Q̅}}}^{\prime };t\right)}\prod_{j}{\left({{\overset{®}{\mathrm{Q̅}}}^{\prime }}_{j}\right)}^{p_{j}}\prod_{k}{\left({\overset{®}{\mathrm{Q̅}}}_{k}\right)}^{q_{k}}$$

where $\Phi \left(\overset{®}{\mathrm{Q̅}},{\overset{®}{\mathrm{Q̅}}}^{\prime };t\right)$ is a quadratic form collecting the contributions from the propagator and the two harmonic wave functions. The multi-indices ***p*** and ***q*** encode the polynomial degrees of the wave functions, with each component determined by the corresponding mode occupation number. The vibrational quantum numbers enter only through the polynomial prefactor, which makes the reduction especially suitable for automated symbolic treatment.

### 2.2 Gaussian-Moment Factorization

The central simplification is obtained by transforming eq 17 into sum and difference coordinates

18 $$Z=\frac{1}{\sqrt{2}}\left(\overset{®}{\mathrm{Q̅}}+{\overset{®}{\mathrm{Q̅}}}^{\prime }\right)$$

19 $$U=\frac{1}{\sqrt{2}}\left(\overset{®}{\mathrm{Q̅}}-{\overset{®}{\mathrm{Q̅}}}^{\prime }\right)$$

This separates the quadratic form into two Gaussian kernels, with matrices ***C*** and ***D*** and displacement vector ***v***

20 $$C=\overset{®}{\mathrm{\Omega ̅}}\coth\left(i\frac{\overset{®}{\mathrm{\Omega ̅}}t}{2}\right)+J^{T}\mathrm{\Omega ̅}J$$

21 $$D=\overset{®}{\mathrm{\Omega ̅}}\tanh\left(i\frac{\overset{®}{\mathrm{\Omega ̅}}t}{2}\right)+J^{T}\mathrm{\Omega ̅}J$$

22 $$v=J^{T}\mathrm{\Omega ̅}K$$

Completing the square in the sum coordinate introduces the shifted coordinate

23 $$\xi =Z+D^{-1}v$$

After expanding the polynomial prefactors in **ξ** and ***U***, the auxiliary integrals reduce to finite sums of multivariate Gaussian moments of the form

24 $$\int \mathrm{dX}\exp\left(-\frac{1}{2}X^{T}\mathrm{AX}\right)\prod_{j}X_{j}^{n_{j}}$$

These moments, where ***A*** is the Gaussian kernel and *n*_*j*_ is the polynomial degree of coordinate *X*_*j*_, are evaluated analytically by Isserlis’ theorem,^30,31^ which expresses any even-order Gaussian moment as a sum over pairwise contractions

25 $$\int \mathrm{dX}\exp\left(-\frac{1}{2}X^{T}\mathrm{AX}\right)X_{i_{1}}\cdot \cdot \cdot X_{i_{m}}=\sqrt{\frac{{\left(2\pi \right)}^{N}}{\det A}}\sum_{P\in P_{I}}\prod_{\left(a,b\right)\in P}{\left(A^{-1}\right)}_{\mathrm{ab}}$$

where $I=\left\{i_{1},\cdot \cdot \cdot ,i_{m}\right\}$ and $P_{I}$ denotes the set of distinct pairings of the indices in I , and *P* is one such pairing. Odd-order moments vanish.

The algorithm applies this factorization symbolically at the level of the final tensor invariants, rewriting the target tensor in terms of low-rank building blocks (inverse covariance matrices, displacement vectors), replacing explicit high-rank correlation tensors. The generator emits a symbolic sum of tensor products, canonicalizes them, and passes the contraction graph to a numerical evaluator, recasting the derivation from a transition-specific tensor-algebra problem into an automated symbolic contraction.

All scalar prefactors from the Mehler kernel, the harmonic wave functions, and the Gaussian integrations can be collected into a single complex prefactor

26 $$Y=\sqrt{\frac{\det\overset{®}{\mathrm{a̅}}\det\mathrm{\Omega ̅}}{\det C\det D}}$$

### 2.3 Fixing the Phase Discontinuities

Because the prefactor *Y* in eq 26 contains the square root of a complex determinant, its evaluation is susceptible to phase discontinuities. The time-dependent matrices involve trigonometric functions that hit poles at integer multiples of half-periods of the excited-state frequencies. As the determinant evolves, its argument crosses the branch cut, producing a discontinuous sign change. This introduces spurious oscillations and unphysical artifacts, as noted by Toutounji.^32^

Earlier implementations addressed the branch-cut problem through imposing phase continuity numerically: tracking the phase of the prefactor along the time grid and flipping its sign when a branch crossing is detected.^13^ While effective in moderate parameter regimes, such heuristics become fragile for large systems with high-frequency modes or nonuniform integration grids, where the phase can evolve by more than π within a single integration step.

Instead of tracking the branch cut numerically, we apply an algebraic factorization to isolate and exactly resolve the singularities in a way similar to what was proposed by Changala et al.^33^ By rewriting the prefactor matrices in terms of the unitary time-evolution matrix of the excited state, $V=\exp\left(-i\overset{®}{\mathrm{\Omega ̅}}t\right)$ , the singular denominators stemming from the Mehler kernel exactly cancel out. The squared prefactor reduces to

27 $$Y^{2}\left(t\right)=\frac{\det\left(2\overset{®}{\mathrm{\Omega ̅}}\mathrm{\Omega ̅}\right)}{\det(A+\overset{®}{\mathrm{\Omega ̅}}{)}^{2}}\frac{\det\left(V\right)}{\det\left(I-\mathrm{WV}\right)\det\left(I+\mathrm{WV}\right)}$$

with

$$A=J^{T}\mathrm{\Omega ̅}J$$

and $W=(A+\overset{®}{\mathrm{\Omega ̅}}{)}^{-1}\left(A-\overset{®}{\mathrm{\Omega ̅}}\right)$ .

Defining the time-independent constant

28 $$\kappa =\frac{\det\left(2\overset{®}{\mathrm{\Omega ̅}}\mathrm{\Omega ̅}\right)}{\det(A+\overset{®}{\mathrm{\Omega ̅}}{)}^{2}}$$

the prefactor can be written as

29 $$Y\left(t\right)=\sqrt{\kappa }e^{-i\sum_{k}{\overset{®}{\mathrm{\omega ̅}}}_{k}t/2}R\left(t\right)$$

with the residual factor

30 $$R\left(t\right)=\frac{1}{\sqrt{\det\left(I-\mathrm{WVWV}\right)}}$$

The explicit zero-point phase is therefore isolated in the exponential, while the remaining branch issue is confined to *R*(*t*) that can be evaluated continuously by taking the principal square root of each eigenvalue factor before multiplying them (proof is given in Appendix A).

### 2.4 Reduced-Scaling Construction

The practical consequence of the factorized formulation is that the response tensor can be assembled without explicitly forming the full high-rank correlation object associated with a given transition class. In a conventional implementation, a given term might be written schematically as

31 $$T_{\mathrm{jk},\rho \eta }\leftarrow \sum_{i,l}P_{i\rho }^{\prime }X_{\mathrm{ijkl}}P_{l\eta }^{\prime }$$

where *X*_*ijkl*_ is a rank-4 intermediate generated from the correlation function. ***P*** ′ is shortened notation for the first derivative of either ***P*** ^*A,e*^ or ***P*** ^*B,e*^ with respect to the normal coordinates. Building and storing such intermediates requires $O\left(N^{4}\right)$ memory and becomes rapidly prohibitive as the vibrational rank increases.

Our approach avoids constructing *X*_*ijkl*_ (or any explicit high-rank intermediate) by operating directly on the factorized invariant-level representation before numerical evaluation. The algorithm proceeds in three stages.

#### Stage 1: Symbolic Contractions

Substituting the Hermite-polynomial expansion into eq 17 and completing the square as described above gives Gaussian moments of the form eq 24 in the shifted coordinates

32 $$\int d\xi \mathrm{dU}\exp\left(-\frac{1}{2}\xi^{T}D\xi \right)\exp\left(-\frac{1}{2}U^{T}\mathrm{CU}\right)\prod_{j}\xi_{j}^{w_{j}}\prod_{k}U_{k}^{u_{k}}$$

multiplied by displacement factors from ***D*** ^–1^ ***v***. Each integral is evaluated by Isserlis’ theorem, which expresses even-order Gaussian moments as sums over perfect index pairings. We enumerate the matchings, each one replaces contracted index pairs with entries of ***D*** ^–1^ or ***C*** ^–1^. The result encodes all contributions as products of ***D*** ^–1^, ***C*** ^–1^, the displacement vector ***D*** ^–1^ ***v***, and property derivatives *P*_*iρ*_^′^.

#### Stage 2: Canonicalization of the Addends

The symbolic expression from Stage 1 may contain many addends that share the same contraction topology but differ in the labels assigned to internal (dummy) indices. To combine these, we canonicalize each addend by relabeling dummy indices according to the order in which they first appear, then sorting the tensor factors lexicographically by name and index tuple. Two addends with identical canonical forms represent the same contraction graph and are merged by summing their coefficients. This isomorphism-based simplification reduces the number of distinct terms significantly. In the code, this step is carried out directly on the symbolic addends before property contraction and numerical evaluation.

#### Stage 3: Optimized Numerical Contraction

The canonicalized expression is translated into a sum of Einstein contractions, each specified by the tensor names and symbolic index labels. For each addend, an optimized contraction path is determined with a path-planning algorithm. The simplified symbolic equations and the corresponding per-addend contraction plans can be cached for each transition class, so repeated evaluations avoid both symbolic regeneration and path optimization. Because many addends share common subexpressions—for example, the contraction ∑_*i*_ *P*_*iρ*_^′^(*D* ^–1^)_*ij*_ may appear in several addends with different outer labels—the evaluator can also reuse numerical intermediates. When memory permits, intermediate contractions are identified by canonical signatures based on the factor names, index structure, and output labels; matching intermediates are computed once within a quadrature chunk and reused across addends. This caching acts only on the low-rank objects entering the factorized expression and never requires forming transition-specific high-rank correlation tensors.

### 2.5 Finite-Temperature Extension

At finite temperature, the observed RR spectrum is obtained by summing over all thermally populated initial vibrational states

33 $$I\left(\omega_{I},T\right)=\frac{1}{Z\left(T\right)}\sum_{v}e^{-E_{v}/k_{B}T}\sum_{v^{\prime }}I_{v\to v\prime }\left(\omega_{I}\right)$$

where ***v*** denotes the initial occupation vector, *E*_*v*_ its vibrational energy, and *Z*(*T*) the partition function. The difficulty lies not only in the number of populated initial states, but also on the need to generate the corresponding transition tensors for hot bands and other excited-state manifolds.

Within the present framework, the occupation vector enters through the polynomial structure of the harmonic wave functions and therefore through the set of Gaussian moments to be generated. The code can thus produce the required expressions for any | ***v*** ⟩ → | ***v*** ′⟩ transition using the same workflow as fundamentals, overtones, combination bands from the ground state, removing the need for transition-specific manual derivations. As a result, the main practical limitation is the convergence of the Boltzmann sum.

## 3 Computational Details

Structural optimizations and frequency calculations for the ground and excited electronic states were done at the density functional theory (DFT) and its time-dependent extension (TD-DFT) level, using the Gaussian^34^ suite of quantum chemical programs.

The vertical Hessian (VH) model was preferred to compute the RR and RROA vibronic transition integrals, which means that the excited-state harmonic force constants were computed at the ground-state equilibrium geometry. This choice is motivated by the fact that the vibrational overlaps are more accurately described in VH than in the alternative adiabatic Hessian representation where the excited-state frequencies are computed at a displaced minimum. The trade-off on the accuracy of the vibrational energies is not as critical in RR as it can be in one-photon absorption or emission spectra considering that the damping factors typically used are larger than the expected errors on the frequencies. However, since the excited-state PES is computed out of equilibrium, VH is also more susceptible to the anharmonicity and imaginary frequencies can be found at the ground-state geometry. In this case, AH was used instead, which means that the excited-state geometry was first optimized and the harmonic frequencies computed at this point. It should be noted that, in all cases where AH was employed here, the excited-state PES minima were found very close to the vertical region, so the displacements were very small, so errors in the computation of integrals intensities are not expected to differ much from the vertical approach.

Based on initial tests with reference implementations, the TD Laplace transform was evaluated with the exponential mapping *y* = exp­(−*γt*) on a grid of 1000 points, which was deemed sufficient to achieve convergence based on the results from the validation tests. All calculations were done using the FCHT representation of the transition moments.

TD RR calculations were done with a newly developed, open-source Python package, ARIES (Automated resonance Raman Intensity Evaluation Suite). Symbolic manipulations use SymEngine,^35^ whereas the numerical contractions are formulated in Einstein-summation form and can be evaluated with NumPy^36^ or opt_einsum^37^ for contraction-path optimization.

Anthracene (panel A in Figure 1) was used for the validation of our implementation. All calculations were done in gas phase at the ωB97X/def2-TZVP level, targeting the lowest bright state, at 221.91 nm (*S*_4_). The RR calculations used γ = 500 cm^–1^ and an incident energy in resonance with the transition between vibrational ground states (0–0), at 43924.36 cm^–1^. The spectra were plotted using Gaussian distribution functions with half-widths at half-maximum (HWHMs) of 10 cm^–1^. For the reference calculation, the TI implementation in Gaussian was used,^15^ with the default parameters. The convergence on one-photon absorption spectra was first checked to ensure that the prescreening algorithm used to truncate the infinite summation over the intermediate vibrational states properly captured all relevant transitions and that full convergence was achieved (above 99.94%).

**Figure 1:**
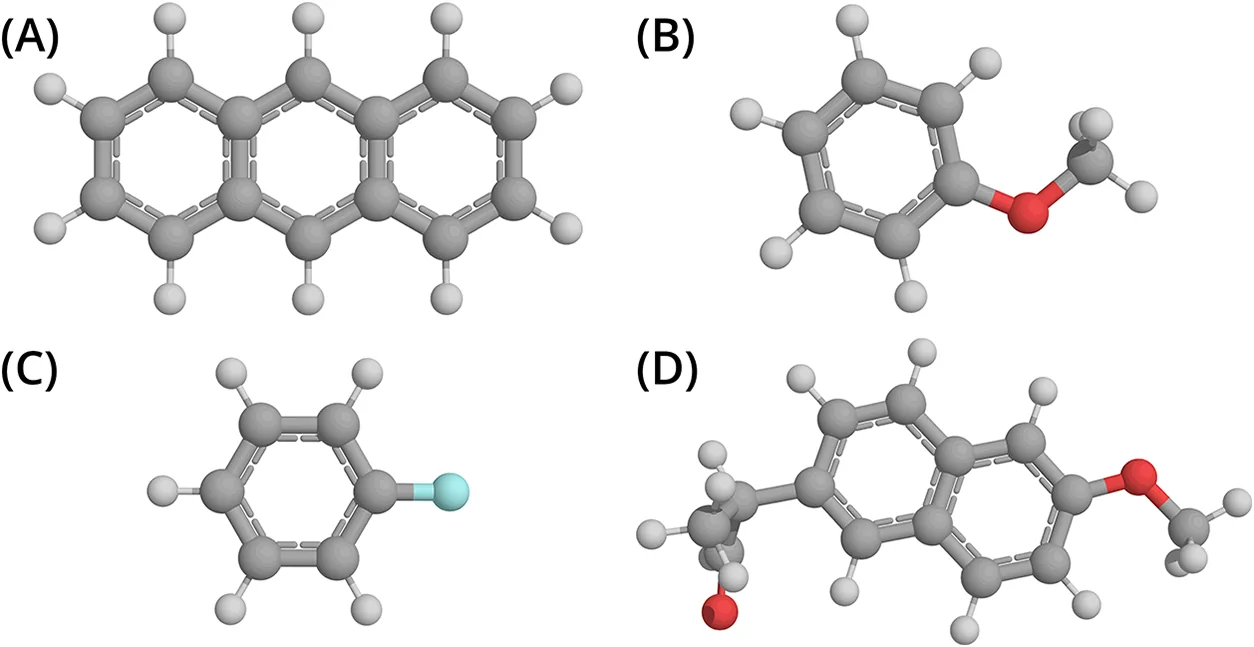
Molecular structures of the systems investigated in this work: (A) anthracene, (B) anisole, (C) fluorobenzene, and (D) (*S*)-(+)-naproxen.

As a secondary test case to validate our implementation, anisole (panel B in Figure 1) was used. Computations were done at the ωB97X/def2-TZVP level of theory. An imaginary frequency being present in the excited state at the initial-state geometry, an optimization was carried out and the AH framework used instead of VH. The incident wavelength was set in resonance with the 0–0 transition. Reference TI calculations were done with the code implemented in Gaussian. Default parameters for the prescreening scheme were used initially. Because of the higher degree of mode mixing compared to anthracene, this was not always sufficient to ensure that all relevant intermediate states were included. To remediate, the prescreening parameters were incremented (see refs 38–40 for details on the algorithm), using the one-photon absorption spectra involving the initial and final states as references.

To analyze the algorithmic cost, a scaling benchmark was developed using synthetic inputs instead of molecular electronic-structure data. Each benchmark input was built from randomly generated orthogonal Duschinsky matrices, displacement vectors, positive initial- and intermediate-state frequencies, a fixed zero-point energy difference of 44,000 cm^–1^, γ = 500 cm^–1^, and random transition dipoles and derivatives. We tested *N* = 96, 128, 192, 256, 384, 512, 768, and 1024 modes with three independent repeats for each value of *N*, using a single processor thread. To avoid the risk of inconsistency and to keep the benchmark timings directly comparable, intermediate caching was not enabled during these runs.

For the analysis of finite-temperature effects, fluorobenzene (panel C in Figure 1) was chosen as a test case, using CAM-B3LYP/def2-TZVP. An imaginary frequency being found in the excited state at the initial-state geometry, AH was used. For this system, the reference incident energy was set in resonance with the 0–0 transition, at 42 039.11 cm^–1^. The fluorobenzene RR tensors were evaluated at 300 K with Lorentzian broadening functions and HWHMs of 10 cm^–1^. To probe the role of resonance conditions, we compared spectra computed with γ = 50 cm^–1^ and 500 cm^–1^ with incident frequencies shifted by ±500 cm^–1^ relative to the 0–0 resonance.

For (*S*)-(+)-naproxen (panel D in Figure 1), we considered two close intermediate states, *S*_1_ and *S*_2_, at the CAM-B3LYP/def2-TZVP level, with vertical excitation energies from the equilibrium of the ground state at 291.86 and 264.34 nm, respectively. The RROA calculations were carried out with γ = 500 cm^–1^ and incident frequencies tuned to the *S*_1_ resonance, to the midpoint between the two states (33 296.87 cm^–1^), and to the *S*_2_ resonance. Empirical broadening was done by means of Lorentzian functions and half-widths at half-maximum of 10 cm^–1^.

## 4 Results and Discussion

### 4.1 Validation of the Algorithm

In order to check the implementation of our autogenerator, we chose anthracene (panel A in Figure 1) and the existing time-independent implementation of RR in Gaussian as reference. The choice of anthracene is motivated by its high rigidity. This translates into limited mode mixing associated with the electronic transitions of interest and small structural deformation, as evidenced by the near-diagonal Duschinsky matrix and low-amplitude shift vector (Figure S1 of the Supporting Information). This property has made polycyclic aromatic hydrocarbons standard prototypical systems to test new models for vibronic calculations.^41–43^ Experimental absorption spectra to the lower electronic states show well-resolved vibronic bands, generally caused by vibrational progressions of a limited number of modes. Since the number of transitions actually contributing is rather low, full convergence of sum-over-states methods is straightforward. The choice of using TI RR as reference among existing implementations stems from the facility of this approach to handle any type of transitions, including nonfundamental transitions and hot bands. An important aspect to keep in mind here is that practical implementations require some reduction to a finite number of the manifold of intermediate states, and thus can fail to capture all relevant contributions, so that the overall performance can vary depending on the target system, as well as the nature of the initial and final states. It is thus important to check that the truncation is done meaningfully. Based on the convergence achieved with one-photon absorption at *T* = 0 K, we do not expect any problem with convergence for transitions from low-quanta states. To limit the numerical noise, the Raman spectra were computed in resonance with the first bright state. Based on the vertical excitation energies and oscillator strengths (Table S1 in the Supporting Information), *S*_4_ was thus chosen.

We first compare the TD and TI results visually, considering the spectral band-shape at *T* = 0 K with transitions up to two quanta, that include all fundamentals (|0⟩ → |1_*i*_⟩, simply noted 0 → 1_*i*_), first overtones (0 → 2_*i*_) and “1 + 1” combinations (0 → 1_*i*_1_*j*_). The results are shown in Figure 2, with the band-shapes associated with each type of transitions reported in the Supporting Information, in Figures S2 (fundamentals), S3 (overtones) and S4 (combination bands).

**Figure 2:**
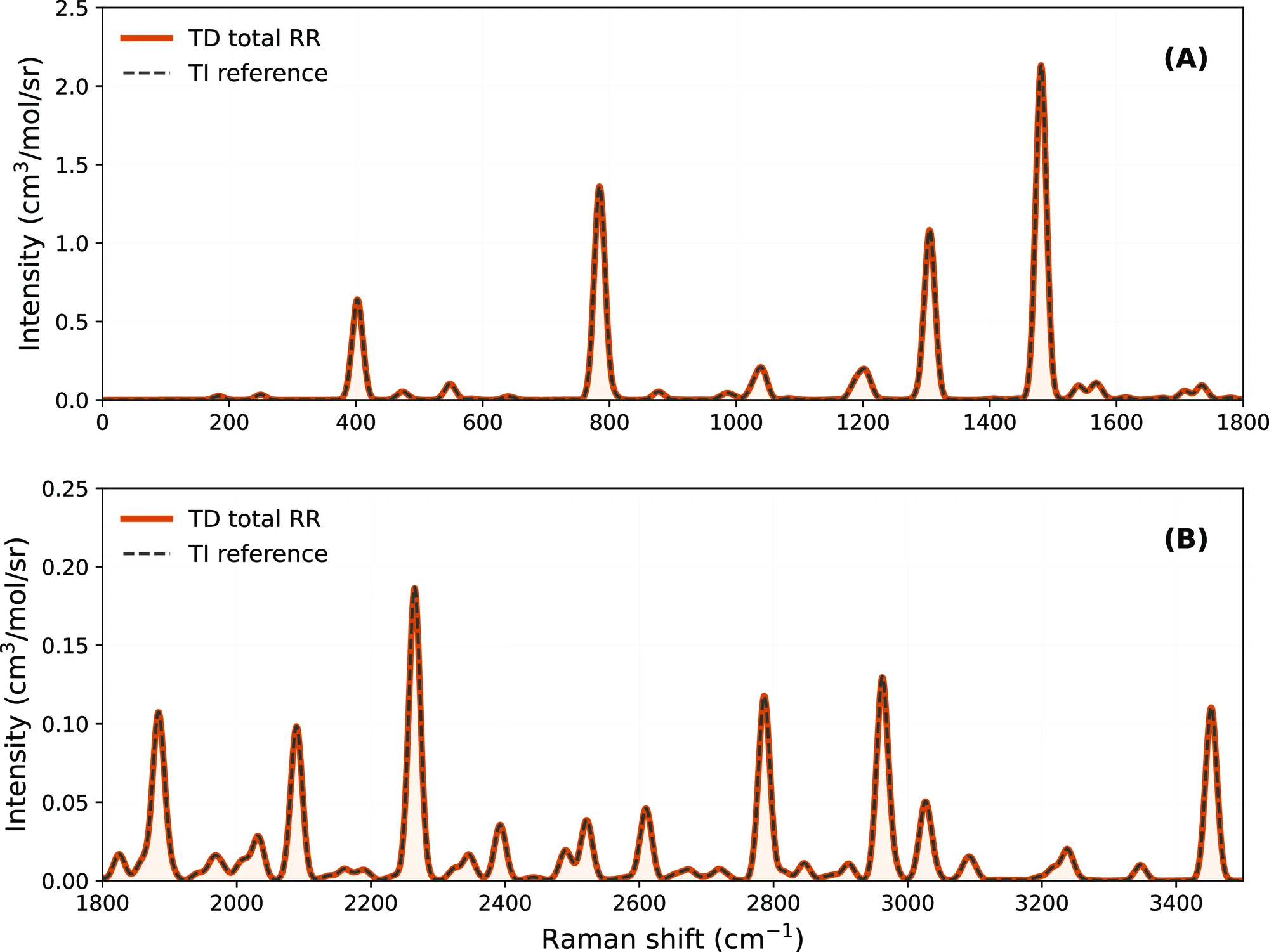
Anthracene TI and TD RR spectrum with incident light set at 43924.36 cm^–1^ at the VH|FCHT level. Gaussian distribution functions with HWHMs of 10 cm^–1^ were used for the broadening. The fingerprint (A) and higher-energy regions (B) were split and scaled appropriately for better visibility.

We note an excellent agreement between the TD and TI results over the whole vibrational range up to the CH stretching region, even in lower-intensity regions. The results are also independent of the type of transition. Looking separately at fundamentals, overtones, and combination bands (Figures S2–S4 in the Supporting Information), the TD and TI curves remain superimposed. The strongest anthracene fundamentals, at 784 cm^–1^, 1305 cm^–1^ and 1481 cm^–1^, corresponding to modes 19, 43 and 48, match perfectly, and their overtone progressions as well, while no TD/TI discrepancies can be noted over the dense combination manifold, which populates the expected intermediate-frequency regions.

After this initial validation, we switch to numerical comparisons, considering all transitions with an intensity above 10^–4^ cm^3^ mol^–1^ sr^–1^ (see Table S2 in the Supporting Information for details). For fundamentals, the mean absolute error (MAE) in relative intensity is Δ*I*/*I* = 0.09% (maximum 0.20%). For overtones, the MAE in relative intensity is Δ*I*/*I* = 0.21% (max. 1.15%). Finally, for combination bands, it is Δ*I*/*I* = 0.26% (max. 2.07%). This comparison confirms that the numerical Laplace transform applied to the optimized symbolic contractions systematically yields near-exact agreement with the formal analytic equations underlying the reference TI implementation, for both sparse and highly congested transition manifolds. A slight deviation is natural considering the numerical truncations of the data read in input by our generator compared to the full internal data flow used for TI within Gaussian. Moreover, the convergence is slightly worse for TI with integrals from 2-quanta states to the manifold of intermediate states. For the cases with the maximum errors, to |2_45_⟩ for the first overtones, and |1_8_1_66_⟩ for the combinations, the total progression on OPA from these states is 99.34% and 98.87%, respectively, compared to 99.94% from the ground state.

Let us now switch to hot-band transitions. For this part of the validation, we considered initial states containing one vibrational quantum in a single mode. From each such initial state, we generated the hot-band classes 1_*i*_ → 2_*i*_, 1_*i*_ → 3_*i*_, 1_*i*_ → 1_*i*_ 1_*j*_, 1_*i*_ → 2_*i*_ 1_*j*_ and 1_*i*_ → 1_*i*_ 2_*j*_, retaining the 100 most intense transitions in each class for comparison with the TI reference. The result is shown in Table S3 of the Supporting Information and the three most intense transitions of each class are reported in Table 1.

**Table 1:** Three Most Intense Anthracene Hot-Band Transitions in Each Class Used for TD/TI Comparison^a^

Class	TD transition	ν	*I* ^TD^	*I* ^TI^
1_*i*_ → 2_*i*_	|1_48_⟩ → |2_48_⟩	1481.01	57.309	57.297
	|1_19_⟩ → |2_19_⟩	784.12	41.255	41.275
	|1_43_⟩ → |2_43_⟩	1305.16	36.907	36.913
1_*i*_ → 3_*i*_	|1_13_⟩ → |3_13_⟩	1204.48	10.486	10.449
	|1_5_⟩ → |3_5_⟩	548.53	5.995	6.0013
	|1_48_⟩ → |3_48_⟩	2962.02	5.256	5.2796
1_*i*_ → 1_*i*_ 1_*j*_	|1_31_⟩ → |1_31_1_48_⟩	1481.01	47.591	47.491
	|1_32_⟩ → |1_32_1_48_⟩	1481.01	47.540	47.403
	|1_23_⟩ → |1_23_1_48_⟩	1481.01	47.328	47.243
1_*i*_ → 2_*i*_ 1_*j*_	|1_19_⟩ → |2_19_1_48_⟩	2265.13	5.576	5.590
	|1_48_⟩ → |2_48_1_19_⟩	2265.13	3.929	3.934
	|1_8_⟩ → |2_8_1_48_⟩	1883.24	3.682	3.684
1_*i*_ → 1_*i*_ 2_*j*_	|1_31_⟩ → |1_31_2_13_⟩	1204.48	3.640	3.586
	|1_32_⟩ → |1_32_2_13_⟩	1204.48	3.624	3.561
	|1_18_⟩ → |1_18_2_13_⟩	1204.48	3.616	3.550

a Vibrational energies ν are reported in cm^–1^, the intensities *I* in cm^3^ mol^–1^ sr^–1^.

The agreement between TD and TI is consistently good for all the transitions listed in Table 1. Over the whole set in Table S3 (the Supporting Information), only 34 transitions show a TD/TI difference above 1% with the worst agreement being |1_11_⟩ → |2_11_ 1_2_⟩ at 5.24%. To check if the problem could be originating from the prescreening algorithm used to truncate the summation over the intermediate states in the time-independent formalism, new parameters were used, higher than the default values (*C*_1_^max^ = 20, *C*_2_^max^ = 13, *N*_*I*_^max^ = 10^8^). By design, such increase translates systematically into newer states including without excluding any of the ones selected with lower thresholds, which should mechanically improve the convergence.^38,40^ Based on these considerations, three new configurations were created, denoted L, XL, and XXL, and defined as follows, *C*_1_^max^ = 40 and *C*_2_^max^ = 30 with effective maximum integral counts of 10^9^, 2 × 10^9^ and 4 × 10^9^, respectively.^40^ At the highest level (XXL), all hot-band transitions in the validation set fall below a 1% TD/TI difference. This confirms that the symbolic TD formulation reproduces the reference TI intensities not only for progression-like channels such as 1_*i*_ → 2_*i*_ and 1_*i*_ → 3_*i*_, but also for mixed manifolds such as 1_*i*_ → 1_*i*_ 1_*j*_, 1_*i*_ → 2_*i*_ 1_*j*_, and 1_*i*_ → 1_*i*_ 2_*j*_, provided that the TI intermediate-state summation is sufficiently converged.

The raw intensities also show that these hot-band channels are not intrinsically weak. The strongest fundamental anthracene lines reach 45.392, 27.787, and 22.993 cm^3^ mol^–1^ sr^–1^ for the 0 → 1_*i*_ transitions at 1481.01 cm^–1^, 784.12 cm^–1^ and 1305.16 cm^–1^, respectively. Against this reference, the leading 1_*i*_ → 2_*i*_ peaks reach 57.309, 41.255, and 36.907 cm^3^ mol^–1^ sr^–1^, while the strongest 1_*i*_ → 1_*i*_ 1_*j*_ lines cap at 47.591, 47.540, and 47.328. These values are therefore comparable to, and in some cases larger than, the strongest fundamental transitions in anthracene. However, these data do not account for the populations of the initial state. For instance, the population of |1_5_⟩ is 26.74% that of the ground state at *T* = 300 K, and |1_48_⟩ is 0.08%.

This is particularly informative for the thermal problem, since it means that if the population of thermally excited states is not negligible, some transitions from these states are likely to be influential; indeed, hot-band transitions can carry intensities of essentially the same order as equivalent transitions from the ground state.

To confirm that our implementation performed well also in the presence of stronger mode mixing, we considered a second molecule, anisole. A previous study on the Franck–Condon one-photon absorption spectrum had shown very good performance of the TI framework compared to the well-resolved resonance enhanced multiphoton ionization (REMPI) experimental spectrum.^44^ Compared to anthracene, we observe a higher degree of mode mixing (Figure S5 in the Supporting Information), which can impact negatively the quality of the prescreening to truncate the summation over the intermediate states.

For the TI reference data to be meaningful, all non-negligible transitions *i* → *m* and *f* → *m* must be included in the computation of the RR polarizability tensor. Since this cannot be evaluated formally for RR, an alternative path hinted in the study of anthracene is to look at the convergence—calculated as the total computed intensity divided by the analytic value obtained from sum rules—of the OPA spectra originating from the initial *i* and final *f* states. For a RR transition *i* → *f*, we define

34 $$\eta =\min\left(\eta_{i},\eta_{f}\right)$$

where η_*i*_ and η_*f*_ are the OPA convergences from the initial and final vibrational states. A large η indicates that the intermediate-state manifolds relevant to the calculation of the polarizability tensor were properly included, making the TI RR reference reliable. The degree of mode mixing was monitored through $\max_{k}\left(J_{ik}^{2}\right)$ , where lower values indicate stronger mixing.

Like for anthracene, the most intense transitions were selected for each class, up to 100 per class and with values of at least 10^–4^ cm^3^ mol^–1^ sr^–1^. The full list of 334 transitions is collected in Table S5 in the Supporting Information. This set includes ground-state fundamentals, overtones and combination bands, as well the hot-band/progression classes 1_*i*_ → 2_*i*_, 1_*i*_ → 3_*i*_, 1_*i*_ → 1_*i*_ 1_*j*_, 1_*i*_ → 2_*i*_ 1_*j*_, and 1_*i*_ → 1_*i*_ 2_*j*_. Across the full set, the mean TD/TI difference with default TI prescreening is 0.56% and the median is 0.20%, showing that the TD implementation reproduces the TI reference for the large majority of transitions. As expected the largest deviations, which reach up to 9.94%, are concentrated in transitions with lower OPA convergence and stronger mode mixing.

To separate TD errors from TI truncation errors, the problematic transitions were recomputed with the L and XL TI prescreening levels. Since the largest errors were significantly higher than for anthracene, the impact of the prescreening algorithm was monitored, considering the OPA spectra as reference. Among the transitions where the RR TD/TI difference was above 1% with the default prescreening parameters, 10 reached η ≥ 95% with the L configuration. For these ten transitions, the L and XL RR calculations reduce all TD/TI differences below 1% (Table S6); the mean error decreases to 0.49% at L and to 0.35% at XL, with maximum errors of 0.65% and 0.44%, respectively. These results confirm that the larger deviations are to be primarily ascribed to the incomplete TI prescreening rather than to a limitation of the TD formulation.

As a further illustration on the problem of convergence, we then built an additional validation set focused on modes with larger mixing. Two criteria were used here. Only transitions with an intensity above 10^–4^ cm^3^ mol^–1^ sr^–1^ were retained again here. The mode-mixing index associated with each transition was computed as the lowest value of $\max_{k}\left(J_{ik}^{2}\right)$ among all modes involved in the initial or final state. For each set, the transitions were then sorted by increasing order of the mode-mixing index and the list truncated to the first 100. The L prescreening configuration reduced the mean TD/TI error from 1.43% to 0.49% and the maximum error from 5.83% to 2.81% (Table S7). Convergence with higher prescreening remains difficult, at the expense of a stark increase of the computational time. For instance, on the previous set, switching from the default to L configuration results in an increase of the computational time by a factor of 11.2 for each RR transition, and to XL by a further factor of 2.3.

This test confirms that the TD formulation remains stable for strongly mixed transitions. The residual discrepancies observed with default TI prescreening are therefore mainly attributable to the truncation of the intermediate-state sum rather than to a failure of the TD treatment of the Duschinsky transformation.

### 4.2 Computational Scaling

To test the scalability of our implementation, we first study the case of vibrational progressions of the form 0 → *n*_*i*_ (*n* = 1, 2, 3, 4), and then focus on the case of hot bands, starting from fundamentals (1_*i*_ → 1_*i*_ 1_*j*_ and 1_*i*_ → 2_*i*_) and then several cases involving more quanta or modes, namely 1_*i*_ → 3_*i*_, 1_*i*_ → 2_*i*_ 1_*j*_, 1_*i*_ → 1_*i*_ 2_*j*_, and 1_*i*_ → 1_*i*_ 1_*j*_1_*k*_.

The types of transitions have been chosen to distinguish the effect of formal contraction order from the growth in the total number of transitions corresponding to a given type. The number of modes was progressively increased from *N* = 96 up to 1024. To get statistically relevant results, each calculation was run three times.

The primary metric is the total execution time on a single processor. The results are reported in Figure 3, together with a cubic polynomial fitting for reference. All transition classes require a common set of dense linear-algebra operations, including the factorization or inversion of the Gaussian kernels entering the low-rank expression. These operations introduce a formal $O\left(N^{3}\right)$ baseline with respect to the number of normal modes, but they are evaluated through standard optimized linear-algebra kernels and carry a small prefactor.

**Figure 3:**
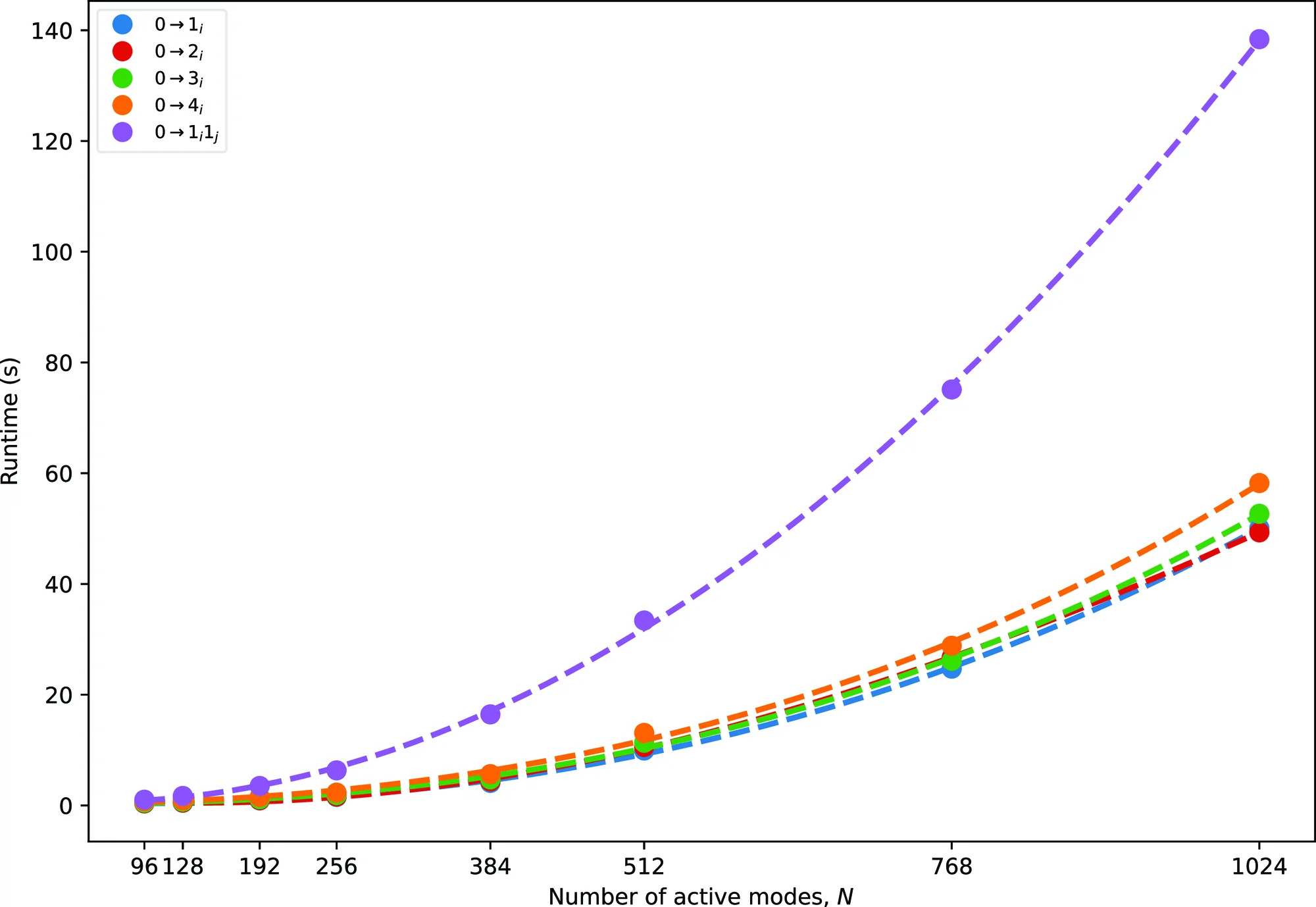
Total computational time on a single processor (in s) as a function of the total number of normal modes for the 0 → *n*_*i*_ transitions (*n* = 1,2,3,4) and “1 + 1” combinations from the ground state. The markers report the average runtimes over multiple executions and dashed lines are cubic fits in *N*.

This common baseline explains why the 0 → *n*_*i*_ progressions remain closely grouped over the sampled range. Increasing *n* changes the degree and number of terms in the generated expressions, but it does not introduce a large increase in the number of final transitions. The 0 → 1_*i*_1_*j*_ combination manifold is more expensive because all binary combinations must be evaluated, yet it still benefits from the same low-rank construction.

This behavior contrasts with explicit high-rank formulations, such as that of de Souza et al.,^14^ where the construction and contraction of the correlation tensor lead to a formal $O\left(N^{6}\right)$ cost. In the present formulation, these high-rank tensors are never materialized, which greatly reduces the cost for a broad range of higher-order transitions.

Let us focus now on hot-bands, reported in Figure 4. The set of transitions for each group contains *N* starting states, from which all possible transitions matching the class definition are built. To be consistent with physical processes, only progressions are considered, which means for instance that the final state contains at least the initial-state configuration, with possibly additional excited modes or a higher number of quanta for modes already excited in the initial state.

**Figure 4:**
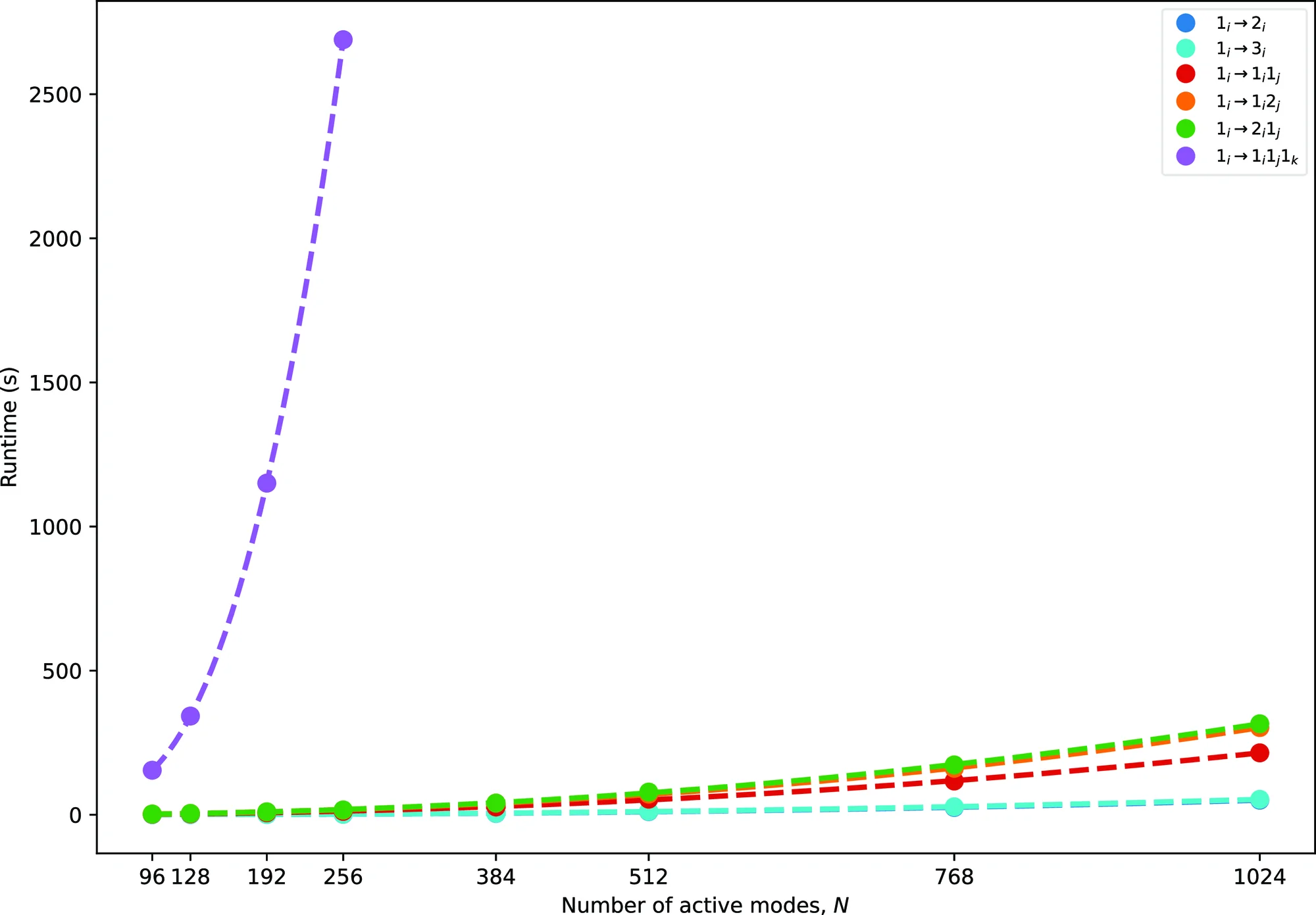
Total computational time on a single processor (in s), as a function of the total number of normal modes considering all possible transitions in a class from fundamental states. The markers report the average runtimes over multiple executions and dashed lines are cubic fits in *N*.

We obtain the same qualitative trends as from the ground state: single-mode channels 1_*i*_ → 2_*i*_ and 1_*i*_ → 3_*i*_ remain the cheapest, while the cost of denser two-mode manifolds such as 1_*i*_ → 1_*i*_ 1_*j*_, 1_*i*_ → 1_*i*_ 2_*j*_, and 1_*i*_ → 2_*i*_ 1_*j*_ are lifted by larger prefactors. The progression of the computational cost with the number of modes remains modest for all classes except 1_*i*_ → 1_*i*_ 1_*j*_1_*k*_. This class is qualitatively different from those considered before. While the growth remains cubic with the number of normal modes, the observed cubic trend has a different origin from the common dense linear-algebra baseline: here, the large prefactor is caused by the number of the transitions.

To summarize this analysis, the observed runtime is governed, for most transition classes, by the low-rank algebraic evaluation, which combines a common small-prefactor cubic dense-linear-algebra baseline with transition-specific generated contractions. The main exception is represented by sufficiently dense hot-band manifolds, such as 1_*i*_ → 1_*i*_ 1_*j*_1_*k*_, where the number of individual transitions becomes the practical limiting factor. Compared to explicit high-rank formulations commonly used in existing implementations, the present approach therefore remains favorable across a broad range of higher-order transitions. This is especially critical when considering the finer structure of Raman or ROA spectra outside of fundamental regions, or when considering temperature effects, where the number of transitions to include can grow very quickly. In the latter case, the limiting factor in terms of computational cost and feasibility is the total transitions count, which causes the overall computations to leave the regime of small cubic prefactors.

### 4.3 Temperature Effects in Fluorobenzene

To study the temperature effects, we chose as a test case fluorobenzene, represented in panel C of Figure 1. The molecule is relatively simple, and its vibrational frequencies are sufficiently high that the number of thermally populated states at room temperature remains manageable (see Table S8 in the Supporting Information for a listing of the harmonic frequencies). The PES of the first excited state has an imaginary frequency (*i*105.7 cm^–1^) in the vertical position. For this reason, a TD-DFT optimization was carried out and a close minimum found. The Duschinsky matrix shows a limited mode mixing, and the shift vector is rather small (Figure S6 of the Supporting Information). To simulate realistic conditions, the temperature was set to 300 K and all initial states with a population above 1% of the ground state’s were included.

The states were then classified according to the number of excited modes in the initial state. In this notation, order 0 contains only the vibrational ground state, order 1 contains singly excited initial states, and order 2 contains initial states with two excited modes. Under the present 300 K conditions, reaching the 1% population cutoff already requires including contributions up to order 2. This truncated ensemble accounts for 81.76% of the total canonical partition function. If all states up to order 2 are systematically included, without a specific cutoff based on the population, the coverage rises up to 92.00% (the corresponding spectrum is shown in Figure S8 in the Supporting Information), showing that the finite-temperature spectrum discussed here is still not fully converged. Nevertheless, it can be seen from a comparison of Figures 5 and S8 that temperature-related trends, as well as the effect of the damping factor, are already well reproduced with the truncation based on the Boltzmann population. Only three singly excited states have a population above 10% of that of the ground state (|1_1_⟩ at 31%, |1_2_⟩ at 14%, and |1_3_⟩ at 13%), but lower-populated order-2 states must still be included to obtain a reasonable description of the thermal ensemble.

**Figure 5:**
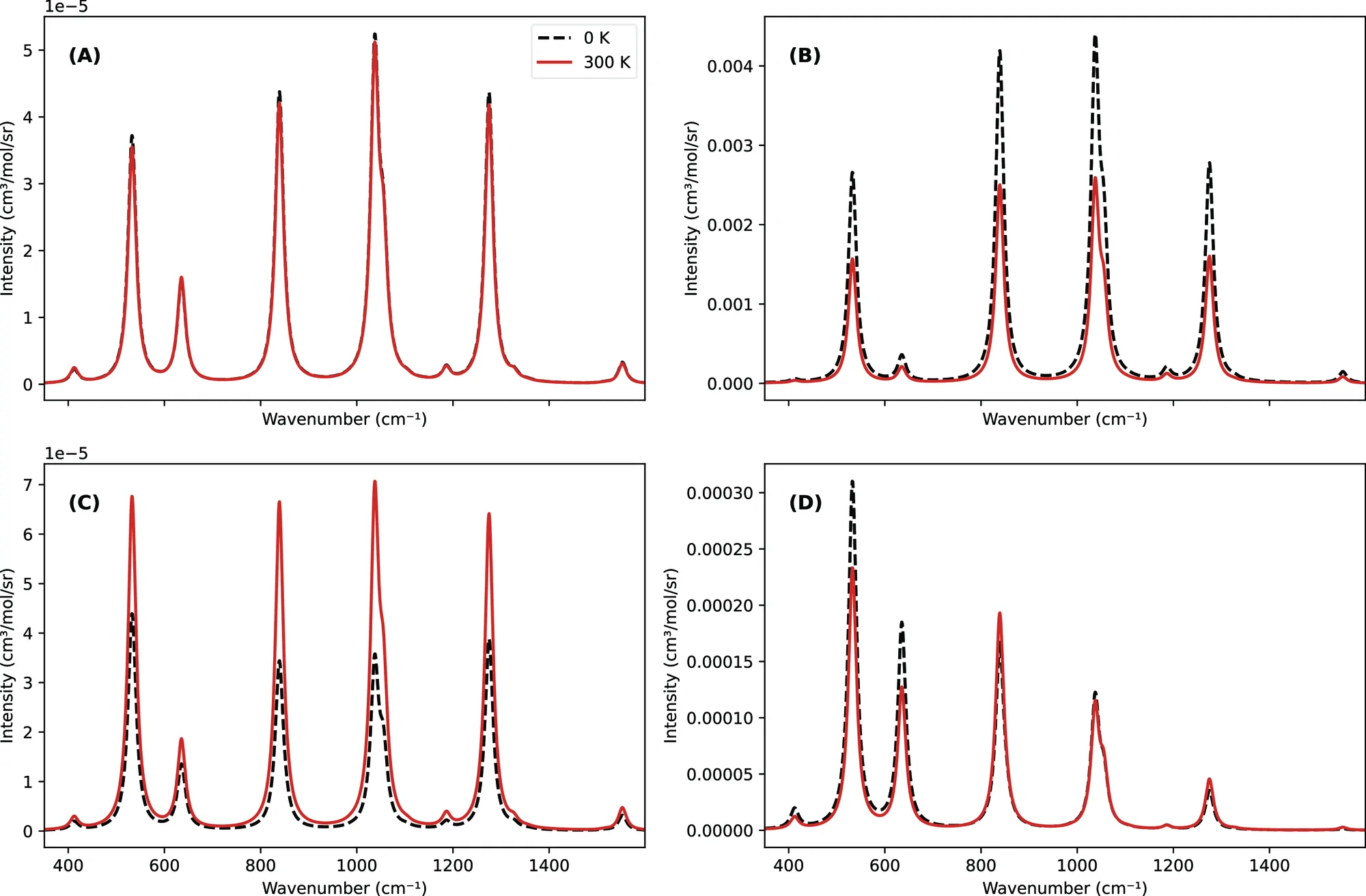
Fluorobenzene RR spectra comparing the 0 and 300 K spectra for four sets of resonance and damping parameters, using a 1% population cutoff for the thermal ensemble. In each panel, the dashed 0 K curve and the solid 300 K curve were evaluated with the same resonance parameters. (A) γ = 500 cm^–1^, incident frequency at the 0–0 resonance. (B) γ = 50 cm^–1^, incident frequency at the 0–0 resonance. (C) γ = 50 cm^–1^, incident frequency shifted by −500 cm^–1^. (D) γ = 50 cm^–1^, incident frequency shifted by +500 cm^–1^.

As discussed in the previous example, moving to higher excitation order rapidly increases both the number of transition classes and the number of individual transitions to be evaluated. In practice, beyond order 2 the computational cost departs significantly from the favorable cubic regime, because some hot-band classes lead to an effectively quartic growth in the number of transitions.

At first sight, the impact of temperature might be expected to remain limited, because of the rigidity of fluorobenzene. Indeed, the number of thermally populated states is still small, and most of the additional hot-band transitions, whose energies are computed at the harmonic-oscillator level, fall in spectral regions that are already dominated by the parent fundamentals. This is especially true for the intense bands near 533 cm^–1^, 839 cm^–1^, 1037 cm^–1^ and 1275 cm^–1^, where the finite-temperature contributions remain largely superimposed with the corresponding 0 → 1_*i*_ transitions.

As shown in Figure 5, the effects of temperature can be strongly influenced by the resonance conditions. For each panel, the dashed 0 K spectrum and the solid 300 K spectrum were computed with the same incident frequency and the same damping parameter, so that only the effect of thermal activation is being compared within a given resonance condition. When the damping is large (γ = 500 cm^–1^) and the incident frequency is kept at the 0–0 resonance, the 300 K spectrum remains close to the 0 K one, with only small local intensity redistributions. Shifting out of resonance (Figure S7 in the Supporting Information) does not change significantly this trend. By contrast, for the narrower value γ = 50 cm^–1^, the relative weight of the hot-band manifold becomes much more sensitive to detuning. At resonance, the 300 K spectrum is weaker than the 0 K one over most of the fingerprint region, whereas shifting the incident frequency by −500 cm^–1^ enhances the room-temperature spectrum, while shifting it by +500 cm^–1^ gives an intermediate, mode-dependent behavior.

Fluorobenzene therefore illustrates two complementary points. First, the number of initial states with non-negligible population can be quite large for room-temperature simulations, even for relatively simple and small molecules like fluorobenzene. With bigger systems, which will typically have a larger number of low-energy modes, the problem can rapidly become intractable, forcing some concessions in the set of states to include. This brings us to the second aspect; the contributions of each hot band, and thus the overall temperature effects, can be strongly modulated by the resonance conditions. With small damping factors, the changes to the spectral band-shapes introduced with the temperature can be very large and poorly predicted by simulations considering only the vibrational ground state as starting point. Larger values tend to dampen the response overall, and these effects appear significantly attenuated. Since typical values of γ are in the order of hundreds of wavenumbers, the impact of temperature could be mild in practice. However, a more extensive study considering a broader panel of molecular systems and different preresonance conditions would be necessary to draw any further conclusion. The same generator used for fundamentals can enumerate and evaluate directly any group of thermally populated initial states without requiring a separate analytic derivation for each case. The impact and effect of temperature can this way be systematically studied.

### 4.4 RROA Spectrum of (*S*)-(+)-naproxen

As a final application, let us examine the RROA spectrum of (*S*)-(+)-naproxen. Naproxen and its derivatives are important chiral benchmark molecules, whose ROA spectra in resonance were among the first to be recorded.^45^ From a purely theoretical perspective, naproxen is also an interesting subject. The first two excited electronic states are relatively close in energies (see Table 2 and Figure S9 in the Supporting Information), which gives us the possibility to study the modulation of the RROA spectrum considering different resonance conditions involving these two states. Another noteworthy aspect to consider is their low oscillator strengths. While such conditions make experiments unfavorable due to the low expected yield, this means for our computations that the Herzberg–Teller contributions are likely to be of magnitude at least comparable to the Franck–Condon terms. In concrete terms, the predicted spectra may not be monosignate even when considering a single electronic state.^46^

**Table 2:** Vertical Excitation Energies and Oscillator Strengths (*f*) to the First Singlet Excited Electronic States of Naproxen at the CAM-B3LYP/def2-TZVP Level

State	Energy (eV)	Wavelength (nm)	Wavenumber (cm^–1^)	*f*
*S*_1_	4.2481	291.86	34 263	0.0572
*S*_2_	4.6903	264.34	37 830	0.0164
*S*_3_	5.7221	216.68	46 151	1.1566
*S*_4_	5.8810	210.82	47 434	0.0158
*S*_5_	5.9599	208.03	48 070	0.4062

Considering the zero-point vibrational energy differences of the two lowest excited states with respect to the ground state, which is extrapolated in the vertical Hessian model employed here, respectively at 30865.00 cm^–1^ (324 nm) and 35728.73 cm^–1^ (280 nm), three resonance conditions were chosen, with the incident frequencies tuned in resonance with *S*_1_ (30865.10 cm^–1^), *S*_2_ (35728.73 cm^–1^), and finally set halfway inbetween, which will be referred to in the following as midpoint condition (33296.87 cm^–1^). To build the full RROA response, we first constructed all relevant tensors for each intermediate electronic state (*S*_1_ and *S*_2_), and then summed them to generate the final invariants. The relative Duschinsky matrices and shift vectors within the vertical Hessian model are depicted in Figures S10 and S11 in the Supporting Information.

The spectral band-shape obtained from fundamental transitions in the fingerprint region for all three incident frequencies are displayed in Figure 6, while the “single electronic state” spectra are reported in Figure S12 in the Supporting Information. In all three conditions, the strongest band is located at 1718.8 cm^–1^ and corresponds to the in-plane deformation of the naphthyl unit (mode 72, depicted in Figure S13 in the Supporting Information). Its intensity is 9.29 × 10^–7^ cm^3^ mol^–1^ sr^–1^ at the *S*_1_ resonance, 1.57 × 10^–6^ at the midpoint, and 3.51 × 10^–6^ at the *S*_2_ resonance. Over the relatively narrow range chosen for the incident frequency, the mode is relatively unaffected, and sufficiently robust to detuning. By contrast, several low-intensity transitions are highly sensitive to the actual state in resonance. The 193.9 cm^–1^ feature changes from 1.28 × 10^–7^ cm^3^ mol^–1^ sr^–1^ at *S*_1_ to −6.52 × 10^–8^ at the midpoint and −6.99 × 10^–9^ at *S*_2_, while the nearby 185.0 cm^–1^ line evolves from −4.78 × 10^–8^ to 6.04 × 10^–8^ and then to 6.73 × 10^–8^. Looking at the single-state spectra (Figure S12 in the Supporting Information), we see that these mode-selective sign inversions arise from the different Herzberg–Teller contributions of the two intermediate states and from their coherent combination.

**Figure 6:**
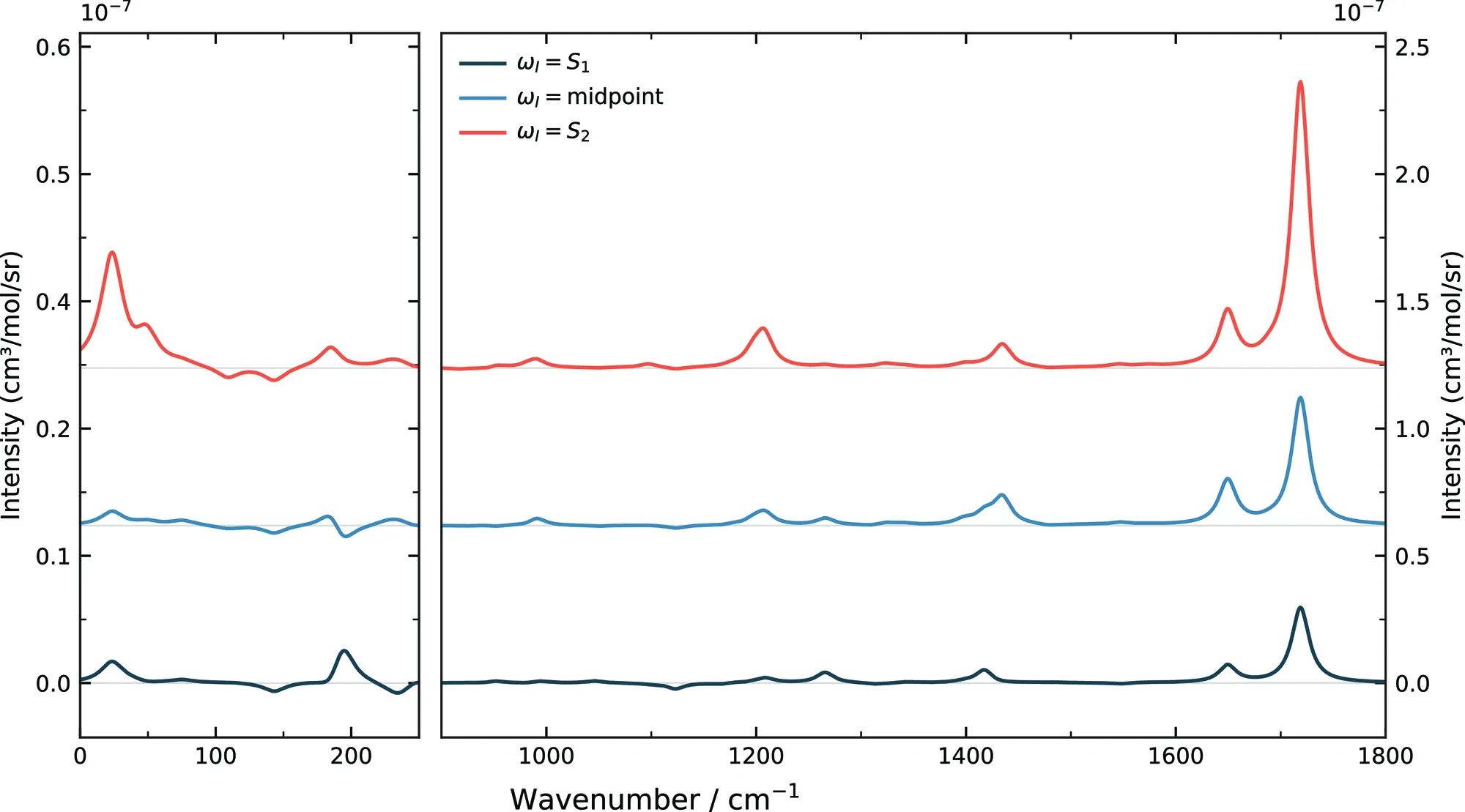
RROA spectrum of (*S*)-(+)-naproxen in the fingerprint region with contributions from the *S*_1_ and *S*_2_ intermediate states, evaluated at three different incident frequencies (*S*_1_ resonance, midpoint, and *S*_2_ resonance). Only fundamental transitions are included. Broadening was done by means of Lorentzian distribution functions with HWHM = 10 cm^–1^. Spectra are vertically offset for clarity. Black, blue, and orange lines correspond to incident frequencies tuned to the *S*_1_ resonance, the midpoint between the two states, and the *S*_2_ resonance, respectively.

Because the RROA signal is far weaker than the RR one, the contribution of nonfundamental transitions can become comparably more visible in measured band shapes. For this reason, transitions to first overtones and “1 + 1” combinations were also computed. The strongest overtone reaches 8.70 × 10^–8^ cm^3^ mol^–1^ sr^–1^ at 3437.7 cm^–1^ (first overtone of mode 72), whereas the strongest combination band reaches 1.43 × 10^–7^ at 3368.2 cm^–1^ (70 + 72).

As expected, the fingerprint region (Figure 7, full-scale spectrum in Figure S14 in the Supporting Information) is dominated by fundamental bands, with only a few low-intensity combinations giving rise to small shoulders of the two main bands in the 1600–2000 cm^–1^ range and some weak bands between 1800 and 2000 cm^–1^, of height similar to the C=O stretching band at 1842 cm^–1^. The results in the X-H stretching region (Figure 8) are far more intriguing. In the chosen resonance conditions, the intensities of the CH and OH stretching fundamentals are negligible, so only overtones and combination bands are visible. However, the region remains of modest intensity, with the highest peak about 1 order of magnitude lower than that of the fingerprint region. Not so surprisingly considering the mode mixing associated with the *S*_0_ → *S*_1_ and *S*_0_ → *S*_2_ transitions and their outsize number compared to overtones and fundamentals, combination bands are dominant. As a matter of fact, out of all computed transitions, the highest peak is comparable to overtones in terms of intensities, and the total integrated absolute intensity reaches 1.49 × 10^–6^ cm^3^ mol^–1^ sr^–1^, roughly 40% of that of the fundamentals (3.94 × 10^–6^), while the overall contribution of the overtone manifold is an order of magnitude weaker (1.76 × 10^–7^).

**Figure 7:**
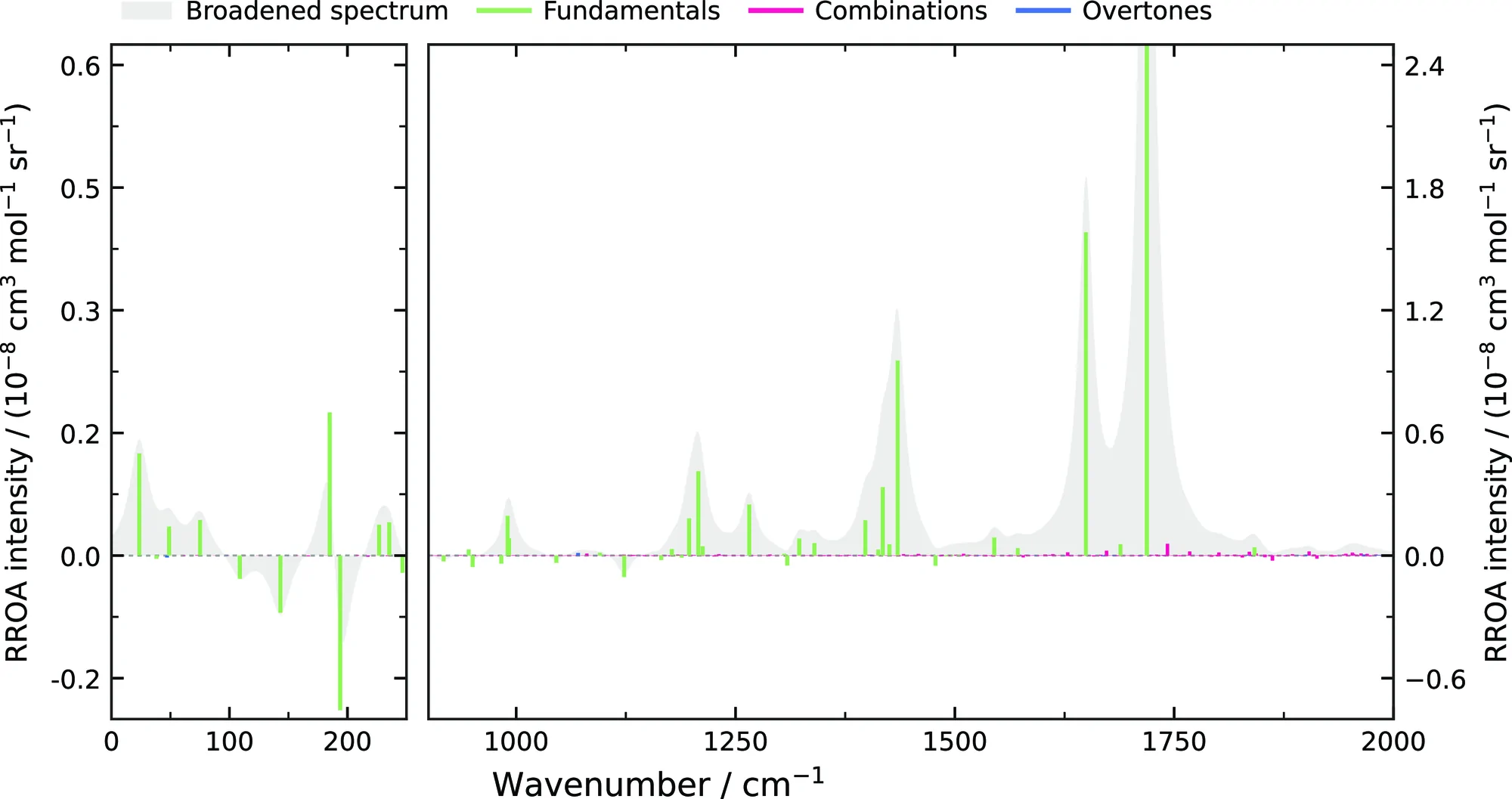
Fingerprint region (0–2000 cm^–1^) of the RROA spectrum of (*S*)-(+)-naproxen including two intermediate electronic states, *S*_1_ and *S*_2_, with the incident energy set at midpoint resonance. A damping factor of 500 cm^–1^ was used and Lorentzian functions with HWHMs = 10 cm^–1^ applied to broaden the stick spectrum. The strongest peak was clipped to highlight lower-intensity bands. The stick spectrum was arbitrarily scaled to fit the band-shape.

**Figure 8:**
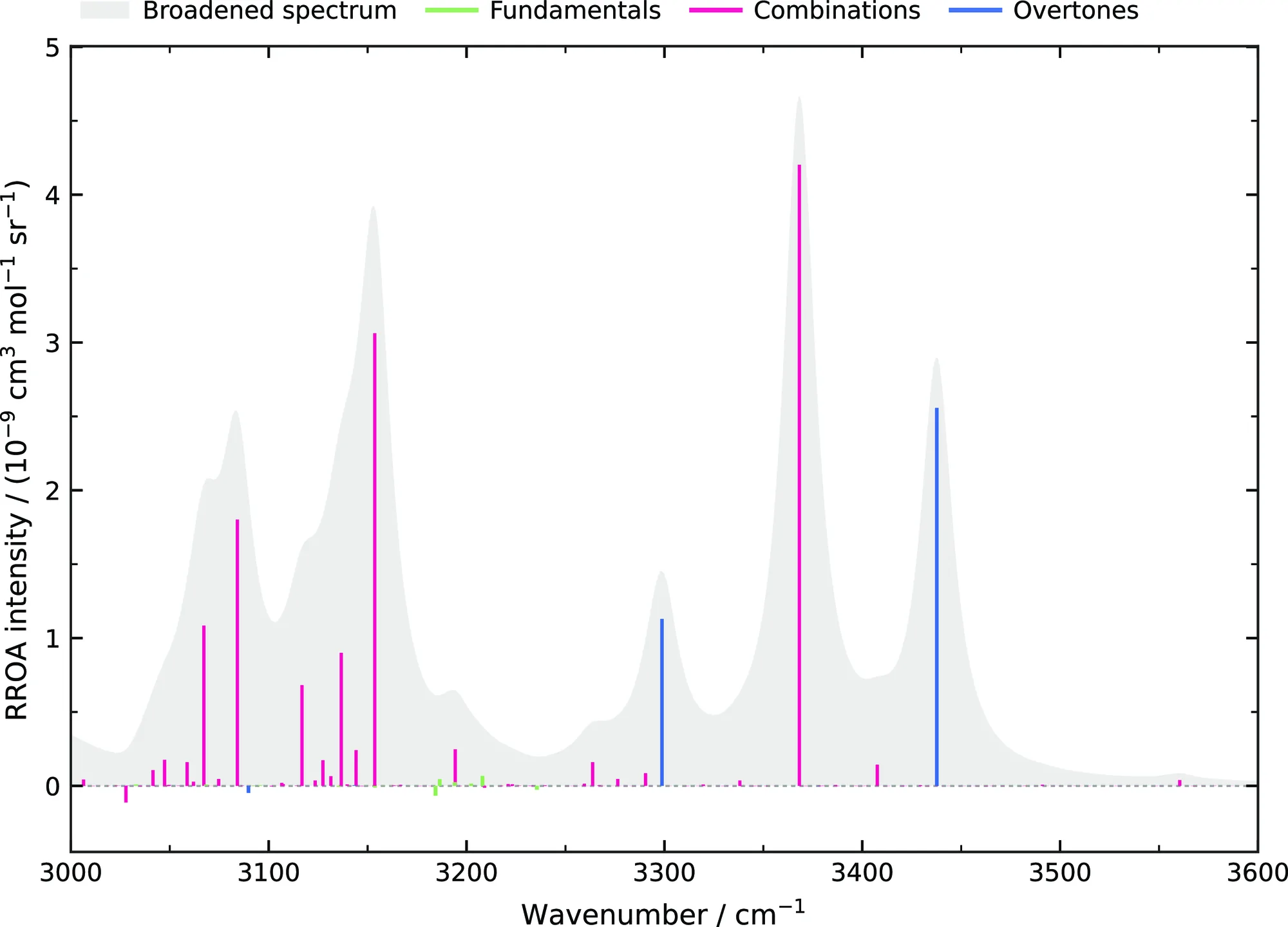
CH-stretching region (3000–3600 cm^–1^) of the RROA spectrum of (*S*)-(+)-naproxen including two intermediate electronic states, *S*_1_ and *S*_2_, with the incident energy set at midpoint resonance. A damping factor of 500 cm^–1^ was used and Lorentzian functions with HWHMs = 10 cm^–1^ applied to broaden the stick spectrum. The stick spectrum was arbitrarily scaled to fit the band-shape.

The intermediate range between the fingerprint and CH-stretching regions (Figure 9) is logically populated by nonfundamental bands, primarily combinations. Their intensity is low, about half of those in the X-H stretching region, but a rich structure is observed. It should be noted that anharmonicity is not accounted for in these calculations, the intensity arising from the mixing with the intermediate electronic state.

**Figure 9:**
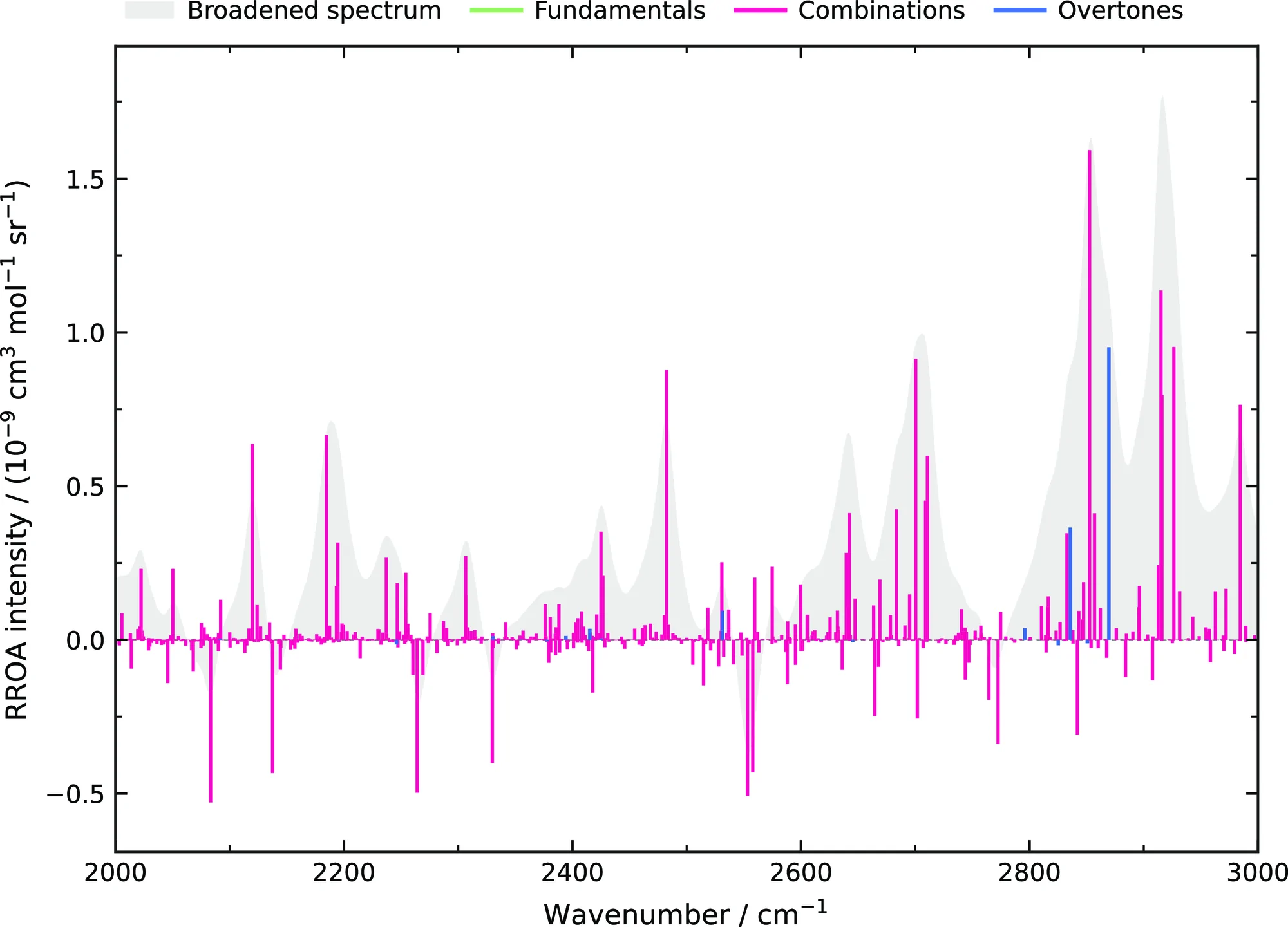
Nonfundamental mid-IR region (2000–3000 cm^–1^) of the RROA spectrum of (*S*)-(+)-naproxen including two intermediate electronic states, *S*_1_ and *S*_2_, with the incident energy set at midpoint resonance. A damping factor of 500 cm^–1^ was used and Lorentzian functions with HWHMs = 10 cm^–1^ applied to broaden the stick spectrum. The stick spectrum was arbitrarily scaled to fit the band-shape.

Taken together, these results show that the same symbolic framework can be easily transferred to RROA observables, including coherent sums over close electronic states, and higher-order manifolds beyond the fundamental regime without the need for transition-specific rederivation.

## 5 Conclusions

We have introduced an automated path-integral framework for simulating resonance Raman spectra and treating general vibrational transitions within a single symbolic workflow. The central idea is to construct the RR tensor invariants directly from low-rank Gaussian-moment intermediates, rather than explicitly generating high-rank correlation tensors and contracting them only afterward. The resulting symbolic addends are canonicalized, simplified, and evaluated through optimized contraction paths, reducing the cost of several higher-order transition classes relative to explicit high-rank formulations.

Scaling tests show that all transition classes share a common dense linear-algebra baseline, formally cubic in the number of normal modes and with a small prefactor. Beyond this baseline, the cost depends on the structure and size of the generated expressions: for simple progressions it remains modest, whereas sufficiently dense hot-band manifolds become limited by the rapid growth in the number of transitions. Overall, avoiding explicit high-rank intermediates makes exact evaluation practical across a broad range of higher-order transitions.

Fluorobenzene provides a proof of principle for Boltzmann-weighted initial-state ensembles, while (*S*)-(+)-naproxen shows that the same machinery transfers naturally to RROA observables and coherent multistate calculations.

## Supplementary Material



## Data Availability

The ARIES Python package used for the RR and RROA calculations is freely available at doi.org/10.5281/zenodo.20312218. Input files, processed data, and plotting scripts to reproduce all figures and tables in this work are archived at doi.org/10.5281/zenodo.20313513.

## A: Phase Factorization of the Prefactor

From the definition of *R*(*t*) from eq 30

35
$$R\left(t\right)=\frac{1}{\sqrt{\det\left(I-\mathrm{WVWV}\right)}}$$

where $W=(A+\overset{®}{\mathrm{\Omega ̅}}{)}^{-1}\left(A-\overset{®}{\mathrm{\Omega ̅}}\right)$ and ***A*** = ***J*** ^*T*^ **Ω** ***J***. Defining

36
$$B={\overset{®}{\mathrm{\Omega ̅}}}^{-1/2}A{\overset{®}{\mathrm{\Omega ̅}}}^{-1/2}$$

one obtains

37
$$W={\overset{®}{\mathrm{\Omega ̅}}}^{-1/2}S{\overset{®}{\mathrm{\Omega ̅}}}^{1/2},,S={\left(B+I\right)}^{-1}\left(B-I\right)$$

Matrix ***S*** is symmetric. If *b*_*j*_ > 0 are the eigenvalues of ***B***, the eigenvalues of ***S*** are

38
$$s_{j}=\frac{b_{j}-1}{b_{j}+1}$$

and therefore |*s*_*j*_|< 1 for all *j*.

The product ***WV*** inherits this bound through the same similarity transformation, since ***V*** commutes with $\overset{®}{\mathrm{\Omega ̅}}$

39
$${\overset{®}{\mathrm{\Omega ̅}}}^{1/2}\mathrm{WV}{\overset{®}{\mathrm{\Omega ̅}}}^{-1/2}=\mathrm{SV}$$

Thus, ***WV*** and ***SV*** have the same eigenvalues. Since ***S*** is symmetric, ∥ ***S*** ∥_2_ = max_*j*_ |*s*_*j*_ |< 1, while ***V*** is unitary and ∥ ***V*** ∥_2_ = 1. Therefore, ∥ ***SV*** ∥_2_ ≤ ∥ ***S*** ∥_2_ ∥ ***V*** ∥_2_ < 1 so all eigenvalues λ_*j*_(*t*) of ***SV*** satisfy

40
$$\left|\lambda_{j}\left(t\right)\right|<1$$

The residual factor can then be written as

41
$$R\left(t\right)=\prod_{j}{\left[1-\lambda_{j}{\left(t\right)}^{2}\right]}^{-1/2}$$

Since |λ_*j*_(*t*)|< 1, each factor 1−λ_*j*_(*t*)^2^ lies in the open right half-plane. It therefore cannot vanish or lie on the negative real axis, so its principal square root is defined unambiguously.

This factor-wise construction is essential. Although each individual factor has a well-defined principal square root, their product

42
$$\prod_{j}\left[1-\lambda_{j}{\left(t\right)}^{2}\right]=\det\left(I-\mathrm{WVWV}\right)$$

may still cross the negative real axis. Therefore, evaluating *R*(*t*) with a single principal square root of the full determinant can introduce discontinuous sign changes. Taking the principal square root factor by factor fixes the branch before forming the product.

## Notes

Anthracene vertical excited states and Duschinsky parameters, class-resolved TD/TI comparison spectra and transition tables for anthracene and anisole, fluorobenzene vibrational frequencies, Duschinsky parameters and hot-band analyses, and state-resolved naproxen spectra with Duschinsky parameters and normal-mode depictions (PDF)
