# Supplementary material for: Efficient Simulation of Higher-Order Transitions in Resonance Raman Spectroscopy and Its Chiral Extension
Source: J Chem Theory Comput. 2026 Aug 29;22(18):9592–607. doi: 10.1021/acs.jctc.6c01051 (PMC13602930; doi:10.1021/acs.jctc.6c01051)
Supplement: Supplementary file 1 [file ct6c01051_si_001.pdf]

Supporting Information: Efficient Simulation of Higher-Order Transitions in Resonance Raman Spectroscopy and its Chiral Extension

Andrea Bianchi* and Julien Bloino*

Scuola Normale Superiore, Piazza dei Cavalieri 7, 56126 Pisa, Italy

E-mail: andrea.bianchi@sns.it; julien.bloino@sns.it

1 Additional data for anthracene

1.1 Singlet electronic states

The first five vertical singlet excited states of anthracene at the ωB97X/def2-TZVP level are summarized in Table S1. The dominant orbital contributions are reported with the corresponding Gaussian amplitudes. The RR calculations discussed in the main text use the fourth excited state (S_4).

Table S1: First five vertical singlet excited states of anthracene.

State	Energy (eV)	Wavelength (nm)	f	Dominant configurations
S_1	3.8016	326.13	0.0893	46 → 49 (0.12892)<br>47 → 48 (0.69259)
S_2	4.1829	296.41	0.0007	46 → 48 (0.49905)<br>47 → 49 (-0.48279)
S_3	5.3862	230.19	0.0000	45 → 48 (0.49234)<br>47 → 50 (0.49739)
S_4	5.5873	221.91	2.1520	46 → 48 (0.48532)<br>47 → 49 (0.50337)
S_5	5.9667	207.79	0.0000	44 → 50 (-0.11333)<br>45 → 48 (0.48583)<br>45 → 51 (0.14535)<br>47 → 50 (-0.47610)

1.2 Duschinsky transformation for $S_0 \rightarrow S_4$

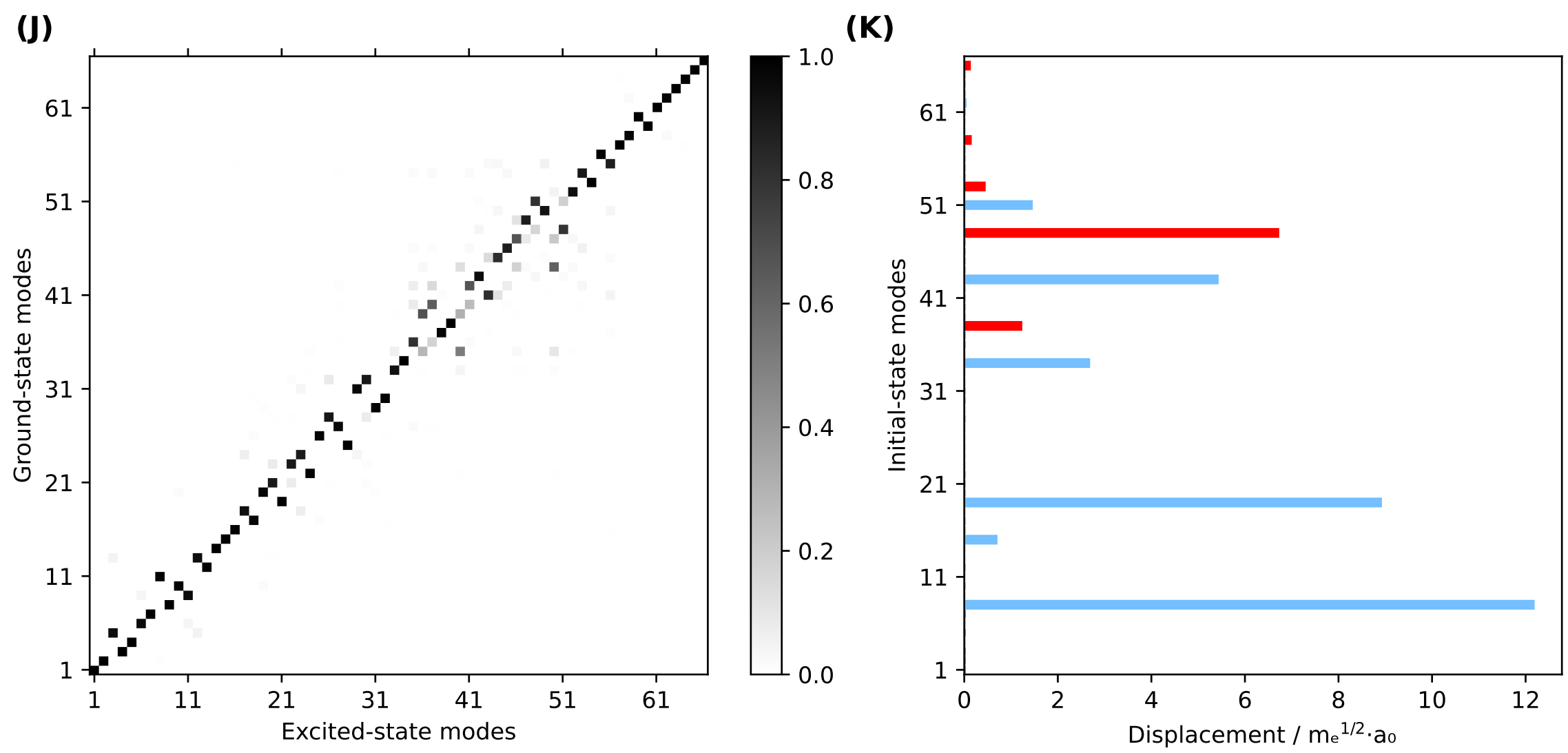

Figure S1: Duschinsky matrix (**J**, left panel) and shift vector (**K**, right panel) associated to the $S_0 \rightarrow S_4$ transition of anthracene within the vertical Hessian model. Each matrix element has been squared and a shade of gray assigned based on the value (1: black, 0: white). The shift vector is reported in atomic units. Red bars indicate negative values, blue for positive ones.

1.3 Class-specific resonance Raman spectra

The anthracene TD/TI validation in the main text combines fundamentals, overtones, and combination bands into a single total RR band-shape. For clarity, the three ground-state transition classes are shown separately here using the same Gaussian broadening settings as in the main-text comparison.

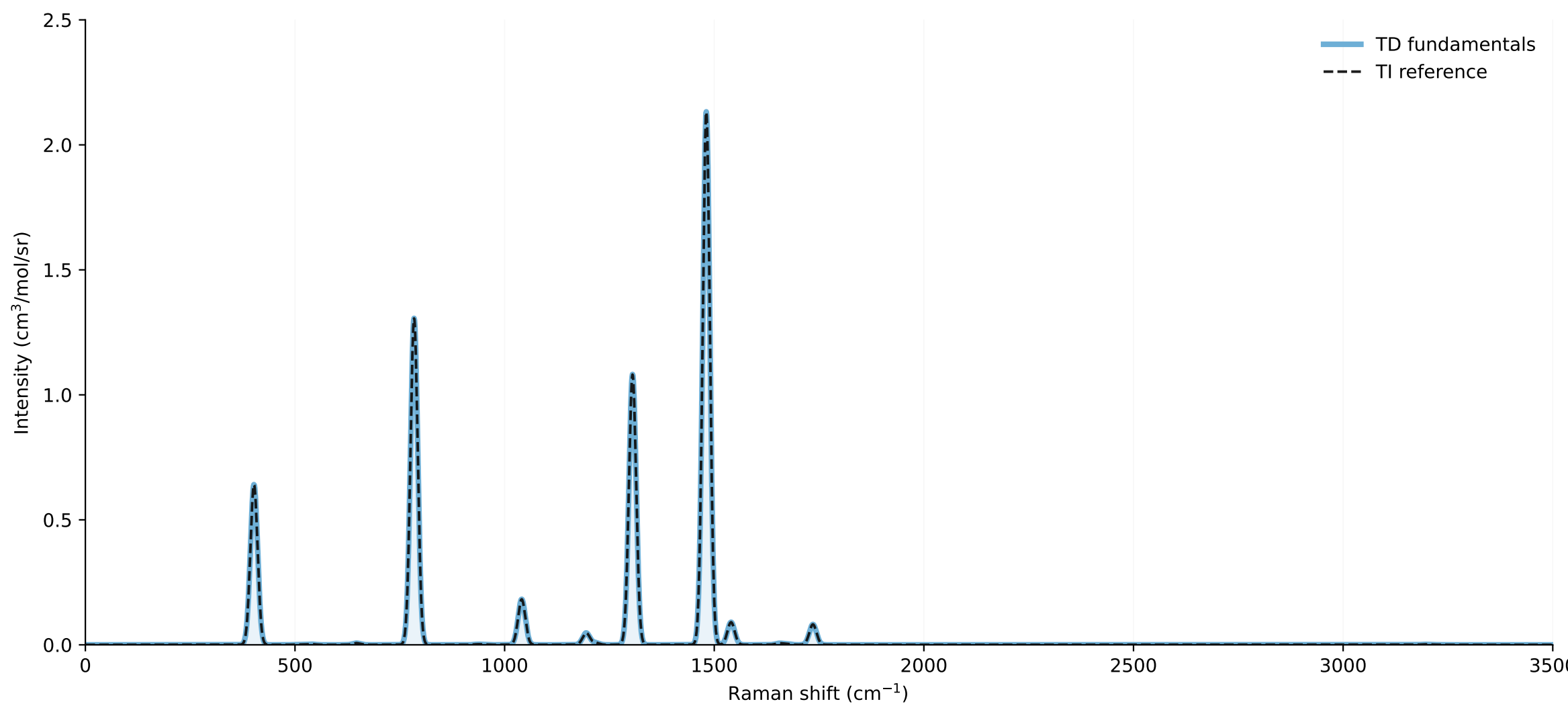

Figure S2: Anthracene TD/TI comparison for the fundamental manifold ($0 \rightarrow 1_i$). Solid and dashed curves denote the TD and TI spectra, respectively.

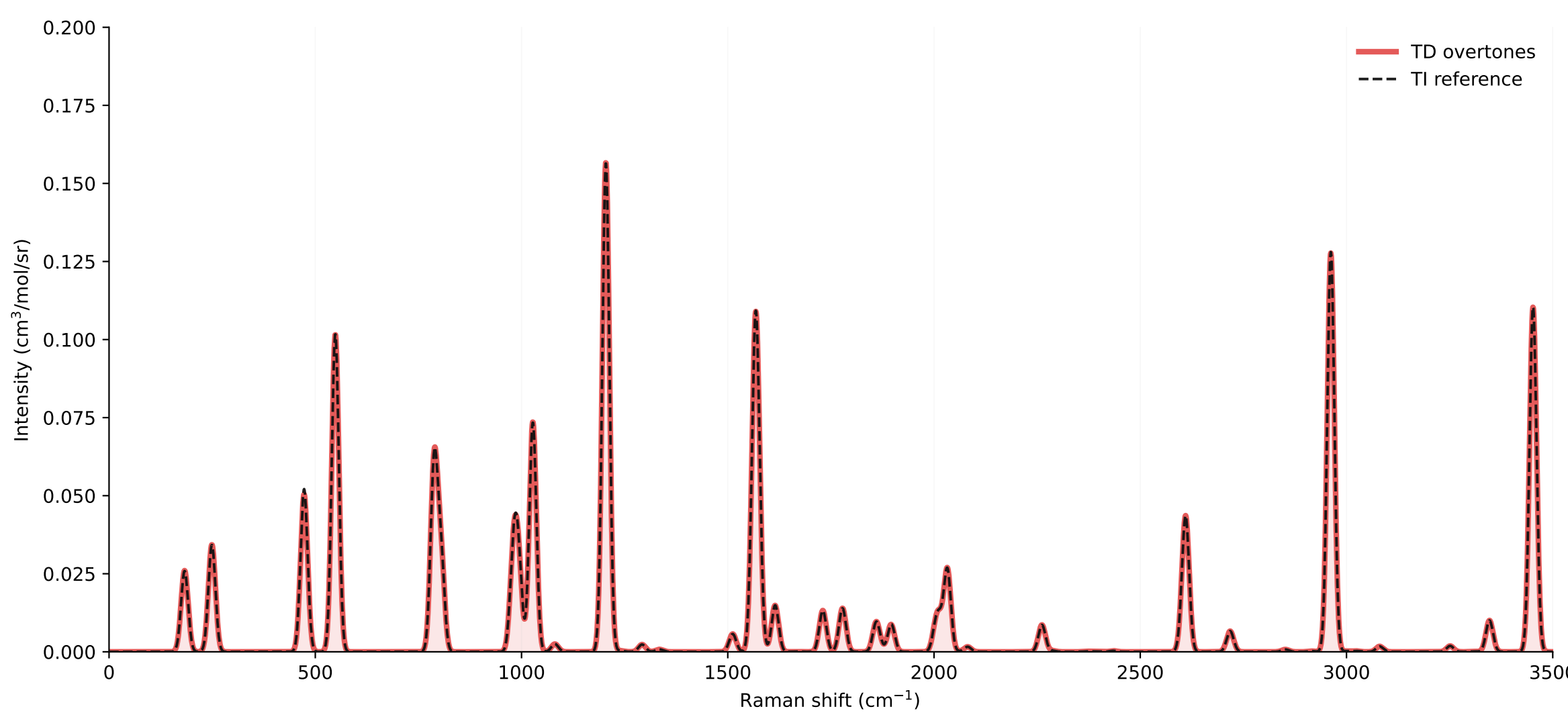

Figure S3: Anthracene TD/TI comparison for the overtone manifold ($0 \rightarrow 2_i$). Solid and dashed curves denote the TD and TI spectra, respectively.

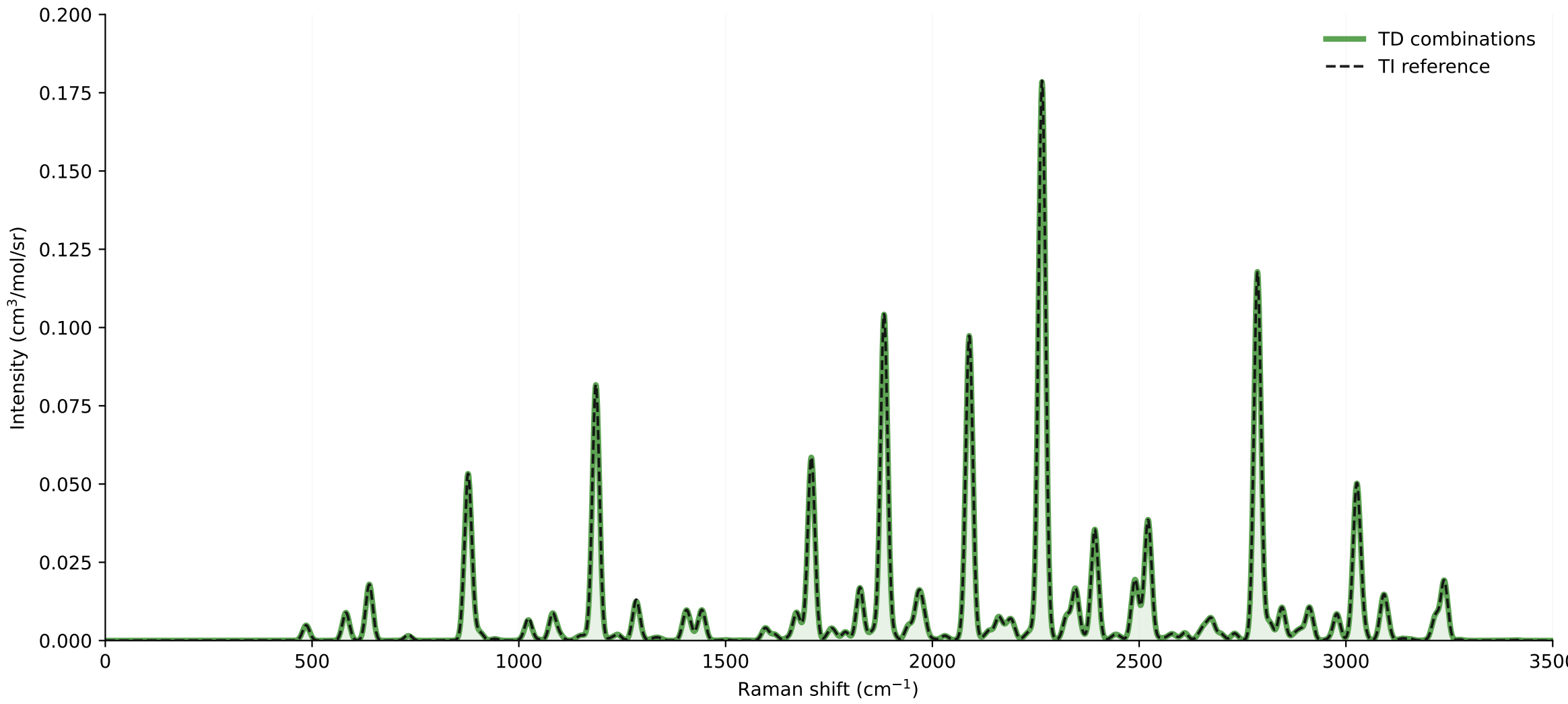

Figure S4: Anthracene TD/TI comparison for the combination-band manifold ($0 \rightarrow 1_i 1_j$). Solid and dashed curves denote the TD and TI spectra, respectively.

1.4 Ground-state transition information

The anthracene TD/TI RR validation data for ground-state transitions are reported in Table S2 and grouped by transition class. The table reports transitions up to 4000 cm^{-1} with $I^{TD} > 10^{-4}$ $cm^3\,mol^{-1}\,sr^{-1}$. The included classes comprise fundamentals ($0 \rightarrow 1_i$), overtones ($0 \rightarrow 2_i$), and combination bands ($0 \rightarrow 1_i 1_j$). For each transition, the table reports the TD frequency, the TD and default-prescreening TI intensities, and the relative difference $\Delta = \Delta I/I = \left| I^{TD} - I^{TI} \right| / I^{TI}$.

Table S2: Anthracene TD/TI RR validation data for ground-state transitions grouped by transition class. The table reports transitions up to 4000 cm^{-1} with $I^{TD} > 10^{-4}$ $cm^3\,mol^{-1}\,sr^{-1}$. Intensities are reported in units of 10^{-1} $cm^3\,mol^{-1}\,sr^{-1}$; relative differences are reported in percent.

Transition	ν	I^{TD}	I^{TI}	Δ
		$0 \rightarrow 1_i$		
$\lvert 0\rangle \rightarrow \lvert 1_3\rangle$	236.38	0.002	0.002	0.04
$\lvert 0\rangle \rightarrow \lvert 1_7\rangle$	401.51	0.427	0.427	0.12
$\lvert 0\rangle \rightarrow \lvert 1_8\rangle$	402.22	135.959	136.010	0.04
$\lvert 0\rangle \rightarrow \lvert 1_{10}\rangle$	496.29	0.070	0.070	0.03
$\lvert 0\rangle \rightarrow \lvert 1_{12}\rangle$	540.45	0.567	0.566	0.19
$\lvert 0\rangle \rightarrow \lvert 1_{15}\rangle$	646.09	1.469	1.467	0.19
$\lvert 0\rangle \rightarrow \lvert 1_{19}\rangle$	784.12	277.867	277.980	0.04
$\lvert 0\rangle \rightarrow \lvert 1_{20}\rangle$	787.38	0.040	0.040	0.01
$\lvert 0\rangle \rightarrow \lvert 1_{27}\rangle$	936.79	0.520	0.518	0.20
$\lvert 0\rangle \rightarrow \lvert 1_{29}\rangle$	1002.35	0.079	0.079	0.07
$\lvert 0\rangle \rightarrow \lvert 1_{34}\rangle$	1040.73	38.829	38.763	0.17
$\lvert 0\rangle \rightarrow \lvert 1_{36}\rangle$	1143.44	0.005	0.005	0.16
$\lvert 0\rangle \rightarrow \lvert 1_{38}\rangle$	1194.50	10.147	10.152	0.05
$\lvert 0\rangle \rightarrow \lvert 1_{40}\rangle$	1217.25	1.707	1.704	0.16
$\lvert 0\rangle \rightarrow \lvert 1_{42}\rangle$	1304.62	0.229	0.228	0.18
$\lvert 0\rangle \rightarrow \lvert 1_{43}\rangle$	1305.16	229.935	229.980	0.02
$\lvert 0\rangle \rightarrow \lvert 1_{46}\rangle$	1425.78	0.006	0.006	0.07
$\lvert 0\rangle \rightarrow \lvert 1_{48}\rangle$	1481.01	453.918	453.680	0.05
$\lvert 0\rangle \rightarrow \lvert 1_{51}\rangle$	1540.09	19.257	19.261	0.02
$\lvert 0\rangle \rightarrow \lvert 1_{53}\rangle$	1654.57	1.262	1.264	0.14
$\lvert 0\rangle \rightarrow \lvert 1_{54}\rangle$	1673.33	0.968	0.968	0.10

Continued on next page

Table S2: Anthracene TD/TI RR validation data for ground-state transitions grouped by transition class. The table reports transitions up to 4000 cm^{-1} with $I^{TD} > 10^{-4}$ $cm^3\,mol^{-1}\,sr^{-1}$. Intensities are reported in units of 10^{-1} $cm^3\,mol^{-1}\,sr^{-1}$; relative differences are reported in percent. (continued)

Transition	ν	I^{TD}	I^{TI}	Δ
$\|0\rangle \to \|1_{56}\rangle$	1735.30	17.402	17.390	0.07
$\|0\rangle \to \|1_{58}\rangle$	3193.70	0.392	0.392	0.08
$\|0\rangle \to \|1_{59}\rangle$	3198.45	0.040	0.040	0.00
$\|0\rangle \to \|1_{62}\rangle$	3203.20	0.004	0.004	0.09
$\|0\rangle \to \|1_{63}\rangle$	3217.75	0.035	0.035	0.00
$\|0\rangle \to \|1_{66}\rangle$	3230.43	0.127	0.127	0.08
	$0 \to 2_i$			
$\|0\rangle \to \|2_{1}\rangle$	183.17	5.537	5.512	0.46
$\|0\rangle \to \|2_{2}\rangle$	249.36	7.321	7.290	0.43
$\|0\rangle \to \|2_{3}\rangle$	472.76	10.923	10.911	0.11
$\|0\rangle \to \|2_{4}\rangle$	477.16	0.215	0.214	0.25
$\|0\rangle \to \|2_{5}\rangle$	548.53	21.700	21.675	0.11
$\|0\rangle \to \|2_{6}\rangle$	788.04	12.708	12.686	0.17
$\|0\rangle \to \|2_{7}\rangle$	803.01	0.713	0.712	0.07
$\|0\rangle \to \|2_{8}\rangle$	804.45	5.803	5.804	0.01
$\|0\rangle \to \|2_{9}\rangle$	980.63	6.404	6.406	0.02
$\|0\rangle \to \|2_{10}\rangle$	992.58	5.722	5.726	0.07
$\|0\rangle \to \|2_{11}\rangle$	1027.46	15.642	15.648	0.04
$\|0\rangle \to \|2_{12}\rangle$	1080.90	0.535	0.534	0.17
$\|0\rangle \to \|2_{13}\rangle$	1204.48	33.351	33.348	0.01
$\|0\rangle \to \|2_{14}\rangle$	1239.74	0.078	0.078	0.13
$\|0\rangle \to \|2_{15}\rangle$	1292.18	0.500	0.500	0.06
$\|0\rangle \to \|2_{16}\rangle$	1333.84	0.172	0.172	0.04
$\|0\rangle \to \|2_{17}\rangle$	1511.87	1.220	1.218	0.16
$\|0\rangle \to \|2_{18}\rangle$	1562.85	5.562	5.560	0.04
$\|0\rangle \to \|2_{19}\rangle$	1568.25	15.698	15.705	0.05
$\|0\rangle \to \|2_{20}\rangle$	1574.77	4.034	4.029	0.12
$\|0\rangle \to \|2_{21}\rangle$	1614.86	3.176	3.172	0.13
$\|0\rangle \to \|2_{22}\rangle$	1666.16	0.024	0.024	0.62
$\|0\rangle \to \|2_{23}\rangle$	1730.38	2.816	2.817	0.04
$\|0\rangle \to \|2_{24}\rangle$	1778.16	2.969	2.971	0.08
$\|0\rangle \to \|2_{25}\rangle$	1855.36	0.040	0.040	0.17
$\|0\rangle \to \|2_{26}\rangle$	1860.28	1.998	1.998	0.03

Continued on next page

Table S2: Anthracene TD/TI RR validation data for ground-state transitions grouped by transition class. The table reports transitions up to 4000 cm^{-1} with $I^{TD} > 10^{-4}$ $cm^3\,mol^{-1}\,sr^{-1}$. Intensities are reported in units of 10^{-1} $cm^3\,mol^{-1}\,sr^{-1}$; relative differences are reported in percent. (continued)

Transition	ν	I^{TD}	I^{TI}	Δ
$\|0\rangle \to \|2_{27}\rangle$	1873.58	0.124	0.123	0.50
$\|0\rangle \to \|2_{28}\rangle$	1896.41	1.884	1.883	0.03
$\|0\rangle \to \|2_{29}\rangle$	2004.70	1.601	1.599	0.12
$\|0\rangle \to \|2_{30}\rangle$	2014.22	1.532	1.531	0.08
$\|0\rangle \to \|2_{31}\rangle$	2031.76	2.807	2.803	0.13
$\|0\rangle \to \|2_{32}\rangle$	2033.00	2.780	2.777	0.12
$\|0\rangle \to \|2_{33}\rangle$	2059.53	0.028	0.028	0.44
$\|0\rangle \to \|2_{34}\rangle$	2081.47	0.347	0.346	0.44
$\|0\rangle \to \|2_{35}\rangle$	2261.59	1.837	1.837	0.01
$\|0\rangle \to \|2_{36}\rangle$	2286.88	0.073	0.072	0.36
$\|0\rangle \to \|2_{37}\rangle$	2371.36	0.034	0.034	0.14
$\|0\rangle \to \|2_{38}\rangle$	2389.00	0.014	0.014	0.37
$\|0\rangle \to \|2_{39}\rangle$	2394.34	0.006	0.006	0.71
$\|0\rangle \to \|2_{40}\rangle$	2434.49	0.072	0.072	0.47
$\|0\rangle \to \|2_{41}\rangle$	2602.12	0.676	0.677	0.18
$\|0\rangle \to \|2_{42}\rangle$	2609.24	0.575	0.577	0.27
$\|0\rangle \to \|2_{43}\rangle$	2610.32	8.270	8.275	0.05
$\|0\rangle \to \|2_{44}\rangle$	2717.83	1.410	1.411	0.11
$\|0\rangle \to \|2_{45}\rangle$	2728.48	0.007	0.007	1.15
$\|0\rangle \to \|2_{46}\rangle$	2851.56	0.162	0.162	0.06
$\|0\rangle \to \|2_{47}\rangle$	2916.55	0.050	0.049	0.78
$\|0\rangle \to \|2_{48}\rangle$	2962.02	27.189	27.254	0.24
$\|0\rangle \to \|2_{49}\rangle$	3000.33	0.050	0.051	0.47
$\|0\rangle \to \|2_{50}\rangle$	3023.33	0.060	0.060	0.11
$\|0\rangle \to \|2_{51}\rangle$	3080.17	0.376	0.376	0.04
$\|0\rangle \to \|2_{52}\rangle$	3251.18	0.404	0.404	0.06
$\|0\rangle \to \|2_{53}\rangle$	3309.14	0.026	0.026	0.42
$\|0\rangle \to \|2_{54}\rangle$	3346.67	2.141	2.143	0.07
$\|0\rangle \to \|2_{55}\rangle$	3452.26	23.503	23.515	0.05
$\|0\rangle \to \|2_{56}\rangle$	3470.60	0.027	0.027	0.37
		$0 \to 1_i 1_j$		
$\|0\rangle \to \|1_6\,1_1\rangle$	485.60	1.047	1.043	0.37
$\|0\rangle \to \|1_5\,1_3\rangle$	510.64	0.001	0.001	1.12

Continued on next page

Table S2: Anthracene TD/TI RR validation data for ground-state transitions grouped by transition class. The table reports transitions up to 4000 cm^{-1} with $I^{TD} > 10^{-4}$ $cm^3\,mol^{-1}\,sr^{-1}$. Intensities are reported in units of 10^{-1} $cm^3\,mol^{-1}\,sr^{-1}$; relative differences are reported in percent. (continued)

Transition	ν	I^{TD}	I^{TI}	Δ
$\|0\rangle \to \|1_9\,1_1\rangle$	581.90	1.902	1.898	0.23
$\|0\rangle \to \|1_{11}\,1_2\rangle$	638.41	3.833	3.818	0.39
$\|0\rangle \to \|1_{10}\,1_3\rangle$	732.67	0.407	0.405	0.53
$\|0\rangle \to \|1_8\,1_7\rangle$	803.73	0.012	0.012	0.51
$\|0\rangle \to \|1_{17}\,1_1\rangle$	847.52	0.016	0.016	0.06
$\|0\rangle \to \|1_{13}\,1_5\rangle$	876.50	10.277	10.233	0.43
$\|0\rangle \to \|1_9\,1_6\rangle$	884.33	1.643	1.638	0.27
$\|0\rangle \to \|1_{10}\,1_8\rangle$	898.52	0.002	0.002	0.07
$\|0\rangle \to \|1_{16}\,1_4\rangle$	905.50	0.005	0.005	0.80
$\|0\rangle \to \|1_{18}\,1_2\rangle$	906.10	0.523	0.523	0.13
$\|0\rangle \to \|1_{12}\,1_7\rangle$	941.95	0.103	0.102	0.36
$\|0\rangle \to \|1_{12}\,1_8\rangle$	942.67	0.015	0.015	0.62
$\|0\rangle \to \|1_{24}\,1_2\rangle$	1013.76	0.144	0.144	0.31
$\|0\rangle \to \|1_{26}\,1_1\rangle$	1021.72	0.031	0.031	0.06
$\|0\rangle \to \|1_{20}\,1_3\rangle$	1023.76	1.421	1.420	0.11
$\|0\rangle \to \|1_{15}\,1_8\rangle$	1048.32	0.064	0.065	0.76
$\|0\rangle \to \|1_{21}\,1_5\rangle$	1081.69	1.824	1.821	0.16
$\|0\rangle \to \|1_{30}\,1_1\rangle$	1098.70	0.378	0.376	0.44
$\|0\rangle \to \|1_{23}\,1_5\rangle$	1139.46	0.075	0.074	0.25
$\|0\rangle \to \|1_{31}\,1_2\rangle$	1140.56	0.004	0.004	0.23
$\|0\rangle \to \|1_{32}\,1_2\rangle$	1141.18	0.001	0.001	0.22
$\|0\rangle \to \|1_{17}\,1_6\rangle$	1149.95	0.285	0.285	0.18
$\|0\rangle \to \|1_{25}\,1_4\rangle$	1166.26	0.199	0.199	0.08
$\|0\rangle \to \|1_{19}\,1_7\rangle$	1185.63	0.025	0.025	0.34
$\|0\rangle \to \|1_{19}\,1_8\rangle$	1186.35	17.349	17.380	0.18
$\|0\rangle \to \|1_{20}\,1_8\rangle$	1189.61	0.001	0.001	0.09
$\|0\rangle \to \|1_{28}\,1_5\rangle$	1222.47	0.140	0.140	0.24
$\|0\rangle \to \|1_{29}\,1_3\rangle$	1238.73	0.390	0.389	0.16
$\|0\rangle \to \|1_{17}\,1_9\rangle$	1246.25	0.026	0.026	0.08
$\|0\rangle \to \|1_{19}\,1_{10}\rangle$	1280.41	0.004	0.004	0.06
$\|0\rangle \to \|1_{20}\,1_{10}\rangle$	1283.68	2.673	2.673	0.01
$\|0\rangle \to \|1_{31}\,1_5\rangle$	1290.15	0.018	0.018	0.33
$\|0\rangle \to \|1_{32}\,1_5\rangle$	1290.76	0.073	0.073	0.32
$\|0\rangle \to \|1_{18}\,1_{11}\rangle$	1295.16	0.100	0.100	0.16

Continued on next page

Table S2: Anthracene TD/TI RR validation data for ground-state transitions grouped by transition class. The table reports transitions up to 4000 cm^{-1} with $I^{TD} > 10^{-4}$ $cm^3\,mol^{-1}\,sr^{-1}$. Intensities are reported in units of 10^{-1} $cm^3\,mol^{-1}\,sr^{-1}$; relative differences are reported in percent. (continued)

Transition	ν	I^{TD}	I^{TI}	Δ
$\|0\rangle \to \|1_{26}\,1_6\rangle$	1324.16	0.097	0.097	0.05
$\|0\rangle \to \|1_{19}\,1_{12}\rangle$	1324.57	0.032	0.033	0.42
$\|0\rangle \to \|1_{27}\,1_7\rangle$	1338.30	0.161	0.162	0.01
$\|0\rangle \to \|1_{27}\,1_8\rangle$	1339.02	0.014	0.014	0.27
$\|0\rangle \to \|1_{30}\,1_6\rangle$	1401.13	0.057	0.057	0.21
$\|0\rangle \to \|1_{24}\,1_{11}\rangle$	1402.81	1.233	1.233	0.00
$\|0\rangle \to \|1_{29}\,1_8\rangle$	1404.57	0.002	0.002	0.10
$\|0\rangle \to \|1_{21}\,1_{13}\rangle$	1409.67	0.968	0.967	0.07
$\|0\rangle \to \|1_{26}\,1_9\rangle$	1420.45	0.004	0.004	0.01
$\|0\rangle \to \|1_{37}\,1_4\rangle$	1424.26	0.003	0.003	0.20
$\|0\rangle \to \|1_{19}\,1_{15}\rangle$	1430.21	0.228	0.228	0.21
$\|0\rangle \to \|1_{34}\,1_7\rangle$	1442.24	0.003	0.003	0.33
$\|0\rangle \to \|1_{34}\,1_8\rangle$	1442.96	1.999	2.004	0.26
$\|0\rangle \to \|1_{22}\,1_{14}\rangle$	1452.95	0.006	0.005	1.36
$\|0\rangle \to \|1_{23}\,1_{13}\rangle$	1467.43	0.009	0.009	0.32
$\|0\rangle \to \|1_{27}\,1_{12}\rangle$	1477.24	0.002	0.002	1.06
$\|0\rangle \to \|1_{30}\,1_9\rangle$	1497.43	0.052	0.052	0.03
$\|0\rangle \to \|1_{29}\,1_{10}\rangle$	1498.64	0.005	0.005	0.42
$\|0\rangle \to \|1_{31}\,1_{11}\rangle$	1529.61	0.009	0.009	0.09
$\|0\rangle \to \|1_{32}\,1_{11}\rangle$	1530.23	0.002	0.002	0.10
$\|0\rangle \to \|1_{41}\,1_4\rangle$	1539.64	0.005	0.005	0.25
$\|0\rangle \to \|1_{36}\,1_7\rangle$	1544.94	0.018	0.018	0.17
$\|0\rangle \to \|1_{28}\,1_{13}\rangle$	1550.44	0.001	0.001	0.34
$\|0\rangle \to \|1_{20}\,1_{19}\rangle$	1571.51	0.002	0.002	0.06
$\|0\rangle \to \|1_{34}\,1_{12}\rangle$	1581.18	0.004	0.004	0.33
$\|0\rangle \to \|1_{25}\,1_{16}\rangle$	1594.60	0.305	0.306	0.28
$\|0\rangle \to \|1_{38}\,1_8\rangle$	1596.72	0.519	0.520	0.18
$\|0\rangle \to \|1_{45}\,1_4\rangle$	1602.82	0.048	0.048	0.22
$\|0\rangle \to \|1_{31}\,1_{13}\rangle$	1618.12	0.077	0.077	0.11
$\|0\rangle \to \|1_{32}\,1_{13}\rangle$	1618.74	0.282	0.282	0.11
$\|0\rangle \to \|1_{40}\,1_7\rangle$	1618.75	0.003	0.003	0.49
$\|0\rangle \to \|1_{40}\,1_8\rangle$	1619.47	0.046	0.046	0.12
$\|0\rangle \to \|1_{33}\,1_{14}\rangle$	1649.63	0.167	0.168	0.22
$\|0\rangle \to \|1_{24}\,1_{18}\rangle$	1670.50	1.469	1.467	0.15

Continued on next page

Table S2: Anthracene TD/TI RR validation data for ground-state transitions grouped by transition class. The table reports transitions up to 4000 cm^{-1} with $I^{TD} > 10^{-4}$ $cm^3\,mol^{-1}\,sr^{-1}$. Intensities are reported in units of 10^{-1} $cm^3\,mol^{-1}\,sr^{-1}$; relative differences are reported in percent. (continued)

Transition	ν	I^{TD}	I^{TI}	Δ
$\lvert 0\rangle \to \lvert 1_{23}\,1_{21}\rangle$	1672.62	0.378	0.377	0.24
$\lvert 0\rangle \to \lvert 1_{26}\,1_{17}\rangle$	1686.07	0.285	0.285	0.00
$\lvert 0\rangle \to \lvert 1_{34}\,1_{15}\rangle$	1686.83	0.098	0.097	0.25
$\lvert 0\rangle \to \lvert 1_{42}\,1_{7}\rangle$	1706.12	0.067	0.067	0.14
$\lvert 0\rangle \to \lvert 1_{43}\,1_{7}\rangle$	1706.66	0.021	0.021	0.41
$\lvert 0\rangle \to \lvert 1_{42}\,1_{8}\rangle$	1706.84	0.007	0.007	0.24
$\lvert 0\rangle \to \lvert 1_{43}\,1_{8}\rangle$	1707.38	12.338	12.358	0.16
$\lvert 0\rangle \to \lvert 1_{27}\,1_{19}\rangle$	1720.91	0.029	0.029	0.30
$\lvert 0\rangle \to \lvert 1_{38}\,1_{12}\rangle$	1734.95	0.001	0.001	0.49
$\lvert 0\rangle \to \lvert 1_{35}\,1_{14}\rangle$	1750.66	0.384	0.385	0.14
$\lvert 0\rangle \to \lvert 1_{28}\,1_{21}\rangle$	1755.63	0.183	0.183	0.07
$\lvert 0\rangle \to \lvert 1_{40}\,1_{12}\rangle$	1757.70	0.086	0.086	0.24
$\lvert 0\rangle \to \lvert 1_{30}\,1_{17}\rangle$	1763.05	0.371	0.372	0.22
$\lvert 0\rangle \to \lvert 1_{29}\,1_{19}\rangle$	1786.47	0.005	0.005	0.11
$\lvert 0\rangle \to \lvert 1_{29}\,1_{20}\rangle$	1789.73	0.580	0.580	0.09
$\lvert 0\rangle \to \lvert 1_{31}\,1_{18}\rangle$	1797.31	0.002	0.002	1.11
$\lvert 0\rangle \to \lvert 1_{43}\,1_{10}\rangle$	1801.45	0.003	0.003	0.08
$\lvert 0\rangle \to \lvert 1_{28}\,1_{23}\rangle$	1813.39	0.011	0.011	0.23
$\lvert 0\rangle \to \lvert 1_{39}\,1_{14}\rangle$	1817.04	0.003	0.003	0.04
$\lvert 0\rangle \to \lvert 1_{31}\,1_{21}\rangle$	1823.31	0.036	0.037	0.09
$\lvert 0\rangle \to \lvert 1_{32}\,1_{21}\rangle$	1823.93	0.136	0.136	0.09
$\lvert 0\rangle \to \lvert 1_{34}\,1_{19}\rangle$	1824.86	3.406	3.415	0.27
$\lvert 0\rangle \to \lvert 1_{46}\,1_{7}\rangle$	1827.29	0.005	0.005	0.11
$\lvert 0\rangle \to \lvert 1_{38}\,1_{15}\rangle$	1840.59	0.006	0.006	0.21
$\lvert 0\rangle \to \lvert 1_{42}\,1_{12}\rangle$	1845.07	0.031	0.031	0.09
$\lvert 0\rangle \to \lvert 1_{43}\,1_{12}\rangle$	1845.61	0.028	0.028	0.36
$\lvert 0\rangle \to \lvert 1_{37}\,1_{16}\rangle$	1852.60	0.380	0.380	0.05
$\lvert 0\rangle \to \lvert 1_{33}\,1_{22}\rangle$	1862.84	0.344	0.344	0.17
$\lvert 0\rangle \to \lvert 1_{31}\,1_{23}\rangle$	1881.07	0.037	0.038	0.14
$\lvert 0\rangle \to \lvert 1_{32}\,1_{23}\rangle$	1881.69	0.138	0.138	0.15
$\lvert 0\rangle \to \lvert 1_{48}\,1_{7}\rangle$	1882.52	0.041	0.041	0.12
$\lvert 0\rangle \to \lvert 1_{48}\,1_{8}\rangle$	1883.24	21.926	21.953	0.12
$\lvert 0\rangle \to \lvert 1_{31}\,1_{24}\rangle$	1904.96	0.318	0.318	0.16
$\lvert 0\rangle \to \lvert 1_{32}\,1_{24}\rangle$	1905.58	0.085	0.085	0.16

Continued on next page

Table S2: Anthracene TD/TI RR validation data for ground-state transitions grouped by transition class. The table reports transitions up to 4000 cm^{-1} with $I^{TD} > 10^{-4}$ $cm^3\,mol^{-1}\,sr^{-1}$. Intensities are reported in units of 10^{-1} $cm^3\,mol^{-1}\,sr^{-1}$; relative differences are reported in percent. (continued)

Transition	ν	I^{TD}	I^{TI}	Δ
$\|0\rangle \to \|1_{30}\,1_{26}\rangle$	1937.25	0.043	0.043	0.15
$\|0\rangle \to \|1_{51}\,1_{7}\rangle$	1941.59	0.002	0.002	0.04
$\|0\rangle \to \|1_{51}\,1_{8}\rangle$	1942.31	0.941	0.942	0.05
$\|0\rangle \to \|1_{43}\,1_{15}\rangle$	1951.25	0.069	0.069	0.09
$\|0\rangle \to \|1_{35}\,1_{22}\rangle$	1963.87	1.403	1.405	0.19
$\|0\rangle \to \|1_{31}\,1_{28}\rangle$	1964.08	0.033	0.033	0.03
$\|0\rangle \to \|1_{32}\,1_{28}\rangle$	1964.70	0.127	0.127	0.02
$\|0\rangle \to \|1_{55}\,1_{4}\rangle$	1964.71	0.001	0.001	0.09
$\|0\rangle \to \|1_{46}\,1_{12}\rangle$	1966.23	0.023	0.023	0.07
$\|0\rangle \to \|1_{41}\,1_{16}\rangle$	1967.98	1.334	1.334	0.03
$\|0\rangle \to \|1_{48}\,1_{10}\rangle$	1977.30	0.006	0.006	0.08
$\|0\rangle \to \|1_{34}\,1_{27}\rangle$	1977.53	0.004	0.004	0.17
$\|0\rangle \to \|1_{38}\,1_{19}\rangle$	1978.62	0.978	0.979	0.11
$\|0\rangle \to \|1_{44}\,1_{14}\rangle$	1978.78	0.500	0.501	0.29
$\|0\rangle \to \|1_{40}\,1_{19}\rangle$	2001.37	0.099	0.099	0.19
$\|0\rangle \to \|1_{48}\,1_{12}\rangle$	2021.46	0.053	0.053	0.24
$\|0\rangle \to \|1_{39}\,1_{22}\rangle$	2030.25	0.027	0.027	0.12
$\|0\rangle \to \|1_{45}\,1_{16}\rangle$	2031.16	0.272	0.272	0.00
$\|0\rangle \to \|1_{53}\,1_{8}\rangle$	2056.80	0.044	0.044	0.61
$\|0\rangle \to \|1_{54}\,1_{7}\rangle$	2074.84	0.101	0.101	0.19
$\|0\rangle \to \|1_{54}\,1_{8}\rangle$	2075.56	0.028	0.028	0.12
$\|0\rangle \to \|1_{47}\,1_{14}\rangle$	2078.15	0.036	0.036	0.03
$\|0\rangle \to \|1_{36}\,1_{27}\rangle$	2080.23	0.107	0.107	0.55
$\|0\rangle \to \|1_{51}\,1_{12}\rangle$	2080.54	0.002	0.002	0.01
$\|0\rangle \to \|1_{42}\,1_{19}\rangle$	2088.74	0.014	0.014	0.23
$\|0\rangle \to \|1_{43}\,1_{19}\rangle$	2089.28	20.586	20.601	0.07
$\|0\rangle \to \|1_{43}\,1_{20}\rangle$	2092.54	0.002	0.002	0.13
$\|0\rangle \to \|1_{37}\,1_{25}\rangle$	2113.36	0.023	0.023	0.35
$\|0\rangle \to \|1_{49}\,1_{14}\rangle$	2120.04	0.063	0.064	0.37
$\|0\rangle \to \|1_{48}\,1_{15}\rangle$	2127.10	0.078	0.079	0.75
$\|0\rangle \to \|1_{38}\,1_{27}\rangle$	2131.29	0.001	0.001	0.42
$\|0\rangle \to \|1_{56}\,1_{7}\rangle$	2136.81	0.102	0.102	0.36
$\|0\rangle \to \|1_{56}\,1_{8}\rangle$	2137.53	0.506	0.506	0.06
$\|0\rangle \to \|1_{40}\,1_{27}\rangle$	2154.04	0.143	0.144	0.58

Continued on next page

Table S2: Anthracene TD/TI RR validation data for ground-state transitions grouped by transition class. The table reports transitions up to 4000 cm^{-1} with $I^{\mathrm{TD}} > 10^{-4}$ cm^3 mol^{-1} sr^{-1}. Intensities are reported in units of 10^{-1} cm^3 mol^{-1} sr^{-1}; relative differences are reported in percent. (continued)

Transition	ν	I^{TD}	I^{TI}	Δ
$\vert 0\rangle \to \vert 1_{35}\, 1_{33}\rangle$	2160.56	1.449	1.450	0.10
$\vert 0\rangle \to \vert 1_{50}\, 1_{16}\rangle$	2178.59	0.642	0.642	0.00
$\vert 0\rangle \to \vert 1_{51}\, 1_{15}\rangle$	2186.18	0.093	0.093	0.02
$\vert 0\rangle \to \vert 1_{44}\, 1_{22}\rangle$	2191.99	1.175	1.176	0.07
$\vert 0\rangle \to \vert 1_{54}\, 1_{12}\rangle$	2213.78	0.039	0.039	0.30
$\vert 0\rangle \to \vert 1_{39}\, 1_{33}\rangle$	2226.94	0.100	0.100	0.03
$\vert 0\rangle \to \vert 1_{41}\, 1_{25}\rangle$	2228.74	0.155	0.156	0.30
$\vert 0\rangle \to \vert 1_{38}\, 1_{34}\rangle$	2235.23	0.194	0.194	0.07
$\vert 0\rangle \to \vert 1_{42}\, 1_{27}\rangle$	2241.41	0.290	0.290	0.25
$\vert 0\rangle \to \vert 1_{43}\, 1_{27}\rangle$	2241.95	0.024	0.025	0.31
$\vert 0\rangle \to \vert 1_{52}\, 1_{14}\rangle$	2245.46	0.039	0.039	0.23
$\vert 0\rangle \to \vert 1_{40}\, 1_{34}\rangle$	2257.98	0.013	0.013	0.06
$\vert 0\rangle \to \vert 1_{48}\, 1_{19}\rangle$	2265.13	37.816	37.912	0.25
$\vert 0\rangle \to \vert 1_{48}\, 1_{20}\rangle$	2268.40	0.004	0.004	0.03
$\vert 0\rangle \to \vert 1_{56}\, 1_{12}\rangle$	2275.75	0.320	0.320	0.06
$\vert 0\rangle \to \vert 1_{47}\, 1_{22}\rangle$	2291.36	0.039	0.039	0.04
$\vert 0\rangle \to \vert 1_{45}\, 1_{25}\rangle$	2291.92	0.060	0.060	0.27
$\vert 0\rangle \to \vert 1_{53}\, 1_{15}\rangle$	2300.66	0.007	0.007	0.08
$\vert 0\rangle \to \vert 1_{43}\, 1_{29}\rangle$	2307.51	0.004	0.004	0.06
$\vert 0\rangle \to \vert 1_{51}\, 1_{19}\rangle$	2324.21	1.559	1.561	0.13
$\vert 0\rangle \to \vert 1_{39}\, 1_{35}\rangle$	2327.96	0.050	0.050	0.38
$\vert 0\rangle \to \vert 1_{49}\, 1_{22}\rangle$	2333.25	0.005	0.005	0.11
$\vert 0\rangle \to \vert 1_{42}\, 1_{34}\rangle$	2345.35	0.002	0.002	0.10
$\vert 0\rangle \to \vert 1_{43}\, 1_{34}\rangle$	2345.89	3.454	3.452	0.08
$\vert 0\rangle \to \vert 1_{40}\, 1_{36}\rangle$	2360.69	0.094	0.094	0.20
$\vert 0\rangle \to \vert 1_{46}\, 1_{27}\rangle$	2362.57	0.160	0.160	0.04
$\vert 0\rangle \to \vert 1_{56}\, 1_{15}\rangle$	2381.39	0.005	0.005	0.21
$\vert 0\rangle \to \vert 1_{44}\, 1_{33}\rangle$	2388.68	0.816	0.818	0.20
$\vert 0\rangle \to \vert 1_{55}\, 1_{16}\rangle$	2393.05	6.839	6.829	0.14
$\vert 0\rangle \to \vert 1_{40}\, 1_{38}\rangle$	2411.74	0.004	0.004	0.32
$\vert 0\rangle \to \vert 1_{48}\, 1_{27}\rangle$	2417.80	0.046	0.047	0.17
$\vert 0\rangle \to \vert 1_{53}\, 1_{19}\rangle$	2438.69	0.235	0.235	0.05
$\vert 0\rangle \to \vert 1_{50}\, 1_{25}\rangle$	2439.35	0.030	0.030	0.48
$\vert 0\rangle \to \vert 1_{42}\, 1_{36}\rangle$	2448.06	0.208	0.208	0.13

Continued on next page

Table S2: Anthracene TD/TI RR validation data for ground-state transitions grouped by transition class. The table reports transitions up to 4000 cm^{-1} with $I^{TD} > 10^{-4}$ $cm^3\,mol^{-1}\,sr^{-1}$. Intensities are reported in units of 10^{-1} $cm^3\,mol^{-1}\,sr^{-1}$; relative differences are reported in percent. (continued)

Transition	ν	I^{TD}	I^{TI}	Δ
$\|0\rangle \to \|1_{54}\,1_{19}\rangle$	2457.46	0.059	0.059	0.13
$\|0\rangle \to \|1_{52}\,1_{22}\rangle$	2458.67	0.020	0.020	0.18
$\|0\rangle \to \|1_{51}\,1_{27}\rangle$	2476.88	0.002	0.002	0.01
$\|0\rangle \to \|1_{48}\,1_{29}\rangle$	2483.36	0.007	0.007	0.03
$\|0\rangle \to \|1_{41}\,1_{37}\rangle$	2486.74	0.440	0.441	0.24
$\|0\rangle \to \|1_{47}\,1_{33}\rangle$	2488.04	0.017	0.017	1.34
$\|0\rangle \to \|1_{44}\,1_{35}\rangle$	2489.71	3.415	3.412	0.09
$\|0\rangle \to \|1_{43}\,1_{38}\rangle$	2499.66	0.571	0.571	0.05
$\|0\rangle \to \|1_{56}\,1_{19}\rangle$	2519.43	1.067	1.068	0.09
$\|0\rangle \to \|1_{48}\,1_{34}\rangle$	2521.75	6.673	6.662	0.16
$\|0\rangle \to \|1_{42}\,1_{40}\rangle$	2521.87	0.335	0.336	0.17
$\|0\rangle \to \|1_{43}\,1_{40}\rangle$	2522.40	0.082	0.082	0.30
$\|0\rangle \to \|1_{49}\,1_{33}\rangle$	2529.93	0.135	0.135	0.40
$\|0\rangle \to \|1_{45}\,1_{37}\rangle$	2549.92	0.116	0.117	0.18
$\|0\rangle \to \|1_{44}\,1_{39}\rangle$	2556.08	0.045	0.045	0.32
$\|0\rangle \to \|1_{46}\,1_{36}\rangle$	2569.22	0.151	0.152	0.44
$\|0\rangle \to \|1_{51}\,1_{34}\rangle$	2580.82	0.366	0.366	0.01
$\|0\rangle \to \|1_{47}\,1_{35}\rangle$	2589.07	0.057	0.056	1.04
$\|0\rangle \to \|1_{43}\,1_{42}\rangle$	2609.78	0.011	0.011	0.20
$\|0\rangle \to \|1_{54}\,1_{27}\rangle$	2610.12	0.517	0.519	0.51
$\|0\rangle \to \|1_{46}\,1_{40}\rangle$	2643.03	0.176	0.176	0.37
$\|0\rangle \to \|1_{55}\,1_{25}\rangle$	2653.81	0.578	0.580	0.45
$\|0\rangle \to \|1_{52}\,1_{33}\rangle$	2655.35	0.046	0.046	0.11
$\|0\rangle \to \|1_{47}\,1_{39}\rangle$	2655.45	0.051	0.051	0.29
$\|0\rangle \to \|1_{45}\,1_{41}\rangle$	2665.30	0.446	0.447	0.15
$\|0\rangle \to \|1_{56}\,1_{27}\rangle$	2672.09	0.017	0.017	0.01
$\|0\rangle \to \|1_{48}\,1_{38}\rangle$	2675.51	1.240	1.242	0.22
$\|0\rangle \to \|1_{53}\,1_{34}\rangle$	2695.31	0.052	0.053	0.21
$\|0\rangle \to \|1_{49}\,1_{39}\rangle$	2697.34	0.001	0.001	0.14
$\|0\rangle \to \|1_{50}\,1_{37}\rangle$	2697.35	0.252	0.252	0.11
$\|0\rangle \to \|1_{48}\,1_{40}\rangle$	2698.26	0.154	0.154	0.04
$\|0\rangle \to \|1_{54}\,1_{34}\rangle$	2714.07	0.008	0.008	0.04
$\|0\rangle \to \|1_{46}\,1_{42}\rangle$	2730.40	0.444	0.445	0.17
$\|0\rangle \to \|1_{51}\,1_{38}\rangle$	2734.58	0.048	0.048	0.19

Continued on next page

Table S2: Anthracene TD/TI RR validation data for ground-state transitions grouped by transition class. The table reports transitions up to $4000\,\mathrm{cm}^{-1}$ with $I^{\mathrm{TD}} > 10^{-4}$ $\mathrm{cm}^3\,\mathrm{mol}^{-1}\,\mathrm{sr}^{-1}$. Intensities are reported in units of 10^{-1} $\mathrm{cm}^3\,\mathrm{mol}^{-1}\,\mathrm{sr}^{-1}$; relative differences are reported in percent. (continued)

Transition	ν	I^{TD}	I^{TI}	Δ
$\lvert 0\rangle \to \lvert 1_{52}\,1_{35}\rangle$	2756.38	0.017	0.017	1.13
$\lvert 0\rangle \to \lvert 1_{51}\,1_{40}\rangle$	2757.33	0.007	0.007	0.07
$\lvert 0\rangle \to \lvert 1_{56}\,1_{34}\rangle$	2776.04	0.143	0.142	0.23
$\lvert 0\rangle \to \lvert 1_{48}\,1_{42}\rangle$	2785.63	0.021	0.021	0.03
$\lvert 0\rangle \to \lvert 1_{48}\,1_{43}\rangle$	2786.17	24.977	24.954	0.09
$\lvert 0\rangle \to \lvert 1_{50}\,1_{41}\rangle$	2812.73	0.695	0.697	0.22
$\lvert 0\rangle \to \lvert 1_{54}\,1_{36}\rangle$	2816.77	0.327	0.327	0.05
$\lvert 0\rangle \to \lvert 1_{47}\,1_{44}\rangle$	2817.19	0.226	0.224	0.84
$\lvert 0\rangle \to \lvert 1_{52}\,1_{39}\rangle$	2822.76	0.084	0.084	0.32
$\lvert 0\rangle \to \lvert 1_{51}\,1_{42}\rangle$	2844.71	0.001	0.001	0.03
$\lvert 0\rangle \to \lvert 1_{51}\,1_{43}\rangle$	2845.24	2.247	2.247	0.01
$\lvert 0\rangle \to \lvert 1_{53}\,1_{38}\rangle$	2849.07	0.006	0.006	0.72
$\lvert 0\rangle \to \lvert 1_{49}\,1_{44}\rangle$	2859.08	0.001	0.001	0.65
$\lvert 0\rangle \to \lvert 1_{54}\,1_{38}\rangle$	2867.83	0.002	0.002	0.19
$\lvert 0\rangle \to \lvert 1_{50}\,1_{45}\rangle$	2875.91	0.389	0.390	0.26
$\lvert 0\rangle \to \lvert 1_{56}\,1_{36}\rangle$	2878.74	0.004	0.004	0.16
$\lvert 0\rangle \to \lvert 1_{54}\,1_{40}\rangle$	2890.58	0.642	0.642	0.05
$\lvert 0\rangle \to \lvert 1_{55}\,1_{37}\rangle$	2911.81	2.250	2.252	0.11
$\lvert 0\rangle \to \lvert 1_{56}\,1_{38}\rangle$	2929.80	0.039	0.039	0.09
$\lvert 0\rangle \to \lvert 1_{56}\,1_{40}\rangle$	2952.55	0.006	0.006	0.77
$\lvert 0\rangle \to \lvert 1_{49}\,1_{47}\rangle$	2958.44	0.015	0.015	0.95
$\lvert 0\rangle \to \lvert 1_{53}\,1_{43}\rangle$	2959.73	0.079	0.079	0.30
$\lvert 0\rangle \to \lvert 1_{54}\,1_{42}\rangle$	2977.95	1.752	1.757	0.29
$\lvert 0\rangle \to \lvert 1_{54}\,1_{43}\rangle$	2978.49	0.048	0.048	0.17
$\lvert 0\rangle \to \lvert 1_{52}\,1_{44}\rangle$	2984.50	0.007	0.007	0.88
$\lvert 0\rangle \to \lvert 1_{51}\,1_{48}\rangle$	3021.10	2.128	2.132	0.22
$\lvert 0\rangle \to \lvert 1_{55}\,1_{41}\rangle$	3027.19	8.758	8.772	0.16
$\lvert 0\rangle \to \lvert 1_{56}\,1_{42}\rangle$	3039.92	0.033	0.033	0.26
$\lvert 0\rangle \to \lvert 1_{56}\,1_{43}\rangle$	3040.46	0.866	0.866	0.02
$\lvert 0\rangle \to \lvert 1_{52}\,1_{47}\rangle$	3083.86	0.040	0.040	0.72
$\lvert 0\rangle \to \lvert 1_{55}\,1_{45}\rangle$	3090.37	2.539	2.543	0.13
$\lvert 0\rangle \to \lvert 1_{54}\,1_{46}\rangle$	3099.11	0.890	0.894	0.39
$\lvert 0\rangle \to \lvert 1_{53}\,1_{48}\rangle$	3135.58	0.144	0.144	0.20
$\lvert 0\rangle \to \lvert 1_{54}\,1_{48}\rangle$	3154.34	0.090	0.090	0.00

Continued on next page

Table S2: Anthracene TD/TI RR validation data for ground-state transitions grouped by transition class. The table reports transitions up to 4000 cm^{-1} with $I^{\mathrm{TD}} > 10^{-4}$ $\mathrm{cm}^3\,\mathrm{mol}^{-1}\,\mathrm{sr}^{-1}$. Intensities are reported in units of 10^{-1} $\mathrm{cm}^3\,\mathrm{mol}^{-1}\,\mathrm{sr}^{-1}$; relative differences are reported in percent. (continued)

Transition	ν	I^{TD}	I^{TI}	Δ
$\lvert 0\rangle \rightarrow \lvert 1_{56}\,1_{46}\rangle$	3161.08	0.022	0.022	0.19
$\lvert 0\rangle \rightarrow \lvert 1_{53}\,1_{51}\rangle$	3194.66	0.035	0.035	0.01
$\lvert 0\rangle \rightarrow \lvert 1_{54}\,1_{51}\rangle$	3213.42	0.004	0.004	0.03
$\lvert 0\rangle \rightarrow \lvert 1_{56}\,1_{48}\rangle$	3216.31	1.623	1.622	0.09
$\lvert 0\rangle \rightarrow \lvert 1_{55}\,1_{50}\rangle$	3237.80	4.041	4.044	0.07
$\lvert 0\rangle \rightarrow \lvert 1_{56}\,1_{51}\rangle$	3275.39	0.069	0.069	0.01
$\lvert 0\rangle \rightarrow \lvert 1_{56}\,1_{53}\rangle$	3389.87	0.007	0.007	0.22
$\lvert 0\rangle \rightarrow \lvert 1_{56}\,1_{54}\rangle$	3408.64	0.046	0.046	0.11
$\lvert 0\rangle \rightarrow \lvert 1_{60}\,1_{4}\rangle$	3437.05	0.001	0.001	1.38
$\lvert 0\rangle \rightarrow \lvert 1_{58}\,1_{8}\rangle$	3595.93	0.016	0.016	0.91
$\lvert 0\rangle \rightarrow \lvert 1_{59}\,1_{8}\rangle$	3600.67	0.001	0.001	0.09
$\lvert 0\rangle \rightarrow \lvert 1_{63}\,1_{8}\rangle$	3619.98	0.001	0.001	0.04
$\lvert 0\rangle \rightarrow \lvert 1_{66}\,1_{8}\rangle$	3632.66	0.005	0.005	2.07
$\lvert 0\rangle \rightarrow \lvert 1_{61}\,1_{14}\rangle$	3822.35	0.002	0.002	1.44
$\lvert 0\rangle \rightarrow \lvert 1_{58}\,1_{15}\rangle$	3839.80	0.003	0.003	0.04
$\lvert 0\rangle \rightarrow \lvert 1_{62}\,1_{15}\rangle$	3849.29	0.001	0.001	0.32
$\lvert 0\rangle \rightarrow \lvert 1_{57}\,1_{16}\rangle$	3858.85	0.004	0.004	0.77
$\lvert 0\rangle \rightarrow \lvert 1_{60}\,1_{16}\rangle$	3865.39	0.002	0.002	1.98
$\lvert 0\rangle \rightarrow \lvert 1_{64}\,1_{16}\rangle$	3884.82	0.017	0.017	0.68
$\lvert 0\rangle \rightarrow \lvert 1_{58}\,1_{19}\rangle$	3977.83	0.030	0.030	0.53
$\lvert 0\rangle \rightarrow \lvert 1_{59}\,1_{19}\rangle$	3982.57	0.003	0.003	0.02

1.5 Resonance Raman transition information for hot bands

Anthracene was used as the reference TD/TI RR validation case for the hot-band manifolds. The default-prescreening validation set is reported in Table S3 and grouped by transition class. The table contains the top transitions retained for each class with $I^{\mathrm{TD}} > 10^{-4}$ $\mathrm{cm}^3\,\mathrm{mol}^{-1}\,\mathrm{sr}^{-1}$, with a maximum of 100 transitions retained for each class. For each transition, the table reports the TD RR intensity, the corresponding default-prescreening TI RR intensity, and the relative difference $\Delta = \Delta I/I = \left|I^{\mathrm{TD}} - I^{\mathrm{TI}}\right|/I^{\mathrm{TI}}$.

Table S3: Anthracene hot-band TD/TI RR validation data grouped by transition class. The table reports the top default-intense transitions retained for each class with $I^{\mathrm{TD}} > 10^{-4}$ cm^3 mol^{-1} sr^{-1}. Intensities are reported in units of 10^{-1} cm^3 mol^{-1} sr^{-1}; relative differences are reported in percent.

Transition	ν	I^{TD}	I^{TI}	Δ
$1_i \to 2_i$				
$\vert 1_3\rangle \to \vert 2_3\rangle$	236.38	0.003	0.003	0.08
$\vert 1_7\rangle \to \vert 2_7\rangle$	401.51	0.867	0.866	0.10
$\vert 1_8\rangle \to \vert 2_8\rangle$	402.22	225.242	225.420	0.08
$\vert 1_{10}\rangle \to \vert 2_{10}\rangle$	496.29	0.143	0.142	0.41
$\vert 1_{12}\rangle \to \vert 2_{12}\rangle$	540.45	1.150	1.147	0.28
$\vert 1_{15}\rangle \to \vert 2_{15}\rangle$	646.09	2.997	2.994	0.11
$\vert 1_{19}\rangle \to \vert 2_{19}\rangle$	784.12	412.551	412.750	0.05
$\vert 1_{20}\rangle \to \vert 2_{20}\rangle$	787.38	0.082	0.082	0.38
$\vert 1_{27}\rangle \to \vert 2_{27}\rangle$	936.79	1.036	1.030	0.54
$\vert 1_{29}\rangle \to \vert 2_{29}\rangle$	1002.35	0.159	0.158	0.44
$\vert 1_{34}\rangle \to \vert 2_{34}\rangle$	1040.73	74.671	74.560	0.15
$\vert 1_{36}\rangle \to \vert 2_{36}\rangle$	1143.44	0.010	0.010	0.88
$\vert 1_{38}\rangle \to \vert 2_{38}\rangle$	1194.50	20.085	20.093	0.04
$\vert 1_{40}\rangle \to \vert 2_{40}\rangle$	1217.25	3.378	3.354	0.73
$\vert 1_{41}\rangle \to \vert 2_{41}\rangle$	1301.06	0.001	0.001	0.02
$\vert 1_{42}\rangle \to \vert 2_{42}\rangle$	1304.62	0.446	0.446	0.18
$\vert 1_{43}\rangle \to \vert 2_{43}\rangle$	1305.16	369.071	369.130	0.02
$\vert 1_{46}\rangle \to \vert 2_{46}\rangle$	1425.78	0.012	0.012	0.80
$\vert 1_{48}\rangle \to \vert 2_{48}\rangle$	1481.01	573.095	572.970	0.02
$\vert 1_{51}\rangle \to \vert 2_{51}\rangle$	1540.09	38.564	38.560	0.01
$\vert 1_{53}\rangle \to \vert 2_{53}\rangle$	1654.57	2.466	2.468	0.10
$\vert 1_{54}\rangle \to \vert 2_{54}\rangle$	1673.33	1.851	1.845	0.34
$\vert 1_{56}\rangle \to \vert 2_{56}\rangle$	1735.30	34.760	34.614	0.42
$\vert 1_{58}\rangle \to \vert 2_{58}\rangle$	3193.70	0.784	0.783	0.12
$\vert 1_{59}\rangle \to \vert 2_{59}\rangle$	3198.45	0.080	0.079	0.49
$\vert 1_{62}\rangle \to \vert 2_{62}\rangle$	3203.20	0.008	0.008	1.91
$\vert 1_{63}\rangle \to \vert 2_{63}\rangle$	3217.75	0.070	0.070	0.33
$\vert 1_{66}\rangle \to \vert 2_{66}\rangle$	3230.43	0.254	0.254	0.06
$1_i \to 3_i$				
$\vert 1_1\rangle \to \vert 3_1\rangle$	183.17	15.877	15.825	0.33
$\vert 1_2\rangle \to \vert 3_2\rangle$	249.36	20.900	20.909	0.04

Continued on next page

Table S3: Anthracene hot-band TD/TI RR validation data grouped by transition class (continued).

Transition	ν	I^{TD}	I^{TI}	Δ
$\|1_3\rangle \to \|3_3\rangle$	472.76	33.628	33.614	0.04
$\|1_4\rangle \to \|3_4\rangle$	477.16	0.653	0.653	0.09
$\|1_5\rangle \to \|3_5\rangle$	548.53	59.947	60.013	0.11
$\|1_6\rangle \to \|3_6\rangle$	788.04	40.883	40.885	0.01
$\|1_7\rangle \to \|3_7\rangle$	803.01	2.201	2.202	0.03
$\|1_8\rangle \to \|3_8\rangle$	804.45	14.618	14.628	0.07
$\|1_9\rangle \to \|3_9\rangle$	980.63	20.522	20.488	0.17
$\|1_{10}\rangle \to \|3_{10}\rangle$	992.58	18.294	18.320	0.14
$\|1_{11}\rangle \to \|3_{11}\rangle$	1027.46	50.882	50.822	0.12
$\|1_{12}\rangle \to \|3_{12}\rangle$	1080.90	1.649	1.647	0.13
$\|1_{13}\rangle \to \|3_{13}\rangle$	1204.48	104.860	104.450	0.39
$\|1_{14}\rangle \to \|3_{14}\rangle$	1239.74	0.235	0.235	0.14
$\|1_{15}\rangle \to \|3_{15}\rangle$	1292.18	1.537	1.538	0.06
$\|1_{16}\rangle \to \|3_{16}\rangle$	1333.84	0.501	0.500	0.07
$\|1_{17}\rangle \to \|3_{17}\rangle$	1511.87	3.793	3.788	0.12
$\|1_{18}\rangle \to \|3_{18}\rangle$	1562.85	17.716	17.734	0.10
$\|1_{19}\rangle \to \|3_{19}\rangle$	1568.25	36.092	36.119	0.07
$\|1_{20}\rangle \to \|3_{20}\rangle$	1574.77	12.698	12.676	0.17
$\|1_{21}\rangle \to \|3_{21}\rangle$	1614.86	9.977	9.968	0.10
$\|1_{22}\rangle \to \|3_{22}\rangle$	1666.16	0.073	0.073	0.71
$\|1_{23}\rangle \to \|3_{23}\rangle$	1730.38	8.898	8.887	0.12
$\|1_{24}\rangle \to \|3_{24}\rangle$	1778.16	9.284	9.279	0.06
$\|1_{25}\rangle \to \|3_{25}\rangle$	1855.36	0.119	0.119	0.23
$\|1_{26}\rangle \to \|3_{26}\rangle$	1860.28	6.263	6.268	0.07
$\|1_{27}\rangle \to \|3_{27}\rangle$	1873.58	0.373	0.371	0.42
$\|1_{28}\rangle \to \|3_{28}\rangle$	1896.41	5.892	5.895	0.04
$\|1_{29}\rangle \to \|3_{29}\rangle$	2004.70	4.990	4.983	0.15
$\|1_{30}\rangle \to \|3_{30}\rangle$	2014.22	4.778	4.770	0.17
$\|1_{31}\rangle \to \|3_{31}\rangle$	2031.76	8.816	8.803	0.14
$\|1_{32}\rangle \to \|3_{32}\rangle$	2033.00	8.724	8.707	0.20
$\|1_{33}\rangle \to \|3_{33}\rangle$	2059.53	0.081	0.081	0.51
$\|1_{34}\rangle \to \|3_{34}\rangle$	2081.47	1.008	1.003	0.47
$\|1_{35}\rangle \to \|3_{35}\rangle$	2261.59	4.746	4.757	0.23
$\|1_{36}\rangle \to \|3_{36}\rangle$	2286.88	0.218	0.218	0.18
$\|1_{37}\rangle \to \|3_{37}\rangle$	2371.36	0.101	0.101	0.06
$\|1_{38}\rangle \to \|3_{38}\rangle$	2389.00	0.041	0.042	0.36

Continued on next page

Table S3: Anthracene hot-band TD/TI RR validation data grouped by transition class (continued).

Transition	ν	I^{TD}	I^{TI}	Δ
$\|1_{39}\rangle \to \|3_{39}\rangle$	2394.34	0.017	0.017	0.06
$\|1_{40}\rangle \to \|3_{40}\rangle$	2434.49	0.216	0.215	0.44
$\|1_{41}\rangle \to \|3_{41}\rangle$	2602.12	1.839	1.842	0.15
$\|1_{42}\rangle \to \|3_{42}\rangle$	2609.24	1.703	1.705	0.10
$\|1_{43}\rangle \to \|3_{43}\rangle$	2610.32	20.331	20.344	0.06
$\|1_{44}\rangle \to \|3_{44}\rangle$	2717.83	3.580	3.557	0.65
$\|1_{45}\rangle \to \|3_{45}\rangle$	2728.48	0.020	0.020	0.54
$\|1_{46}\rangle \to \|3_{46}\rangle$	2851.56	0.482	0.482	0.06
$\|1_{47}\rangle \to \|3_{47}\rangle$	2916.55	0.147	0.146	0.25
$\|1_{48}\rangle \to \|3_{48}\rangle$	2962.02	52.556	52.796	0.45
$\|1_{49}\rangle \to \|3_{49}\rangle$	3000.33	0.152	0.152	0.20
$\|1_{50}\rangle \to \|3_{50}\rangle$	3023.33	0.169	0.169	0.29
$\|1_{51}\rangle \to \|3_{51}\rangle$	3080.17	1.131	1.131	0.06
$\|1_{52}\rangle \to \|3_{52}\rangle$	3251.18	1.232	1.229	0.22
$\|1_{53}\rangle \to \|3_{53}\rangle$	3309.14	0.078	0.079	0.39
$\|1_{54}\rangle \to \|3_{54}\rangle$	3346.67	6.347	6.338	0.14
$\|1_{55}\rangle \to \|3_{55}\rangle$	3452.26	19.873	19.695	0.90
$\|1_{56}\rangle \to \|3_{56}\rangle$	3470.60	0.080	0.079	1.16
$\|1_{61}\rangle \to \|3_{61}\rangle$	6404.96	0.001	0.001	0.32
$1_i \to 1_i 1_j$				
$\|1_{1}\rangle \to \|1_{19}\, 1_{1}\rangle$	784.12	287.371	286.970	0.14
$\|1_{2}\rangle \to \|1_{19}\, 1_{2}\rangle$	784.12	287.743	287.140	0.21
$\|1_{3}\rangle \to \|1_{19}\, 1_{3}\rangle$	784.12	293.420	292.580	0.29
$\|1_{4}\rangle \to \|1_{19}\, 1_{4}\rangle$	784.12	281.374	281.130	0.09
$\|1_{5}\rangle \to \|1_{19}\, 1_{5}\rangle$	784.12	285.800	284.240	0.55
$\|1_{6}\rangle \to \|1_{19}\, 1_{6}\rangle$	784.12	298.940	297.680	0.42
$\|1_{7}\rangle \to \|1_{19}\, 1_{7}\rangle$	784.12	285.847	285.010	0.29
$\|1_{9}\rangle \to \|1_{19}\, 1_{9}\rangle$	784.12	297.997	295.120	0.97
$\|1_{10}\rangle \to \|1_{19}\, 1_{10}\rangle$	784.12	296.607	294.970	0.55
$\|1_{11}\rangle \to \|1_{19}\, 1_{11}\rangle$	784.12	303.195	299.450	1.25
$\|1_{12}\rangle \to \|1_{19}\, 1_{12}\rangle$	784.12	286.533	285.530	0.35
$\|1_{13}\rangle \to \|1_{19}\, 1_{13}\rangle$	784.12	299.659	296.890	0.93
$\|1_{14}\rangle \to \|1_{19}\, 1_{14}\rangle$	784.12	281.048	280.240	0.29
$\|1_{15}\rangle \to \|1_{19}\, 1_{15}\rangle$	784.12	287.210	287.090	0.04
$\|1_{17}\rangle \to \|1_{19}\, 1_{17}\rangle$	784.12	293.224	291.840	0.47

Continued on next page

Table S3: Anthracene hot-band TD/TI RR validation data grouped by transition class (continued).

Transition	ν	I^{TD}	I^{TI}	Δ
$\|1_{18}\rangle \to \|1_{19}\,1_{18}\rangle$	784.12	305.598	303.490	0.69
$\|1_{20}\rangle \to \|1_{20}\,1_{19}\rangle$	784.12	299.414	297.660	0.59
$\|1_{21}\rangle \to \|1_{21}\,1_{19}\rangle$	784.12	299.618	297.950	0.56
$\|1_{23}\rangle \to \|1_{23}\,1_{19}\rangle$	784.12	303.538	301.840	0.56
$\|1_{24}\rangle \to \|1_{24}\,1_{19}\rangle$	784.12	299.152	297.530	0.55
$\|1_{25}\rangle \to \|1_{25}\,1_{19}\rangle$	784.12	280.789	279.950	0.30
$\|1_{26}\rangle \to \|1_{26}\,1_{19}\rangle$	784.12	302.236	300.500	0.58
$\|1_{27}\rangle \to \|1_{27}\,1_{19}\rangle$	784.12	281.416	280.420	0.36
$\|1_{28}\rangle \to \|1_{28}\,1_{19}\rangle$	784.12	301.826	300.130	0.56
$\|1_{29}\rangle \to \|1_{29}\,1_{19}\rangle$	784.12	300.586	299.040	0.52
$\|1_{30}\rangle \to \|1_{30}\,1_{19}\rangle$	784.12	300.607	298.890	0.57
$\|1_{31}\rangle \to \|1_{31}\,1_{19}\rangle$	784.12	307.464	305.590	0.61
$\|1_{32}\rangle \to \|1_{32}\,1_{19}\rangle$	784.12	306.813	304.300	0.83
$\|1_{36}\rangle \to \|1_{36}\,1_{19}\rangle$	784.12	280.705	279.860	0.30
$\|1_{42}\rangle \to \|1_{42}\,1_{19}\rangle$	784.12	281.153	280.870	0.10
$\|1_{49}\rangle \to \|1_{49}\,1_{19}\rangle$	784.12	284.435	284.160	0.10
$\|1_{51}\rangle \to \|1_{51}\,1_{19}\rangle$	784.12	280.848	280.750	0.03
$\|1_{52}\rangle \to \|1_{52}\,1_{19}\rangle$	784.12	297.971	296.750	0.41
$\|1_{54}\rangle \to \|1_{54}\,1_{19}\rangle$	784.12	293.656	292.170	0.51
$\|1_{60}\rangle \to \|1_{60}\,1_{19}\rangle$	784.12	278.998	278.480	0.19
$\|1_{61}\rangle \to \|1_{61}\,1_{19}\rangle$	784.12	279.541	278.880	0.24
$\|1_{1}\rangle \to \|1_{48}\,1_{1}\rangle$	1481.01	459.576	459.880	0.07
$\|1_{2}\rangle \to \|1_{48}\,1_{2}\rangle$	1481.01	458.879	459.170	0.06
$\|1_{3}\rangle \to \|1_{48}\,1_{3}\rangle$	1481.01	462.436	462.650	0.05
$\|1_{4}\rangle \to \|1_{48}\,1_{4}\rangle$	1481.01	456.361	456.760	0.09
$\|1_{5}\rangle \to \|1_{48}\,1_{5}\rangle$	1481.01	448.711	448.460	0.06
$\|1_{6}\rangle \to \|1_{48}\,1_{6}\rangle$	1481.01	464.788	464.710	0.02
$\|1_{7}\rangle \to \|1_{48}\,1_{7}\rangle$	1481.01	460.024	460.500	0.10
$\|1_{8}\rangle \to \|1_{48}\,1_{8}\rangle$	1481.01	377.981	378.260	0.07
$\|1_{9}\rangle \to \|1_{48}\,1_{9}\rangle$	1481.01	466.943	466.880	0.01
$\|1_{10}\rangle \to \|1_{48}\,1_{10}\rangle$	1481.01	464.935	465.030	0.02
$\|1_{11}\rangle \to \|1_{48}\,1_{11}\rangle$	1481.01	464.914	464.040	0.19
$\|1_{12}\rangle \to \|1_{48}\,1_{12}\rangle$	1481.01	460.611	461.080	0.10
$\|1_{13}\rangle \to \|1_{48}\,1_{13}\rangle$	1481.01	449.930	448.670	0.28
$\|1_{14}\rangle \to \|1_{48}\,1_{14}\rangle$	1481.01	455.366	455.870	0.11
$\|1_{15}\rangle \to \|1_{48}\,1_{15}\rangle$	1481.01	460.898	461.280	0.08

Continued on next page

Table S3: Anthracene hot-band TD/TI RR validation data grouped by transition class (continued).

Transition	ν	I^{TD}	I^{TI}	Δ
$\lvert 1_{16}\rangle \to \lvert 1_{48}\, 1_{16}\rangle$	1481.01	439.222	439.630	0.09
$\lvert 1_{17}\rangle \to \lvert 1_{48}\, 1_{17}\rangle$	1481.01	465.387	465.680	0.06
$\lvert 1_{18}\rangle \to \lvert 1_{48}\, 1_{18}\rangle$	1481.01	471.045	470.720	0.07
$\lvert 1_{19}\rangle \to \lvert 1_{48}\, 1_{19}\rangle$	1481.01	301.344	301.560	0.07
$\lvert 1_{20}\rangle \to \lvert 1_{48}\, 1_{20}\rangle$	1481.01	467.621	467.550	0.02
$\lvert 1_{21}\rangle \to \lvert 1_{48}\, 1_{21}\rangle$	1481.01	468.898	468.880	0.00
$\lvert 1_{22}\rangle \to \lvert 1_{48}\, 1_{22}\rangle$	1481.01	448.982	449.420	0.10
$\lvert 1_{23}\rangle \to \lvert 1_{48}\, 1_{23}\rangle$	1481.01	472.478	472.430	0.01
$\lvert 1_{24}\rangle \to \lvert 1_{48}\, 1_{24}\rangle$	1481.01	467.746	467.670	0.02
$\lvert 1_{25}\rangle \to \lvert 1_{48}\, 1_{25}\rangle$	1481.01	455.186	455.710	0.11
$\lvert 1_{26}\rangle \to \lvert 1_{48}\, 1_{26}\rangle$	1481.01	472.400	472.450	0.01
$\lvert 1_{27}\rangle \to \lvert 1_{48}\, 1_{27}\rangle$	1481.01	454.480	454.970	0.11
$\lvert 1_{28}\rangle \to \lvert 1_{48}\, 1_{28}\rangle$	1481.01	471.991	472.010	0.00
$\lvert 1_{29}\rangle \to \lvert 1_{48}\, 1_{29}\rangle$	1481.01	471.244	471.360	0.02
$\lvert 1_{30}\rangle \to \lvert 1_{48}\, 1_{30}\rangle$	1481.01	471.365	471.450	0.02
$\lvert 1_{31}\rangle \to \lvert 1_{48}\, 1_{31}\rangle$	1481.01	475.134	474.910	0.05
$\lvert 1_{32}\rangle \to \lvert 1_{48}\, 1_{32}\rangle$	1481.01	474.623	474.030	0.13
$\lvert 1_{33}\rangle \to \lvert 1_{48}\, 1_{33}\rangle$	1481.01	437.565	438.090	0.12
$\lvert 1_{34}\rangle \to \lvert 1_{48}\, 1_{34}\rangle$	1481.01	430.195	430.510	0.07
$\lvert 1_{35}\rangle \to \lvert 1_{48}\, 1_{35}\rangle$	1481.01	380.617	381.080	0.12
$\lvert 1_{36}\rangle \to \lvert 1_{48}\, 1_{36}\rangle$	1481.01	454.259	454.680	0.09
$\lvert 1_{37}\rangle \to \lvert 1_{48}\, 1_{37}\rangle$	1481.01	442.208	442.680	0.11
$\lvert 1_{38}\rangle \to \lvert 1_{48}\, 1_{38}\rangle$	1481.01	445.396	445.740	0.08
$\lvert 1_{39}\rangle \to \lvert 1_{48}\, 1_{39}\rangle$	1481.01	453.129	453.580	0.10
$\lvert 1_{40}\rangle \to \lvert 1_{48}\, 1_{40}\rangle$	1481.01	450.838	451.220	0.08
$\lvert 1_{41}\rangle \to \lvert 1_{48}\, 1_{41}\rangle$	1481.01	403.491	403.790	0.07
$\lvert 1_{42}\rangle \to \lvert 1_{48}\, 1_{42}\rangle$	1481.01	449.419	449.610	0.04
$\lvert 1_{43}\rangle \to \lvert 1_{48}\, 1_{43}\rangle$	1481.01	286.510	286.810	0.10
$\lvert 1_{44}\rangle \to \lvert 1_{48}\, 1_{44}\rangle$	1481.01	365.725	366.130	0.11
$\lvert 1_{45}\rangle \to \lvert 1_{48}\, 1_{45}\rangle$	1481.01	440.936	441.470	0.12
$\lvert 1_{46}\rangle \to \lvert 1_{48}\, 1_{46}\rangle$	1481.01	451.027	451.360	0.07
$\lvert 1_{47}\rangle \to \lvert 1_{48}\, 1_{47}\rangle$	1481.01	441.843	442.150	0.07
$\lvert 1_{49}\rangle \to \lvert 1_{49}\, 1_{48}\rangle$	1481.01	459.171	459.550	0.08
$\lvert 1_{50}\rangle \to \lvert 1_{50}\, 1_{48}\rangle$	1481.01	424.391	424.790	0.09
$\lvert 1_{51}\rangle \to \lvert 1_{51}\, 1_{48}\rangle$	1481.01	447.872	448.230	0.08
$\lvert 1_{52}\rangle \to \lvert 1_{52}\, 1_{48}\rangle$	1481.01	470.023	470.230	0.04

Continued on next page

Table S3: Anthracene hot-band TD/TI RR validation data grouped by transition class (continued).

Transition	ν	I^{TD}	I^{TI}	Δ
$\lvert 1_{53}\rangle \to \lvert 1_{53}\,1_{48}\rangle$	1481.01	444.737	445.030	0.07
$\lvert 1_{54}\rangle \to \lvert 1_{54}\,1_{48}\rangle$	1481.01	462.241	461.990	0.05
$\lvert 1_{56}\rangle \to \lvert 1_{56}\,1_{48}\rangle$	1481.01	453.066	453.640	0.13
$\lvert 1_{57}\rangle \to \lvert 1_{57}\,1_{48}\rangle$	1481.01	452.500	452.880	0.08
$\lvert 1_{58}\rangle \to \lvert 1_{58}\,1_{48}\rangle$	1481.01	453.234	453.590	0.08
$\lvert 1_{59}\rangle \to \lvert 1_{59}\,1_{48}\rangle$	1481.01	454.096	454.540	0.10
$\lvert 1_{60}\rangle \to \lvert 1_{60}\,1_{48}\rangle$	1481.01	454.318	454.760	0.10
$\lvert 1_{61}\rangle \to \lvert 1_{61}\,1_{48}\rangle$	1481.01	454.807	455.320	0.11
$\lvert 1_{62}\rangle \to \lvert 1_{62}\,1_{48}\rangle$	1481.01	454.211	454.580	0.08
$\lvert 1_{63}\rangle \to \lvert 1_{63}\,1_{48}\rangle$	1481.01	453.996	454.580	0.13
$\lvert 1_{64}\rangle \to \lvert 1_{64}\,1_{48}\rangle$	1481.01	450.764	451.180	0.09
$\lvert 1_{65}\rangle \to \lvert 1_{65}\,1_{48}\rangle$	1481.01	453.886	454.270	0.08
$\lvert 1_{66}\rangle \to \lvert 1_{66}\,1_{48}\rangle$	1481.01	453.619	453.980	0.08
		$1_i \to 1_i 2_j$		
$\lvert 1_{1}\rangle \to \lvert 2_{13}\,1_{1}\rangle$	1204.48	34.304	34.208	0.28
$\lvert 1_{2}\rangle \to \lvert 2_{13}\,1_{2}\rangle$	1204.48	34.309	34.151	0.46
$\lvert 1_{3}\rangle \to \lvert 2_{13}\,1_{3}\rangle$	1204.48	34.899	34.694	0.59
$\lvert 1_{4}\rangle \to \lvert 2_{13}\,1_{4}\rangle$	1204.48	33.724	33.633	0.27
$\lvert 1_{5}\rangle \to \lvert 2_{13}\,1_{5}\rangle$	1204.48	34.218	34.054	0.48
$\lvert 1_{6}\rangle \to \lvert 2_{13}\,1_{6}\rangle$	1204.48	35.431	35.115	0.90
$\lvert 1_{7}\rangle \to \lvert 2_{13}\,1_{7}\rangle$	1204.48	34.205	33.926	0.82
$\lvert 1_{9}\rangle \to \lvert 2_{13}\,1_{9}\rangle$	1204.48	35.409	34.897	1.47
$\lvert 1_{10}\rangle \to \lvert 2_{13}\,1_{10}\rangle$	1204.48	35.245	34.834	1.18
$\lvert 1_{11}\rangle \to \lvert 2_{13}\,1_{11}\rangle$	1204.48	35.783	35.015	2.19
$\lvert 1_{12}\rangle \to \lvert 2_{13}\,1_{12}\rangle$	1204.48	34.279	33.960	0.94
$\lvert 1_{14}\rangle \to \lvert 1_{14}\,2_{13}\rangle$	1204.48	33.675	33.411	0.79
$\lvert 1_{15}\rangle \to \lvert 1_{15}\,2_{13}\rangle$	1204.48	34.342	34.287	0.16
$\lvert 1_{16}\rangle \to \lvert 1_{16}\,2_{13}\rangle$	1204.48	32.161	32.022	0.43
$\lvert 1_{17}\rangle \to \lvert 1_{17}\,2_{13}\rangle$	1204.48	34.970	34.570	1.16
$\lvert 1_{18}\rangle \to \lvert 1_{18}\,2_{13}\rangle$	1204.48	36.160	35.499	1.86
$\lvert 1_{20}\rangle \to \lvert 1_{20}\,2_{13}\rangle$	1204.48	35.524	35.043	1.37
$\lvert 1_{21}\rangle \to \lvert 1_{21}\,2_{13}\rangle$	1204.48	35.121	34.627	1.43
$\lvert 1_{22}\rangle \to \lvert 1_{22}\,2_{13}\rangle$	1204.48	33.148	32.855	0.89
$\lvert 1_{23}\rangle \to \lvert 1_{23}\,2_{13}\rangle$	1204.48	36.036	35.527	1.43
$\lvert 1_{24}\rangle \to \lvert 1_{24}\,2_{13}\rangle$	1204.48	35.498	34.983	1.47

Continued on next page

Table S3: Anthracene hot-band TD/TI RR validation data grouped by transition class (continued).

Transition	ν	I^{TD}	I^{TI}	Δ
$\|1_{25}\rangle \rightarrow \|1_{25}\,2_{13}\rangle$	1204.48	33.643	33.376	0.80
$\|1_{26}\rangle \rightarrow \|1_{26}\,2_{13}\rangle$	1204.48	35.925	35.388	1.52
$\|1_{27}\rangle \rightarrow \|1_{27}\,2_{13}\rangle$	1204.48	33.672	33.346	0.98
$\|1_{28}\rangle \rightarrow \|1_{28}\,2_{13}\rangle$	1204.48	35.880	35.404	1.34
$\|1_{29}\rangle \rightarrow \|1_{29}\,2_{13}\rangle$	1204.48	35.746	35.256	1.39
$\|1_{30}\rangle \rightarrow \|1_{30}\,2_{13}\rangle$	1204.48	35.754	35.238	1.47
$\|1_{31}\rangle \rightarrow \|1_{31}\,2_{13}\rangle$	1204.48	36.402	35.855	1.53
$\|1_{32}\rangle \rightarrow \|1_{32}\,2_{13}\rangle$	1204.48	36.239	35.609	1.77
$\|1_{33}\rangle \rightarrow \|1_{33}\,2_{13}\rangle$	1204.48	31.932	31.723	0.66
$\|1_{34}\rangle \rightarrow \|1_{34}\,2_{13}\rangle$	1204.48	30.809	30.767	0.14
$\|1_{36}\rangle \rightarrow \|1_{36}\,2_{13}\rangle$	1204.48	33.594	33.272	0.97
$\|1_{37}\rangle \rightarrow \|1_{37}\,2_{13}\rangle$	1204.48	32.384	32.167	0.68
$\|1_{38}\rangle \rightarrow \|1_{38}\,2_{13}\rangle$	1204.48	32.705	32.660	0.14
$\|1_{39}\rangle \rightarrow \|1_{39}\,2_{13}\rangle$	1204.48	33.388	33.124	0.80
$\|1_{40}\rangle \rightarrow \|1_{40}\,2_{13}\rangle$	1204.48	33.255	32.927	1.00
$\|1_{41}\rangle \rightarrow \|1_{41}\,2_{13}\rangle$	1204.48	28.985	28.941	0.15
$\|1_{42}\rangle \rightarrow \|1_{42}\,2_{13}\rangle$	1204.48	33.439	33.331	0.33
$\|1_{45}\rangle \rightarrow \|1_{45}\,2_{13}\rangle$	1204.48	32.408	32.147	0.81
$\|1_{46}\rangle \rightarrow \|1_{46}\,2_{13}\rangle$	1204.48	33.276	32.886	1.19
$\|1_{47}\rangle \rightarrow \|1_{47}\,2_{13}\rangle$	1204.48	32.100	32.034	0.21
$\|1_{49}\rangle \rightarrow \|1_{49}\,2_{13}\rangle$	1204.48	34.053	33.950	0.30
$\|1_{50}\rangle \rightarrow \|1_{50}\,2_{13}\rangle$	1204.48	30.966	30.721	0.80
$\|1_{51}\rangle \rightarrow \|1_{51}\,2_{13}\rangle$	1204.48	33.524	33.474	0.15
$\|1_{52}\rangle \rightarrow \|1_{52}\,2_{13}\rangle$	1204.48	35.493	35.035	1.31
$\|1_{53}\rangle \rightarrow \|1_{53}\,2_{13}\rangle$	1204.48	32.430	32.390	0.12
$\|1_{54}\rangle \rightarrow \|1_{54}\,2_{13}\rangle$	1204.48	34.721	33.863	2.53
$\|1_{56}\rangle \rightarrow \|1_{56}\,2_{13}\rangle$	1204.48	33.368	33.116	0.76
$\|1_{57}\rangle \rightarrow \|1_{57}\,2_{13}\rangle$	1204.48	33.243	33.118	0.38
$\|1_{58}\rangle \rightarrow \|1_{58}\,2_{13}\rangle$	1204.48	33.347	33.298	0.15
$\|1_{59}\rangle \rightarrow \|1_{59}\,2_{13}\rangle$	1204.48	33.440	33.226	0.64
$\|1_{60}\rangle \rightarrow \|1_{60}\,2_{13}\rangle$	1204.48	33.465	33.212	0.76
$\|1_{61}\rangle \rightarrow \|1_{61}\,2_{13}\rangle$	1204.48	33.525	33.307	0.66
$\|1_{62}\rangle \rightarrow \|1_{62}\,2_{13}\rangle$	1204.48	33.454	33.398	0.17
$\|1_{63}\rangle \rightarrow \|1_{63}\,2_{13}\rangle$	1204.48	33.432	33.194	0.72
$\|1_{64}\rangle \rightarrow \|1_{64}\,2_{13}\rangle$	1204.48	33.009	32.855	0.47
$\|1_{65}\rangle \rightarrow \|1_{65}\,2_{13}\rangle$	1204.48	33.416	33.330	0.26

Continued on next page

Table S3: Anthracene hot-band TD/TI RR validation data grouped by transition class (continued).

Transition	ν	I^{TD}	I^{TI}	Δ
$\|1_{66}\rangle \rightarrow \|1_{66}\,2_{13}\rangle$	1204.48	33.394	33.347	0.14
$\|1_{1}\rangle \rightarrow \|2_{48}\,1_{1}\rangle$	2962.02	28.384	28.350	0.12
$\|1_{2}\rangle \rightarrow \|2_{48}\,1_{2}\rangle$	2962.02	28.495	28.465	0.10
$\|1_{3}\rangle \rightarrow \|2_{48}\,1_{3}\rangle$	2962.02	29.144	29.077	0.23
$\|1_{4}\rangle \rightarrow \|2_{48}\,1_{4}\rangle$	2962.02	27.570	27.617	0.17
$\|1_{5}\rangle \rightarrow \|2_{48}\,1_{5}\rangle$	2962.02	28.820	28.659	0.56
$\|1_{6}\rangle \rightarrow \|2_{48}\,1_{6}\rangle$	2962.02	29.822	29.706	0.39
$\|1_{7}\rangle \rightarrow \|2_{48}\,1_{7}\rangle$	2962.02	28.079	28.079	0.00
$\|1_{9}\rangle \rightarrow \|2_{48}\,1_{9}\rangle$	2962.02	29.632	29.661	0.10
$\|1_{10}\rangle \rightarrow \|2_{48}\,1_{10}\rangle$	2962.02	29.480	29.299	0.62
$\|1_{11}\rangle \rightarrow \|2_{48}\,1_{11}\rangle$	2962.02	30.472	30.456	0.05
$\|1_{12}\rangle \rightarrow \|2_{48}\,1_{12}\rangle$	2962.02	28.155	28.134	0.08
$\|1_{13}\rangle \rightarrow \|2_{48}\,1_{13}\rangle$	2962.02	30.854	30.586	0.88
$\|1_{14}\rangle \rightarrow \|2_{48}\,1_{14}\rangle$	2962.02	27.554	27.567	0.05
$\|1_{15}\rangle \rightarrow \|2_{48}\,1_{15}\rangle$	2962.02	28.214	28.217	0.01
$\|1_{17}\rangle \rightarrow \|2_{48}\,1_{17}\rangle$	2962.02	28.972	28.881	0.31
$\|1_{18}\rangle \rightarrow \|2_{48}\,1_{18}\rangle$	2962.02	30.537	30.294	0.80
$\|1_{20}\rangle \rightarrow \|2_{48}\,1_{20}\rangle$	2962.02	29.945	29.742	0.68
$\|1_{21}\rangle \rightarrow \|2_{48}\,1_{21}\rangle$	2962.02	29.874	29.683	0.64
$\|1_{23}\rangle \rightarrow \|2_{48}\,1_{23}\rangle$	2962.02	30.163	29.965	0.66
$\|1_{24}\rangle \rightarrow \|2_{48}\,1_{24}\rangle$	2962.02	29.877	29.693	0.62
$\|1_{25}\rangle \rightarrow \|2_{48}\,1_{25}\rangle$	2962.02	27.544	27.555	0.04
$\|1_{26}\rangle \rightarrow \|2_{48}\,1_{26}\rangle$	2962.02	29.984	29.806	0.60
$\|1_{27}\rangle \rightarrow \|2_{48}\,1_{27}\rangle$	2962.02	27.696	27.659	0.14
$\|1_{28}\rangle \rightarrow \|2_{48}\,1_{28}\rangle$	2962.02	29.941	29.802	0.47
$\|1_{29}\rangle \rightarrow \|2_{48}\,1_{29}\rangle$	2962.02	29.832	29.668	0.55
$\|1_{30}\rangle \rightarrow \|2_{48}\,1_{30}\rangle$	2962.02	29.818	29.690	0.43
$\|1_{31}\rangle \rightarrow \|2_{48}\,1_{31}\rangle$	2962.02	30.625	30.438	0.61
$\|1_{32}\rangle \rightarrow \|2_{48}\,1_{32}\rangle$	2962.02	30.574	30.565	0.03
$\|1_{36}\rangle \rightarrow \|2_{48}\,1_{36}\rangle$	2962.02	27.621	27.614	0.03
$\|1_{39}\rangle \rightarrow \|2_{48}\,1_{39}\rangle$	2962.02	27.296	27.337	0.15
$\|1_{40}\rangle \rightarrow \|2_{48}\,1_{40}\rangle$	2962.02	27.419	27.411	0.03
$\|1_{42}\rangle \rightarrow \|2_{48}\,1_{42}\rangle$	2962.02	28.121	28.063	0.21
$\|1_{46}\rangle \rightarrow \|2_{48}\,1_{46}\rangle$	2962.02	27.669	27.628	0.15
$\|1_{49}\rangle \rightarrow \|1_{49}\,2_{48}\rangle$	2962.02	27.947	27.971	0.09
$\|1_{52}\rangle \rightarrow \|1_{52}\,2_{48}\rangle$	2962.02	29.502	29.386	0.40

Continued on next page

Table S3: Anthracene hot-band TD/TI RR validation data grouped by transition class (continued).

Transition	ν	I^{TD}	I^{TI}	Δ
$\vert 1_{54}\rangle \to \vert 1_{54}\,2_{48}\rangle$	2962.02	30.420	30.234	0.61
$\vert 1_{59}\rangle \to \vert 1_{59}\,2_{48}\rangle$	2962.02	27.277	27.323	0.17
$\vert 1_{60}\rangle \to \vert 1_{60}\,2_{48}\rangle$	2962.02	27.305	27.348	0.16
$\vert 1_{61}\rangle \to \vert 1_{61}\,2_{48}\rangle$	2962.02	27.361	27.402	0.15
$\vert 1_{62}\rangle \to \vert 1_{62}\,2_{48}\rangle$	2962.02	27.291	27.349	0.21
$\vert 1_{63}\rangle \to \vert 1_{63}\,2_{48}\rangle$	2962.02	27.267	27.298	0.11
$\vert 1_{65}\rangle \to \vert 1_{65}\,2_{48}\rangle$	2962.02	27.253	27.313	0.22
$1_i \to 2_i 1_j$				
$\vert 1_{1}\rangle \to \vert 1_{6}\,2_{1}\rangle$	485.60	2.167	2.149	0.80
$\vert 1_{6}\rangle \to \vert 2_{6}\,1_{1}\rangle$	485.60	2.135	2.031	5.14
$\vert 1_{1}\rangle \to \vert 1_{9}\,2_{1}\rangle$	581.90	4.025	4.005	0.49
$\vert 1_{9}\rangle \to \vert 2_{9}\,1_{1}\rangle$	581.90	3.969	3.865	2.71
$\vert 1_{2}\rangle \to \vert 1_{11}\,2_{2}\rangle$	638.41	8.117	8.071	0.57
$\vert 1_{11}\rangle \to \vert 2_{11}\,1_{2}\rangle$	638.41	7.898	7.505	5.24
$\vert 1_{5}\rangle \to \vert 1_{13}\,2_{5}\rangle$	876.50	21.935	21.862	0.33
$\vert 1_{13}\rangle \to \vert 2_{13}\,1_{5}\rangle$	876.50	19.940	19.140	4.18
$\vert 1_{6}\rangle \to \vert 1_{9}\,2_{6}\rangle$	884.33	3.860	3.880	0.52
$\vert 1_{9}\rangle \to \vert 2_{9}\,1_{6}\rangle$	884.33	3.608	3.522	2.46
$\vert 1_{2}\rangle \to \vert 1_{18}\,2_{2}\rangle$	906.10	1.127	1.123	0.37
$\vert 1_{18}\rangle \to \vert 2_{18}\,1_{2}\rangle$	906.10	1.106	1.074	2.98
$\vert 1_{3}\rangle \to \vert 1_{20}\,2_{3}\rangle$	1023.76	3.264	3.259	0.16
$\vert 1_{20}\rangle \to \vert 2_{20}\,1_{3}\rangle$	1023.76	2.956	2.883	2.53
$\vert 1_{5}\rangle \to \vert 1_{21}\,2_{5}\rangle$	1081.69	4.023	4.001	0.57
$\vert 1_{21}\rangle \to \vert 2_{21}\,1_{5}\rangle$	1081.69	3.767	3.672	2.60
$\vert 1_{8}\rangle \to \vert 1_{19}\,2_{8}\rangle$	1186.35	28.666	28.679	0.04
$\vert 1_{19}\rangle \to \vert 2_{19}\,1_{8}\rangle$	1186.35	21.980	21.991	0.05
$\vert 1_{10}\rangle \to \vert 1_{20}\,2_{10}\rangle$	1283.68	6.177	6.145	0.52
$\vert 1_{20}\rangle \to \vert 2_{20}\,1_{10}\rangle$	1283.68	5.629	5.550	1.42
$\vert 1_{11}\rangle \to \vert 1_{24}\,2_{11}\rangle$	1402.81	3.085	3.068	0.55
$\vert 1_{24}\rangle \to \vert 2_{24}\,1_{11}\rangle$	1402.81	2.568	2.534	1.32
$\vert 1_{13}\rangle \to \vert 1_{21}\,2_{13}\rangle$	1409.67	2.392	2.353	1.68
$\vert 1_{21}\rangle \to \vert 2_{21}\,1_{13}\rangle$	1409.67	2.029	1.980	2.46
$\vert 1_{8}\rangle \to \vert 1_{34}\,2_{8}\rangle$	1442.96	3.333	3.337	0.11
$\vert 1_{34}\rangle \to \vert 2_{34}\,1_{8}\rangle$	1442.96	3.798	3.797	0.02
$\vert 1_{38}\rangle \to \vert 2_{38}\,1_{8}\rangle$	1596.72	1.024	1.025	0.13

Continued on next page

Table S3: Anthracene hot-band TD/TI RR validation data grouped by transition class (continued).

Transition	ν	I^{TD}	I^{TI}	Δ
$\|1_{18}\rangle \to \|1_{24}\,2_{18}\rangle$	1670.50	3.332	3.333	0.02
$\|1_{24}\rangle \to \|2_{24}\,1_{18}\rangle$	1670.50	3.086	3.064	0.69
$\|1_{8}\rangle \to \|1_{43}\,2_{8}\rangle$	1707.38	20.790	20.805	0.07
$\|1_{43}\rangle \to \|2_{43}\,1_{8}\rangle$	1707.38	18.549	18.566	0.09
$\|1_{20}\rangle \to \|1_{29}\,2_{20}\rangle$	1789.73	1.314	1.309	0.36
$\|1_{29}\rangle \to \|2_{29}\,1_{20}\rangle$	1789.73	1.203	1.199	0.28
$\|1_{19}\rangle \to \|1_{34}\,2_{19}\rangle$	1824.86	4.615	4.625	0.21
$\|1_{34}\rangle \to \|2_{34}\,1_{19}\rangle$	1824.86	6.455	6.463	0.13
$\|1_{8}\rangle \to \|1_{48}\,2_{8}\rangle$	1883.24	36.818	36.841	0.06
$\|1_{48}\rangle \to \|2_{48}\,1_{8}\rangle$	1883.24	23.322	23.325	0.01
$\|1_{8}\rangle \to \|1_{51}\,2_{8}\rangle$	1942.31	1.580	1.581	0.04
$\|1_{51}\rangle \to \|2_{51}\,1_{8}\rangle$	1942.31	1.934	1.942	0.40
$\|1_{22}\rangle \to \|1_{35}\,2_{22}\rangle$	1963.87	2.801	2.803	0.08
$\|1_{35}\rangle \to \|2_{35}\,1_{22}\rangle$	1963.87	2.407	2.423	0.67
$\|1_{16}\rangle \to \|1_{41}\,2_{16}\rangle$	1967.98	2.543	2.545	0.08
$\|1_{41}\rangle \to \|2_{41}\,1_{16}\rangle$	1967.98	2.402	2.405	0.13
$\|1_{19}\rangle \to \|1_{38}\,2_{19}\rangle$	1978.62	1.426	1.426	0.05
$\|1_{38}\rangle \to \|2_{38}\,1_{19}\rangle$	1978.62	1.928	1.929	0.09
$\|1_{19}\rangle \to \|1_{43}\,2_{19}\rangle$	2089.28	30.087	30.095	0.03
$\|1_{43}\rangle \to \|2_{43}\,1_{19}\rangle$	2089.28	30.399	30.412	0.04
$\|1_{33}\rangle \to \|1_{35}\,2_{33}\rangle$	2160.56	2.775	2.780	0.16
$\|1_{35}\rangle \to \|2_{35}\,1_{33}\rangle$	2160.56	2.420	2.428	0.32
$\|1_{16}\rangle \to \|1_{50}\,2_{16}\rangle$	2178.59	1.225	1.224	0.08
$\|1_{50}\rangle \to \|2_{50}\,1_{16}\rangle$	2178.59	1.203	1.204	0.07
$\|1_{22}\rangle \to \|1_{44}\,2_{22}\rangle$	2191.99	2.346	2.345	0.03
$\|1_{44}\rangle \to \|2_{44}\,1_{22}\rangle$	2191.99	2.003	2.019	0.78
$\|1_{19}\rangle \to \|1_{48}\,2_{19}\rangle$	2265.13	55.765	55.900	0.24
$\|1_{48}\rangle \to \|2_{48}\,1_{19}\rangle$	2265.13	39.286	39.335	0.12
$\|1_{19}\rangle \to \|1_{51}\,2_{19}\rangle$	2324.21	2.269	2.272	0.13
$\|1_{51}\rangle \to \|2_{51}\,1_{19}\rangle$	2324.21	3.213	3.224	0.33
$\|1_{34}\rangle \to \|1_{43}\,2_{34}\rangle$	2345.89	6.641	6.633	0.12
$\|1_{43}\rangle \to \|2_{43}\,1_{34}\rangle$	2345.89	5.081	5.076	0.10
$\|1_{33}\rangle \to \|1_{44}\,2_{33}\rangle$	2388.68	1.560	1.565	0.28
$\|1_{44}\rangle \to \|2_{44}\,1_{33}\rangle$	2388.68	1.388	1.391	0.22
$\|1_{16}\rangle \to \|1_{55}\,2_{16}\rangle$	2393.05	13.080	13.064	0.12
$\|1_{55}\rangle \to \|2_{55}\,1_{16}\rangle$	2393.05	4.321	4.376	1.26

Continued on next page

Table S3: Anthracene hot-band TD/TI RR validation data grouped by transition class (continued).

Transition	ν	I^{TD}	I^{TI}	Δ
$\lvert 1_{35}\rangle \to \lvert 1_{44}\,2_{35}\rangle$	2489.71	5.286	5.303	0.32
$\lvert 1_{44}\rangle \to \lvert 2_{44}\,1_{35}\rangle$	2489.71	5.709	5.722	0.23
$\lvert 1_{38}\rangle \to \lvert 1_{43}\,2_{38}\rangle$	2499.66	1.125	1.125	0.03
$\lvert 1_{19}\rangle \to \lvert 1_{56}\,2_{19}\rangle$	2519.43	1.597	1.598	0.08
$\lvert 1_{56}\rangle \to \lvert 2_{56}\,1_{19}\rangle$	2519.43	2.139	2.137	0.09
$\lvert 1_{34}\rangle \to \lvert 1_{48}\,2_{34}\rangle$	2521.75	12.880	12.854	0.20
$\lvert 1_{48}\rangle \to \lvert 2_{48}\,1_{34}\rangle$	2521.75	6.918	6.921	0.03
$\lvert 1_{27}\rangle \to \lvert 1_{54}\,2_{27}\rangle$	2610.12	1.056	1.057	0.10
$\lvert 1_{25}\rangle \to \lvert 1_{55}\,2_{25}\rangle$	2653.81	1.167	1.169	0.19
$\lvert 1_{38}\rangle \to \lvert 1_{48}\,2_{38}\rangle$	2675.51	2.447	2.452	0.21
$\lvert 1_{48}\rangle \to \lvert 2_{48}\,1_{38}\rangle$	2675.51	1.220	1.222	0.17
$\lvert 1_{43}\rangle \to \lvert 1_{48}\,2_{43}\rangle$	2786.17	36.215	36.169	0.13
$\lvert 1_{48}\rangle \to \lvert 2_{48}\,1_{43}\rangle$	2786.17	23.926	23.943	0.07
$\lvert 1_{41}\rangle \to \lvert 1_{50}\,2_{41}\rangle$	2812.73	1.184	1.186	0.17
$\lvert 1_{50}\rangle \to \lvert 2_{50}\,1_{41}\rangle$	2812.73	1.292	1.294	0.17
$\lvert 1_{43}\rangle \to \lvert 1_{51}\,2_{43}\rangle$	2845.24	3.662	3.661	0.02
$\lvert 1_{51}\rangle \to \lvert 2_{51}\,1_{43}\rangle$	2845.24	4.516	4.515	0.02
$\lvert 1_{40}\rangle \to \lvert 1_{54}\,2_{40}\rangle$	2890.58	1.293	1.293	0.02
$\lvert 1_{54}\rangle \to \lvert 2_{54}\,1_{40}\rangle$	2890.58	1.215	1.210	0.39
$\lvert 1_{37}\rangle \to \lvert 1_{55}\,2_{37}\rangle$	2911.81	4.350	4.351	0.01
$\lvert 1_{55}\rangle \to \lvert 2_{55}\,1_{37}\rangle$	2911.81	1.552	1.558	0.38
$\lvert 1_{42}\rangle \to \lvert 1_{54}\,2_{42}\rangle$	2977.95	3.590	3.589	0.02
$\lvert 1_{54}\rangle \to \lvert 2_{54}\,1_{42}\rangle$	2977.95	3.315	3.304	0.35
$\lvert 1_{48}\rangle \to \lvert 1_{51}\,2_{48}\rangle$	3021.10	2.050	2.061	0.53
$\lvert 1_{51}\rangle \to \lvert 2_{51}\,1_{48}\rangle$	3021.10	4.290	4.291	0.03
$\lvert 1_{41}\rangle \to \lvert 1_{55}\,2_{41}\rangle$	3027.19	14.902	14.926	0.16
$\lvert 1_{55}\rangle \to \lvert 2_{55}\,1_{41}\rangle$	3027.19	6.016	6.029	0.22
$\lvert 1_{43}\rangle \to \lvert 1_{56}\,2_{43}\rangle$	3040.46	1.415	1.417	0.12
$\lvert 1_{56}\rangle \to \lvert 2_{56}\,1_{43}\rangle$	3040.46	1.734	1.738	0.23
$\lvert 1_{45}\rangle \to \lvert 1_{55}\,2_{45}\rangle$	3090.37	4.906	4.906	0.01
$\lvert 1_{55}\rangle \to \lvert 2_{55}\,1_{45}\rangle$	3090.37	1.708	1.707	0.01
$\lvert 1_{46}\rangle \to \lvert 1_{54}\,2_{46}\rangle$	3099.11	1.787	1.793	0.36
$\lvert 1_{54}\rangle \to \lvert 2_{54}\,1_{46}\rangle$	3099.11	1.695	1.693	0.10
$\lvert 1_{48}\rangle \to \lvert 1_{56}\,2_{48}\rangle$	3216.31	2.161	2.163	0.09
$\lvert 1_{56}\rangle \to \lvert 2_{56}\,1_{48}\rangle$	3216.31	3.250	3.253	0.09
$\lvert 1_{50}\rangle \to \lvert 1_{55}\,2_{50}\rangle$	3237.80	7.421	7.413	0.10

Continued on next page

Table S3: Anthracene hot-band TD/TI RR validation data grouped by transition class (continued).

Transition	ν	I^{TD}	I^{TI}	Δ
$\lvert 1_{55}\rangle \to \lvert 2_{55}\,1_{50}\rangle$	3237.80	2.524	2.516	0.33

1.6 Tighter-prescreening test for anthracene hot bands

Transitions whose default-prescreening TD/TI relative difference was larger than 1% were recomputed with increasingly tight TI prescreening settings. The L, XL, and XXL levels use $C_1^{\max} = 40$ and $C_2^{\max} = 30$, with effective maximum integral counts of 10^9, 2×10^9, and 4×10^9, respectively. The transition-resolved comparison is reported in Table S4. For the nine transitions recomputed at the XL level, the mean relative difference decreased from 3.05% with the default prescreening to 0.95%; three transitions remained above 1% and were selected for the XXL calculation.

Table S4: Anthracene hot-band transitions with default-prescreening TD/TI relative errors larger than 1%, recomputed with increasingly tight TI prescreening. The L, XL, and XXL levels use $C_1^{\max} = 40$ and $C_2^{\max} = 30$, with effective maximum integral counts of 10^9, 2×10^9, and 4×10^9, respectively. Intensities are reported in units of 10^{-1} cm^3 mol^{-1} sr^{-1}; relative differences are reported in percent.

Transition	I^{TD}	$I^{\mathrm{TI,def}}$	Δ^{def}	$I^{\mathrm{TI,L}}$	Δ^{L}	$I^{\mathrm{TI,XL}}$	Δ^{XL}	$I^{\mathrm{TI,XXL}}$	Δ^{XXL}
				$1_i \to 2_i$					
$\lvert 1_{62}\rangle \to \lvert 2_{62}\rangle$	0.008	0.008	1.91	0.008	0.24	–	–	–	–
				$1_i \to 3_i$					
$\lvert 1_{56}\rangle \to \lvert 3_{56}\rangle$	0.080	0.079	1.16	0.080	0.56	0.080	0.48	–	–
				$1_i \to 1_i 1_j$					
$\lvert 1_{11}\rangle \to \lvert 1_{19}\,1_{11}\rangle$	303.195	299.450	1.25	301.740	0.48	–	–	–	–
				$1_i \to 1_i 2_j$					
$\lvert 1_{9}\rangle \to \lvert 2_{13}\,1_{9}\rangle$	35.409	34.897	1.47	35.116	0.83	–	–	–	–
$\lvert 1_{10}\rangle \to \lvert 2_{13}\,1_{10}\rangle$	35.245	34.834	1.18	35.147	0.28	–	–	–	–
$\lvert 1_{11}\rangle \to \lvert 2_{13}\,1_{11}\rangle$	35.783	35.015	2.19	35.362	1.19	35.374	1.16	35.615	0.47

Continued on next page

Table S4: Anthracene hot-band precision-progression comparison (continued).

Transition	I^{TD}	$I^{\mathrm{TI,def}}$	Δ^{def}	$I^{\mathrm{TI,L}}$	Δ^{L}	$I^{\mathrm{TI,XL}}$	Δ^{XL}	$I^{\mathrm{TI,XXL}}$	Δ^{XXL}
$\vert 1_{17}\rangle \to \vert 1_{17}\, 2_{13}\rangle$	34.970	34.570	1.16	34.782	0.54	–	–	–	–
$\vert 1_{18}\rangle \to \vert 1_{18}\, 2_{13}\rangle$	36.160	35.499	1.86	35.935	0.62	–	–	–	–
$\vert 1_{20}\rangle \to \vert 1_{20}\, 2_{13}\rangle$	35.524	35.043	1.37	35.340	0.52	–	–	–	–
$\vert 1_{21}\rangle \to \vert 1_{21}\, 2_{13}\rangle$	35.121	34.627	1.43	34.953	0.48	–	–	–	–
$\vert 1_{23}\rangle \to \vert 1_{23}\, 2_{13}\rangle$	36.036	35.527	1.43	35.846	0.53	–	–	–	–
$\vert 1_{24}\rangle \to \vert 1_{24}\, 2_{13}\rangle$	35.498	34.983	1.47	35.286	0.60	–	–	–	–
$\vert 1_{26}\rangle \to \vert 1_{26}\, 2_{13}\rangle$	35.925	35.388	1.52	35.715	0.59	–	–	–	–
$\vert 1_{28}\rangle \to \vert 1_{28}\, 2_{13}\rangle$	35.880	35.404	1.34	35.508	1.05	35.733	0.41	–	–
$\vert 1_{29}\rangle \to \vert 1_{29}\, 2_{13}\rangle$	35.746	35.256	1.39	35.552	0.55	–	–	–	–
$\vert 1_{30}\rangle \to \vert 1_{30}\, 2_{13}\rangle$	35.754	35.238	1.47	35.572	0.51	–	–	–	–
$\vert 1_{31}\rangle \to \vert 1_{31}\, 2_{13}\rangle$	36.402	35.855	1.53	36.122	0.78	–	–	–	–
$\vert 1_{32}\rangle \to \vert 1_{32}\, 2_{13}\rangle$	36.239	35.609	1.77	35.902	0.94	–	–	–	–
$\vert 1_{46}\rangle \to \vert 1_{46}\, 2_{13}\rangle$	33.276	32.886	1.19	33.106	0.51	–	–	–	–
$\vert 1_{52}\rangle \to \vert 1_{52}\, 2_{13}\rangle$	35.493	35.035	1.31	35.315	0.50	–	–	–	–
$\vert 1_{54}\rangle \to \vert 1_{54}\, 2_{13}\rangle$	34.721	33.863	2.53	34.526	0.57	–	–	–	–
$1_i \to 2_i 1_j$									
$\vert 1_{6}\rangle \to \vert 2_{6}\, 1_{1}\rangle$	2.135	2.031	5.14	2.102	1.58	2.112	1.08	2.118	0.81
$\vert 1_{9}\rangle \to \vert 2_{9}\, 1_{1}\rangle$	3.969	3.865	2.71	3.918	1.30	3.948	0.54	–	–
$\vert 1_{11}\rangle \to \vert 2_{11}\, 1_{2}\rangle$	7.898	7.505	5.24	7.694	2.66	7.701	2.56	7.830	0.87
$\vert 1_{13}\rangle \to \vert 2_{13}\, 1_{5}\rangle$	19.940	19.140	4.18	19.673	1.36	19.753	0.95	–	–
$\vert 1_{9}\rangle \to \vert 2_{9}\, 1_{6}\rangle$	3.608	3.522	2.46	3.559	1.40	3.582	0.74	–	–
$\vert 1_{18}\rangle \to \vert 2_{18}\, 1_{2}\rangle$	1.106	1.074	2.98	1.094	1.07	1.099	0.66	–	–
$\vert 1_{20}\rangle \to \vert 2_{20}\, 1_{3}\rangle$	2.956	2.883	2.53	2.930	0.89	–	–	–	–
$\vert 1_{21}\rangle \to \vert 2_{21}\, 1_{5}\rangle$	3.767	3.672	2.60	3.735	0.87	–	–	–	–
$\vert 1_{20}\rangle \to \vert 2_{20}\, 1_{10}\rangle$	5.629	5.550	1.42	5.597	0.58	–	–	–	–
$\vert 1_{24}\rangle \to \vert 2_{24}\, 1_{11}\rangle$	2.568	2.534	1.32	2.552	0.60	–	–	–	–
$\vert 1_{13}\rangle \to \vert 1_{21}\, 2_{13}\rangle$	2.392	2.353	1.68	2.378	0.61	–	–	–	–
$\vert 1_{21}\rangle \to \vert 2_{21}\, 1_{13}\rangle$	2.029	1.980	2.46	2.012	0.85	–	–	–	–
$\vert 1_{55}\rangle \to \vert 2_{55}\, 1_{16}\rangle$	4.321	4.376	1.26	4.351	0.69	–	–	–	–

2 Additional data for anisole

Anisole was used as an additional TD/TI validation case within the adiabatic Hessian model. The Duschinsky transformation associated with the $S_0 \to S_1$ transition is shown in Figure S5.

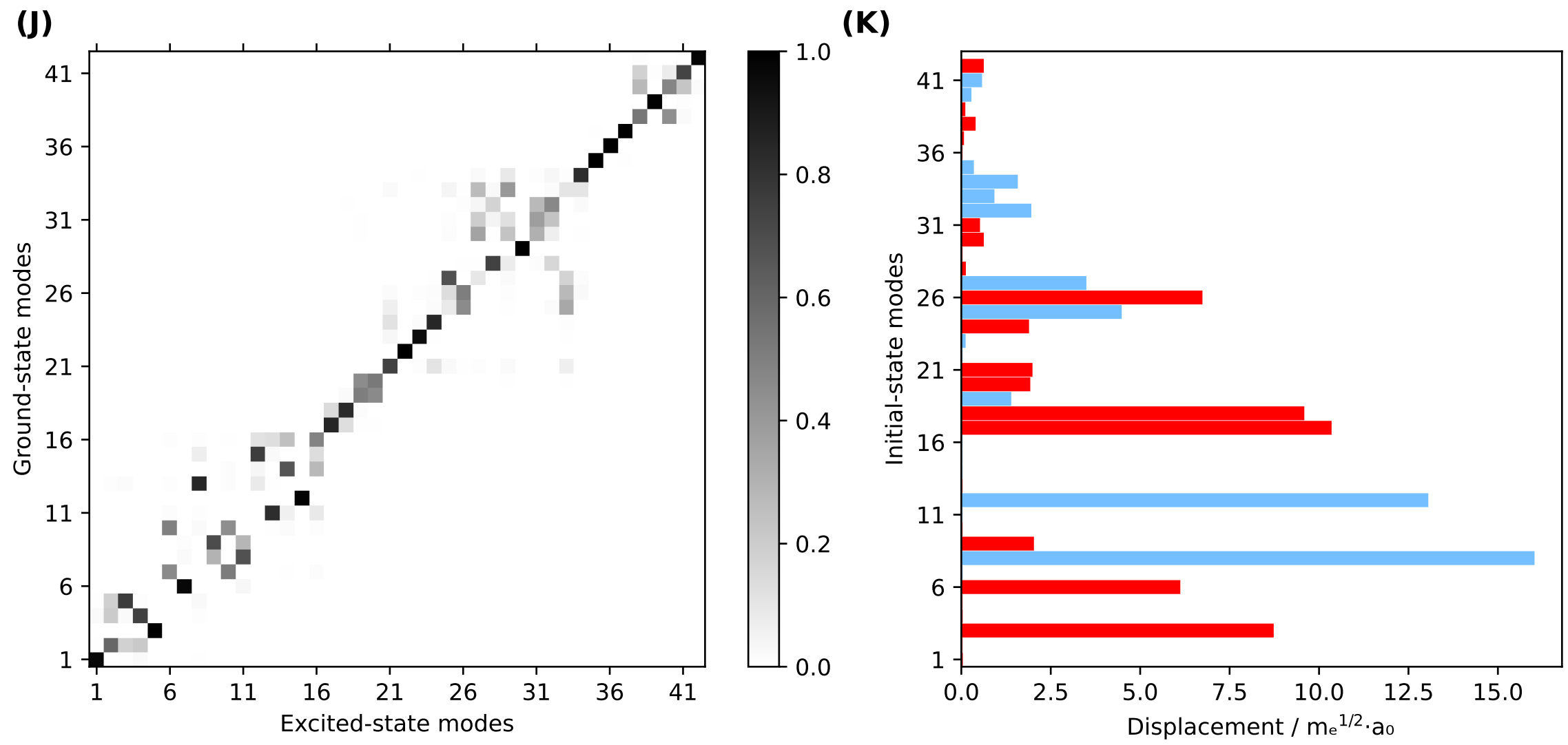

Figure S5: Duschinsky matrix (**J**, left panel) and shift vector (**K**, right panel) associated to the $S_0 \rightarrow S_1$ transition of anisole within the adiabatic Hessian model. Each matrix element has been squared and a shade of gray assigned based on the value (1: black, 0: white). The shift vector is reported in atomic units. Red bars indicate negative values, blue for positive ones.

The full default-intense anisole OPA/RR validation set is reported in Table S5 and grouped by transition class. The table contains indexed transitions with $I^{\mathrm{TD}} > 10^{-4}$ cm^3 mol^{-1} sr^{-1}, retaining at most 100 transitions per class. The included classes comprise fundamentals, overtones, ground-state combination bands, and the hot-band/progression classes $1_i \rightarrow 2_i$, $1_i \rightarrow 3_i$, $1_i \rightarrow 1_i 1_j$, $1_i \rightarrow 2_i 1_j$, and $1_i \rightarrow 1_i 2_j$. For each transition, the table reports the TD and default-prescreening TI RR intensities, the endpoint OPA sum-rule convergences η_i^{def} and η_f^{def}, and the Duschinsky mode-purity diagnostic $\max_k((\mathsf{J}_{ik})^2)$.

Table S5: Full anisole default-intense OPA/RR validation data grouped by transition class. RR intensities are from the default-prescreening TI calculation and the corresponding TD calculation for indexed transitions with $I^{\mathrm{TD}} > 10^{-4}$ cm^3 mol^{-1} sr^{-1}, retaining at most 100 transitions per class. Intensities are reported in units of 10^{-4} cm^3 mol^{-1} sr^{-1}. OPA convergence values, denoted η, are the state sum-rule convergences for the initial and final vibrational states; the smaller endpoint value is colored red. Relative differences are reported in percent. Asterisks mark transitions included in Table S6.

Transition	ν	I^{TD}	I^{TI}	Δ	η_i^{def}	η_f^{def}	$\max_k(\mathrm{J}_{ik}^2)$
			$0 \to 1_i$				
$\|0\rangle \to \|1_3\rangle$	269.99	1.448	1.442	0.46	99.78	98.92	0.987
$\|0\rangle \to \|1_6\rangle$	456.00	3.863	3.862	0.02	99.78	99.01	0.974
$\|0\rangle \to \|1_8\rangle$	567.81	38.537	38.535	0.01	99.78	99.00	0.996
$\|0\rangle \to \|1_9\rangle$	633.87	7.561	7.545	0.20	99.78	99.02	0.800
$\|0\rangle \to \|1_{12}\rangle$	815.58	32.823	32.814	0.03	99.78	99.37	0.994
$\|0\rangle \to \|1_{17}\rangle$	1022.79	32.614	32.586	0.08	99.78	99.33	0.991
$\|0\rangle \to \|1_{18}\rangle$	1062.72	25.625	25.606	0.07	99.78	98.85	0.989
$\|0\rangle \to \|1_{20}\rangle$	1118.27	2.371	2.368	0.10	99.78	98.50	0.782
$\|0\rangle \to \|1_{21}\rangle$	1177.07	2.078	2.078	0.02	99.78	98.55	0.793
$\|0\rangle \to \|1_{24}\rangle$	1215.54	1.564	1.563	0.09	99.78	98.53	0.776
$\|0\rangle \to \|1_{25}\rangle$	1300.81	17.288	17.270	0.11	99.78	98.91	0.952
$\|0\rangle \to \|1_{26}\rangle$	1339.20	31.323	31.304	0.06	99.78	98.85	0.978
$\|0\rangle \to \|1_{27}\rangle$	1364.63	8.360	8.358	0.03	99.78	98.78	0.923
$\|0\rangle \to \|1_{32}\rangle$	1556.51	5.370	5.373	0.05	99.78	98.64	0.788
$\|0\rangle \to \|1_{33}\rangle$	1675.97	6.467	6.460	0.11	99.78	98.50	0.444
$\|0\rangle \to \|1_{34}\rangle$	1695.04	3.107	3.097	0.32	99.78	98.45	0.705
			$0 \to 2_i$				
$\|0\rangle \to \|2_5\rangle$	862.53	3.604	3.590	0.40	99.78	97.55	0.770
$\|0\rangle \to \|2_8\rangle$	1135.63	4.277	4.273	0.10	99.78	98.17	0.996
$\|0\rangle \to \|2_{10}\rangle$	1435.68	2.790	2.792	0.06	99.78	96.92	0.500
$\|0\rangle \to \|2_{12}\rangle$	1631.16	3.370	3.361	0.24	99.78	98.94	0.994
$\|0\rangle \to \|2_{13}\rangle$	1705.89	4.689	4.690	0.02	99.78	97.69	0.848
$\|0\rangle \to \|2_{15}\rangle$	1990.39	3.168	3.171	0.08	99.78	97.27	0.758
$\|0\rangle \to \|2_{16}\rangle$	2011.11	1.505	1.510	0.35	99.78	96.27	0.480
$\|0\rangle \to \|2_{17}\rangle$	2045.58	2.957	2.947	0.35	99.78	98.78	0.991
$\|0\rangle \to \|2_{18}\rangle$	2125.44	2.263	2.256	0.34	99.78	98.24	0.989
$\|0\rangle \to \|2_{26}\rangle$	2678.39	1.208	1.207	0.04	99.78	96.86	0.978
			$0 \to 1_i 1_j$				

Continued on next page

Table S5: Full anisole default-intense OPA/RR validation data (continued).

	Transition	ν	I^{TD}	I^{TI}	Δ	η_i^{def}	η_f^{def}	$\max_k(\mathrm{J}_{ik}^2)$
	$\lvert 0\rangle \to \lvert 1_{12}\, 1_8\rangle$	1383.39	7.007	7.018	0.16	99.78	97.92	0.994
	$\lvert 0\rangle \to \lvert 1_{17}\, 1_8\rangle$	1590.60	5.932	5.922	0.17	99.78	97.90	0.991
	$\lvert 0\rangle \to \lvert 1_{18}\, 1_8\rangle$	1630.53	5.008	5.001	0.12	99.78	96.85	0.989
	$\lvert 0\rangle \to \lvert 1_{17}\, 1_{12}\rangle$	1838.37	5.946	5.955	0.15	99.78	98.52	0.991
	$\lvert 0\rangle \to \lvert 1_{25}\, 1_8\rangle$	1868.62	2.404	2.401	0.14	99.78	96.26	0.952
	$\lvert 0\rangle \to \lvert 1_{18}\, 1_{12}\rangle$	1878.30	4.483	4.496	0.28	99.78	97.62	0.989
	$\lvert 0\rangle \to \lvert 1_{26}\, 1_8\rangle$	1907.01	4.273	4.270	0.08	99.78	96.73	0.978
	$\lvert 0\rangle \to \lvert 1_{27}\, 1_8\rangle$	1932.45	1.152	1.152	0.04	99.78	96.06	0.923
	$\lvert 0\rangle \to \lvert 1_{18}\, 1_{17}\rangle$	2085.51	4.390	4.373	0.37	99.78	98.13	0.989
	$\lvert 0\rangle \to \lvert 1_{25}\, 1_{12}\rangle$	2116.39	2.537	2.537	0.00	99.78	97.29	0.952
	$\lvert 0\rangle \to \lvert 1_{26}\, 1_{12}\rangle$	2154.78	4.942	4.942	0.00	99.78	97.36	0.978
	$\lvert 0\rangle \to \lvert 1_{27}\, 1_{12}\rangle$	2180.21	1.270	1.270	0.01	99.78	96.94	0.923
	$\lvert 0\rangle \to \lvert 1_{25}\, 1_{17}\rangle$	2323.60	1.956	1.952	0.21	99.78	97.52	0.952
	$\lvert 0\rangle \to \lvert 1_{26}\, 1_{17}\rangle$	2361.98	4.288	4.282	0.14	99.78	97.52	0.978
	$\lvert 0\rangle \to \lvert 1_{25}\, 1_{18}\rangle$	2363.53	1.467	1.463	0.27	99.78	96.24	0.952
	$\lvert 0\rangle \to \lvert 1_{27}\, 1_{17}\rangle$	2387.42	1.169	1.168	0.11	99.78	96.85	0.923
	$\lvert 0\rangle \to \lvert 1_{26}\, 1_{18}\rangle$	2401.91	3.524	3.518	0.15	99.78	96.24	0.978
	$\lvert 0\rangle \to \lvert 1_{27}\, 1_{18}\rangle$	2427.35	1.016	1.015	0.10	99.78	93.88	0.923
	$\lvert 0\rangle \to \lvert 1_{26}\, 1_{25}\rangle$	2640.00	2.371	2.370	0.06	99.78	97.00	0.952
	$1_i \to 2_i$							
	$\lvert 1_{12}\rangle \to \lvert 2_{12}\rangle$	815.58	39.507	39.490	0.04	99.37	98.94	0.994
*	$\lvert 1_{13}\rangle \to \lvert 2_{13}\rangle$	852.95	1.352	1.326	2.00	95.25	97.69	0.848
	$\lvert 1_{17}\rangle \to \lvert 2_{17}\rangle$	1022.79	44.251	44.214	0.08	99.33	98.78	0.991
	$\lvert 1_{18}\rangle \to \lvert 2_{18}\rangle$	1062.72	36.707	36.665	0.12	98.85	98.24	0.989
	$\lvert 1_{20}\rangle \to \lvert 2_{20}\rangle$	1118.27	4.707	4.691	0.34	98.50	97.01	0.782
	$\lvert 1_{21}\rangle \to \lvert 2_{21}\rangle$	1177.07	3.838	3.847	0.22	98.55	94.44	0.793
	$\lvert 1_{24}\rangle \to \lvert 2_{24}\rangle$	1215.54	3.078	3.067	0.36	98.53	97.37	0.776
	$\lvert 1_{25}\rangle \to \lvert 2_{25}\rangle$	1300.81	27.108	27.087	0.08	98.91	96.35	0.952
	$\lvert 1_{26}\rangle \to \lvert 2_{26}\rangle$	1339.20	44.902	44.877	0.06	98.85	96.86	0.978
	$\lvert 1_{27}\rangle \to \lvert 2_{27}\rangle$	1364.63	14.921	14.924	0.02	98.78	97.00	0.923
	$\lvert 1_{32}\rangle \to \lvert 2_{32}\rangle$	1556.51	10.455	10.424	0.30	98.64	96.28	0.788
	$\lvert 1_{33}\rangle \to \lvert 2_{33}\rangle$	1675.97	12.795	12.681	0.90	98.50	96.00	0.444
*	$\lvert 1_{34}\rangle \to \lvert 2_{34}\rangle$	1695.04	6.466	6.350	1.82	98.45	97.08	0.705
*	$\lvert 1_{39}\rangle \to \lvert 2_{39}\rangle$	3210.05	1.331	1.310	1.56	98.20	97.78	0.981
	$\lvert 1_3\rangle \to \lvert 2_3\rangle$	269.99	2.791	2.788	0.08	98.92	98.53	0.987
	$\lvert 1_6\rangle \to \lvert 2_6\rangle$	456.00	7.465	7.450	0.21	99.01	98.42	0.974

Continued on next page

Table S5: Full anisole default-intense OPA/RR validation data (continued).

	Transition	ν	I^{TD}	I^{TI}	Δ	η_i^{def}	η_f^{def}	$\max_k(\mathrm{J}_{ik}^2)$
	$\|1_8\rangle \rightarrow \|2_8\rangle$	567.81	55.318	55.313	0.01	99.00	98.17	0.996
	$\|1_9\rangle \rightarrow \|2_9\rangle$	633.87	15.816	15.722	0.60	99.02	98.02	0.800
				$1_i \rightarrow 3_i$				
	$\|1_{10}\rangle \rightarrow \|3_{10}\rangle$	1435.68	7.546	7.465	1.07	96.27	81.77	0.500
	$\|1_{11}\rangle \rightarrow \|3_{11}\rangle$	1574.03	1.612	1.610	0.15	94.72	85.28	0.824
	$\|1_{12}\rangle \rightarrow \|3_{12}\rangle$	1631.16	5.914	5.902	0.20	99.37	98.63	0.994
	$\|1_{13}\rangle \rightarrow \|3_{13}\rangle$	1705.89	12.221	12.104	0.97	95.25	86.02	0.848
	$\|1_{14}\rangle \rightarrow \|3_{14}\rangle$	1849.98	2.581	2.580	0.03	96.54	83.30	0.667
	$\|1_{15}\rangle \rightarrow \|3_{15}\rangle$	1990.39	9.198	9.142	0.61	94.80	82.63	0.758
	$\|1_{16}\rangle \rightarrow \|3_{16}\rangle$	2011.11	4.534	4.539	0.11	95.91	76.04	0.480
	$\|1_{17}\rangle \rightarrow \|3_{17}\rangle$	2045.58	6.061	6.039	0.36	99.33	97.96	0.991
	$\|1_{18}\rangle \rightarrow \|3_{18}\rangle$	2125.44	5.015	4.998	0.35	98.85	97.58	0.989
	$\|1_{26}\rangle \rightarrow \|3_{26}\rangle$	2678.39	2.399	2.399	0.01	98.85	93.95	0.978
*	$\|1_{33}\rangle \rightarrow \|3_{33}\rangle$	3351.95	1.534	1.551	1.06	98.50	90.41	0.444
	$\|1_4\rangle \rightarrow \|3_4\rangle$	567.78	1.877	1.841	1.95	96.89	87.42	0.756
	$\|1_5\rangle \rightarrow \|3_5\rangle$	862.53	7.236	6.765	6.97	93.75	85.15	0.770
	$\|1_8\rangle \rightarrow \|3_8\rangle$	1135.63	8.492	8.469	0.27	99.00	97.24	0.996
	$\|1_9\rangle \rightarrow \|3_9\rangle$	1267.74	2.177	2.176	0.08	99.02	96.92	0.800
				$1_i \rightarrow 1_i1_j$				
	$\|1_{11}\rangle \rightarrow \|1_{11}\,1_8\rangle$	567.81	33.827	33.982	0.46	94.72	84.57	0.824
	$\|1_{11}\rangle \rightarrow \|1_{12}\,1_{11}\rangle$	815.58	32.785	32.543	0.74	94.72	89.81	0.824
	$\|1_{11}\rangle \rightarrow \|1_{17}\,1_{11}\rangle$	1022.79	32.918	32.881	0.11	94.72	90.24	0.824
	$\|1_{14}\rangle \rightarrow \|1_{14}\,1_8\rangle$	567.81	31.541	31.494	0.15	96.54	89.51	0.667
	$\|1_{14}\rangle \rightarrow \|1_{14}\,1_{12}\rangle$	815.58	32.700	32.472	0.70	96.54	90.96	0.667
	$\|1_{14}\rangle \rightarrow \|1_{17}\,1_{14}\rangle$	1022.79	33.049	32.982	0.20	96.54	93.08	0.667
*	$\|1_{16}\rangle \rightarrow \|1_{16}\,1_{12}\rangle$	815.58	30.998	30.624	1.22	95.91	92.50	0.480
	$\|1_{16}\rangle \rightarrow \|1_{17}\,1_{16}\rangle$	1022.79	32.134	32.012	0.38	95.91	91.88	0.480
	$\|1_{19}\rangle \rightarrow \|1_{19}\,1_8\rangle$	567.81	37.216	37.232	0.04	98.77	95.57	0.650
	$\|1_{19}\rangle \rightarrow \|1_{19}\,1_{12}\rangle$	815.58	33.131	33.093	0.11	98.77	96.96	0.650
	$\|1_{19}\rangle \rightarrow \|1_{19}\,1_{17}\rangle$	1022.79	32.894	32.880	0.04	98.77	97.13	0.650
	$\|1_{19}\rangle \rightarrow \|1_{26}\,1_{19}\rangle$	1339.20	31.157	31.160	0.01	98.77	95.62	0.650
	$\|1_1\rangle \rightarrow \|1_8\,1_1\rangle$	567.81	37.904	38.065	0.42	97.82	93.90	0.596
	$\|1_1\rangle \rightarrow \|1_{12}\,1_1\rangle$	815.58	31.931	31.941	0.03	97.82	95.42	0.596
	$\|1_1\rangle \rightarrow \|1_{17}\,1_1\rangle$	1022.79	31.782	31.784	0.01	97.82	95.87	0.596
	$\|1_{20}\rangle \rightarrow \|1_{20}\,1_8\rangle$	567.81	36.272	36.313	0.11	98.50	95.56	0.782

Continued on next page

Table S5: Full anisole default-intense OPA/RR validation data (continued).

Transition	ν	I^{TD}	I^{TI}	Δ	η_i^{def}	η_f^{def}	$\max_k(\mathrm{J}_{ik}^2)$
$\lvert 1_{20}\rangle \rightarrow \lvert 1_{20}\,1_{12}\rangle$	815.58	32.423	32.382	0.13	98.50	96.94	0.782
$\lvert 1_{20}\rangle \rightarrow \lvert 1_{20}\,1_{17}\rangle$	1022.79	32.168	32.154	0.04	98.50	96.90	0.782
$\lvert 1_{21}\rangle \rightarrow \lvert 1_{21}\,1_{8}\rangle$	567.81	35.041	35.141	0.28	98.55	95.04	0.793
$\lvert 1_{22}\rangle \rightarrow \lvert 1_{22}\,1_{8}\rangle$	567.81	38.274	38.565	0.75	97.21	90.16	0.996
$\lvert 1_{22}\rangle \rightarrow \lvert 1_{22}\,1_{12}\rangle$	815.58	33.057	33.066	0.03	97.21	93.54	0.994
$\lvert 1_{22}\rangle \rightarrow \lvert 1_{22}\,1_{17}\rangle$	1022.79	32.817	32.852	0.11	97.21	93.73	0.991
$\lvert 1_{22}\rangle \rightarrow \lvert 1_{26}\,1_{22}\rangle$	1339.20	31.380	31.432	0.17	97.21	88.39	0.978
$\lvert 1_{23}\rangle \rightarrow \lvert 1_{23}\,1_{8}\rangle$	567.81	37.901	38.043	0.37	97.82	92.94	0.940
$\lvert 1_{23}\rangle \rightarrow \lvert 1_{23}\,1_{12}\rangle$	815.58	33.200	33.190	0.03	97.82	95.18	0.940
$\lvert 1_{23}\rangle \rightarrow \lvert 1_{23}\,1_{17}\rangle$	1022.79	32.942	32.957	0.04	97.82	95.41	0.940
$\lvert 1_{23}\rangle \rightarrow \lvert 1_{26}\,1_{23}\rangle$	1339.20	31.275	31.318	0.14	97.82	93.06	0.940
$\lvert 1_{24}\rangle \rightarrow \lvert 1_{24}\,1_{8}\rangle$	567.81	37.043	37.118	0.20	98.53	95.39	0.776
$\lvert 1_{24}\rangle \rightarrow \lvert 1_{24}\,1_{12}\rangle$	815.58	31.605	31.593	0.04	98.53	96.83	0.776
$\lvert 1_{24}\rangle \rightarrow \lvert 1_{24}\,1_{17}\rangle$	1022.79	31.448	31.443	0.01	98.53	96.87	0.776
$\lvert 1_{27}\rangle \rightarrow \lvert 1_{27}\,1_{8}\rangle$	567.81	32.056	32.102	0.14	98.78	96.06	0.923
$\lvert 1_{28}\rangle \rightarrow \lvert 1_{28}\,1_{8}\rangle$	567.81	38.213	38.379	0.43	97.82	93.98	0.729
$\lvert 1_{28}\rangle \rightarrow \lvert 1_{28}\,1_{12}\rangle$	815.58	32.913	32.923	0.03	97.82	94.02	0.729
$\lvert 1_{28}\rangle \rightarrow \lvert 1_{28}\,1_{17}\rangle$	1022.79	32.703	32.725	0.07	97.82	94.76	0.729
$\lvert 1_{28}\rangle \rightarrow \lvert 1_{28}\,1_{26}\rangle$	1339.20	31.332	31.380	0.15	97.82	94.32	0.729
$\lvert 1_{29}\rangle \rightarrow \lvert 1_{29}\,1_{8}\rangle$	567.81	38.451	38.665	0.55	97.53	89.63	0.996
$\lvert 1_{29}\rangle \rightarrow \lvert 1_{29}\,1_{12}\rangle$	815.58	32.959	32.988	0.09	97.53	94.01	0.994
$\lvert 1_{29}\rangle \rightarrow \lvert 1_{29}\,1_{17}\rangle$	1022.79	32.723	32.746	0.07	97.53	94.26	0.991
$\lvert 1_{29}\rangle \rightarrow \lvert 1_{29}\,1_{26}\rangle$	1339.20	31.358	31.445	0.28	97.53	90.76	0.978
$\lvert 1_{2}\rangle \rightarrow \lvert 1_{8}\,1_{2}\rangle$	567.81	37.184	37.714	1.41	94.49	85.94	0.598
$\lvert 1_{2}\rangle \rightarrow \lvert 1_{12}\,1_{2}\rangle$	815.58	33.047	33.074	0.08	94.49	83.73	0.598
$\lvert 1_{2}\rangle \rightarrow \lvert 1_{17}\,1_{2}\rangle$	1022.79	32.863	32.959	0.29	94.49	90.06	0.598
$\lvert 1_{2}\rangle \rightarrow \lvert 1_{26}\,1_{2}\rangle$	1339.20	31.195	31.484	0.92	94.49	83.76	0.598
$\lvert 1_{30}\rangle \rightarrow \lvert 1_{30}\,1_{8}\rangle$	567.81	37.400	37.458	0.16	98.46	95.40	0.265
$\lvert 1_{30}\rangle \rightarrow \lvert 1_{30}\,1_{12}\rangle$	815.58	33.090	33.060	0.09	98.46	96.83	0.265
$\lvert 1_{30}\rangle \rightarrow \lvert 1_{30}\,1_{17}\rangle$	1022.79	32.931	32.921	0.03	98.46	96.85	0.265
$\lvert 1_{30}\rangle \rightarrow \lvert 1_{30}\,1_{26}\rangle$	1339.20	31.288	31.304	0.05	98.46	95.15	0.265
$\lvert 1_{31}\rangle \rightarrow \lvert 1_{31}\,1_{8}\rangle$	567.81	37.507	37.584	0.20	98.36	94.92	0.309
$\lvert 1_{31}\rangle \rightarrow \lvert 1_{31}\,1_{12}\rangle$	815.58	33.078	33.053	0.08	98.36	96.64	0.309
$\lvert 1_{31}\rangle \rightarrow \lvert 1_{31}\,1_{17}\rangle$	1022.79	32.896	32.890	0.02	98.36	96.62	0.309
$\lvert 1_{31}\rangle \rightarrow \lvert 1_{31}\,1_{26}\rangle$	1339.20	31.303	31.329	0.08	98.36	95.33	0.309
$\lvert 1_{32}\rangle \rightarrow \lvert 1_{32}\,1_{8}\rangle$	567.81	35.157	35.174	0.05	98.64	95.48	0.788
$\lvert 1_{32}\rangle \rightarrow \lvert 1_{32}\,1_{12}\rangle$	815.58	32.274	32.216	0.18	98.64	96.97	0.788

Continued on next page

Table S5: Full anisole default-intense OPA/RR validation data (continued).

	Transition	ν	I^{TD}	I^{TI}	Δ	η_i^{def}	η_f^{def}	$\max_k(\mathrm{J}_{ik}^2)$
	$\lvert 1_{32}\rangle \rightarrow \lvert 1_{32}\, 1_{17}\rangle$	1022.79	31.867	31.848	0.06	98.64	97.10	0.788
	$\lvert 1_{33}\rangle \rightarrow \lvert 1_{33}\, 1_{12}\rangle$	815.58	32.401	32.253	0.46	98.50	96.72	0.444
	$\lvert 1_{33}\rangle \rightarrow \lvert 1_{33}\, 1_{17}\rangle$	1022.79	33.202	33.146	0.17	98.50	96.73	0.444
	$\lvert 1_{34}\rangle \rightarrow \lvert 1_{34}\, 1_{8}\rangle$	567.81	34.238	34.234	0.01	98.45	94.89	0.705
	$\lvert 1_{34}\rangle \rightarrow \lvert 1_{34}\, 1_{12}\rangle$	815.58	32.636	32.554	0.25	98.45	96.74	0.705
	$\lvert 1_{34}\rangle \rightarrow \lvert 1_{34}\, 1_{17}\rangle$	1022.79	32.677	32.649	0.09	98.45	96.70	0.705
	$\lvert 1_{35}\rangle \rightarrow \lvert 1_{35}\, 1_{8}\rangle$	567.81	38.434	38.554	0.31	98.34	95.25	0.903
	$\lvert 1_{35}\rangle \rightarrow \lvert 1_{35}\, 1_{12}\rangle$	815.58	31.849	31.871	0.07	98.34	96.61	0.903
	$\lvert 1_{35}\rangle \rightarrow \lvert 1_{35}\, 1_{17}\rangle$	1022.79	31.668	31.669	0.00	98.34	96.50	0.903
*	$\lvert 1_{36}\rangle \rightarrow \lvert 1_{36}\, 1_{8}\rangle$	567.81	38.521	39.112	1.51	95.70	90.74	0.996
	$\lvert 1_{36}\rangle \rightarrow \lvert 1_{36}\, 1_{12}\rangle$	815.58	31.535	31.784	0.78	95.70	90.31	0.994
	$\lvert 1_{36}\rangle \rightarrow \lvert 1_{36}\, 1_{17}\rangle$	1022.79	31.391	31.450	0.19	95.70	90.74	0.991
	$\lvert 1_{37}\rangle \rightarrow \lvert 1_{37}\, 1_{8}\rangle$	567.81	38.422	38.621	0.51	97.96	94.11	0.991
	$\lvert 1_{37}\rangle \rightarrow \lvert 1_{37}\, 1_{12}\rangle$	815.58	32.175	32.212	0.11	97.96	95.74	0.991
	$\lvert 1_{37}\rangle \rightarrow \lvert 1_{37}\, 1_{17}\rangle$	1022.79	31.976	31.999	0.07	97.96	95.36	0.991
	$\lvert 1_{38}\rangle \rightarrow \lvert 1_{38}\, 1_{8}\rangle$	567.81	38.318	38.447	0.34	98.26	95.05	0.470
	$\lvert 1_{38}\rangle \rightarrow \lvert 1_{38}\, 1_{12}\rangle$	815.58	31.848	31.870	0.07	98.26	96.43	0.470
	$\lvert 1_{38}\rangle \rightarrow \lvert 1_{38}\, 1_{17}\rangle$	1022.79	31.669	31.673	0.01	98.26	96.40	0.470
	$\lvert 1_{39}\rangle \rightarrow \lvert 1_{39}\, 1_{8}\rangle$	567.81	38.510	38.679	0.44	98.20	93.48	0.981
	$\lvert 1_{39}\rangle \rightarrow \lvert 1_{39}\, 1_{12}\rangle$	815.58	31.897	31.934	0.11	98.20	95.01	0.981
	$\lvert 1_{39}\rangle \rightarrow \lvert 1_{39}\, 1_{17}\rangle$	1022.79	31.720	31.735	0.05	98.20	95.11	0.981
	$\lvert 1_{3}\rangle \rightarrow \lvert 1_{8}\, 1_{3}\rangle$	567.81	37.004	37.038	0.09	98.92	96.72	0.987
	$\lvert 1_{3}\rangle \rightarrow \lvert 1_{12}\, 1_{3}\rangle$	815.58	31.599	31.585	0.04	98.92	97.46	0.987
	$\lvert 1_{3}\rangle \rightarrow \lvert 1_{17}\, 1_{3}\rangle$	1022.79	31.630	31.620	0.03	98.92	97.51	0.987
	$\lvert 1_{40}\rangle \rightarrow \lvert 1_{40}\, 1_{8}\rangle$	567.81	38.370	38.501	0.34	98.34	94.51	0.451
	$\lvert 1_{40}\rangle \rightarrow \lvert 1_{40}\, 1_{12}\rangle$	815.58	32.238	32.254	0.05	98.34	96.46	0.451
	$\lvert 1_{40}\rangle \rightarrow \lvert 1_{40}\, 1_{17}\rangle$	1022.79	32.055	32.062	0.02	98.34	96.11	0.451
	$\lvert 1_{41}\rangle \rightarrow \lvert 1_{41}\, 1_{8}\rangle$	567.81	38.147	38.269	0.32	98.31	95.16	0.565
	$\lvert 1_{41}\rangle \rightarrow \lvert 1_{41}\, 1_{12}\rangle$	815.58	31.960	31.980	0.06	98.31	96.60	0.565
	$\lvert 1_{41}\rangle \rightarrow \lvert 1_{41}\, 1_{17}\rangle$	1022.79	31.788	31.796	0.03	98.31	96.61	0.565
	$\lvert 1_{42}\rangle \rightarrow \lvert 1_{42}\, 1_{8}\rangle$	567.81	38.147	38.273	0.33	98.35	95.05	0.721
	$\lvert 1_{42}\rangle \rightarrow \lvert 1_{42}\, 1_{12}\rangle$	815.58	31.646	31.669	0.07	98.35	96.55	0.721
	$\lvert 1_{42}\rangle \rightarrow \lvert 1_{42}\, 1_{17}\rangle$	1022.79	31.513	31.518	0.02	98.35	96.47	0.721
	$\lvert 1_{4}\rangle \rightarrow \lvert 1_{8}\, 1_{4}\rangle$	567.81	35.546	35.698	0.43	96.89	90.44	0.756
	$\lvert 1_{4}\rangle \rightarrow \lvert 1_{12}\, 1_{4}\rangle$	815.58	32.827	32.741	0.26	96.89	93.80	0.756
	$\lvert 1_{4}\rangle \rightarrow \lvert 1_{17}\, 1_{4}\rangle$	1022.79	32.761	32.751	0.03	96.89	94.14	0.756
	$\lvert 1_{5}\rangle \rightarrow \lvert 1_{17}\, 1_{5}\rangle$	1022.79	31.166	31.044	0.39	93.75	89.43	0.770

Continued on next page

Table S5: Full anisole default-intense OPA/RR validation data (continued).

Transition	ν	I^{TD}	I^{TI}	Δ	η_i^{def}	η_f^{def}	$\max_k(\mathrm{J}_{ik}^2)$
$\lvert 1_6\rangle \to \lvert 1_8\, 1_6\rangle$	567.81	36.405	36.415	0.03	99.01	97.07	0.974
$\lvert 1_6\rangle \to \lvert 1_{12}\, 1_6\rangle$	815.58	31.387	31.368	0.06	99.01	97.54	0.974
$\lvert 1_6\rangle \to \lvert 1_{17}\, 1_6\rangle$	1022.79	31.343	31.333	0.03	99.01	97.48	0.974
$\lvert 1_7\rangle \to \lvert 1_8\, 1_7\rangle$	567.81	34.684	34.901	0.62	96.86	90.88	0.514
$\lvert 1_7\rangle \to \lvert 1_{12}\, 1_7\rangle$	815.58	31.424	31.377	0.15	96.86	93.19	0.514
$\lvert 1_7\rangle \to \lvert 1_{17}\, 1_7\rangle$	1022.79	31.610	31.609	0.00	96.86	93.41	0.514
$\lvert 1_9\rangle \to \lvert 1_9\, 1_8\rangle$	567.81	35.386	35.355	0.09	99.02	98.04	0.800
$\lvert 1_9\rangle \to \lvert 1_{12}\, 1_9\rangle$	815.58	33.551	33.489	0.19	99.02	97.70	0.800
$\lvert 1_9\rangle \to \lvert 1_{17}\, 1_9\rangle$	1022.79	33.516	33.490	0.08	99.02	97.57	0.800
$\lvert 1_9\rangle \to \lvert 1_{26}\, 1_9\rangle$	1339.20	31.243	31.241	0.01	99.02	96.44	0.800
$1_i \to 2_i 1_j$							
$\lvert 1_{10}\rangle \to \lvert 2_{10}\, 1_7\rangle$	1248.18	1.143	1.139	0.36	96.27	86.18	0.500
$\lvert 1_{12}\rangle \to \lvert 2_{12}\, 1_8\rangle$	1383.39	6.147	6.152	0.07	99.37	96.66	0.994
$\lvert 1_{12}\rangle \to \lvert 1_{17}\, 2_{12}\rangle$	1838.37	5.863	5.873	0.18	99.37	97.87	0.991
$\lvert 1_{12}\rangle \to \lvert 1_{18}\, 2_{12}\rangle$	1878.30	4.300	4.309	0.20	99.37	96.50	0.989
$\lvert 1_{12}\rangle \to \lvert 1_{25}\, 2_{12}\rangle$	2116.39	3.049	3.048	0.06	99.37	95.65	0.952
$\lvert 1_{12}\rangle \to \lvert 1_{26}\, 2_{12}\rangle$	2154.78	5.890	5.886	0.06	99.37	96.14	0.978
$\lvert 1_{12}\rangle \to \lvert 1_{27}\, 2_{12}\rangle$	2180.21	1.489	1.488	0.09	99.37	95.70	0.923
$\lvert 1_{12}\rangle \to \lvert 1_{32}\, 2_{12}\rangle$	2372.09	1.176	1.175	0.07	99.37	94.16	0.788
$\lvert 1_{17}\rangle \to \lvert 2_{17}\, 1_8\rangle$	1590.60	6.570	6.560	0.14	99.33	96.41	0.991
$\lvert 1_{17}\rangle \to \lvert 2_{17}\, 1_{12}\rangle$	1838.37	6.144	6.149	0.09	99.33	97.32	0.991
$\lvert 1_{17}\rangle \to \lvert 1_{18}\, 2_{17}\rangle$	2085.51	4.746	4.737	0.18	99.33	97.03	0.989
$\lvert 1_{17}\rangle \to \lvert 1_{25}\, 2_{17}\rangle$	2323.60	2.377	2.374	0.16	99.33	95.56	0.952
$\lvert 1_{17}\rangle \to \lvert 1_{26}\, 2_{17}\rangle$	2361.98	5.503	5.496	0.13	99.33	95.58	0.978
$\lvert 1_{17}\rangle \to \lvert 1_{27}\, 2_{17}\rangle$	2387.42	1.515	1.513	0.11	99.33	95.22	0.923
$\lvert 1_{18}\rangle \to \lvert 2_{18}\, 1_8\rangle$	1630.53	6.167	6.174	0.11	98.85	95.05	0.989
$\lvert 1_{18}\rangle \to \lvert 2_{18}\, 1_{12}\rangle$	1878.30	5.233	5.258	0.47	98.85	96.17	0.989
$\lvert 1_{18}\rangle \to \lvert 2_{18}\, 1_{17}\rangle$	2085.51	5.334	5.307	0.52	98.85	96.99	0.989
$\lvert 1_{18}\rangle \to \lvert 1_{25}\, 2_{18}\rangle$	2363.53	1.892	1.893	0.05	98.85	93.87	0.952
$\lvert 1_{18}\rangle \to \lvert 1_{26}\, 2_{18}\rangle$	2401.91	4.889	4.886	0.07	98.85	93.84	0.978
$\lvert 1_{18}\rangle \to \lvert 1_{27}\, 2_{18}\rangle$	2427.35	1.443	1.443	0.01	98.85	90.92	0.923
$\lvert 1_{21}\rangle \to \lvert 1_{25}\, 2_{21}\rangle$	2477.88	1.004	1.006	0.17	98.55	91.58	0.793
$\lvert 1_{25}\rangle \to \lvert 2_{25}\, 1_8\rangle$	1868.62	3.161	3.164	0.11	98.91	90.79	0.952
$\lvert 1_{25}\rangle \to \lvert 2_{25}\, 1_{12}\rangle$	2116.39	3.209	3.208	0.02	98.91	93.99	0.952
$\lvert 1_{25}\rangle \to \lvert 2_{25}\, 1_{17}\rangle$	2323.60	2.452	2.452	0.01	98.91	93.43	0.952
$\lvert 1_{25}\rangle \to \lvert 2_{25}\, 1_{18}\rangle$	2363.53	1.776	1.777	0.02	98.91	90.02	0.952

Continued on next page

Table S5: Full anisole default-intense OPA/RR validation data (continued).

Transition	ν	I^{TD}	I^{TI}	Δ	η_i^{def}	η_f^{def}	$\max_k(\mathrm{J}_{ik}^2)$
$\vert 1_{25}\rangle \rightarrow \vert 1_{26}\, 2_{25}\rangle$	2640.00	3.394	3.393	0.05	98.91	93.44	0.952
$\vert 1_{25}\rangle \rightarrow \vert 1_{27}\, 2_{25}\rangle$	2665.44	1.339	1.337	0.12	98.91	91.87	0.923
$\vert 1_{26}\rangle \rightarrow \vert 2_{26}\, 1_{8}\rangle$	1907.01	5.021	5.021	0.00	98.85	92.93	0.978
$\vert 1_{26}\rangle \rightarrow \vert 2_{26}\, 1_{12}\rangle$	2154.78	5.666	5.665	0.03	98.85	93.94	0.978
$\vert 1_{26}\rangle \rightarrow \vert 2_{26}\, 1_{17}\rangle$	2361.98	4.913	4.910	0.06	98.85	93.62	0.978
$\vert 1_{26}\rangle \rightarrow \vert 2_{26}\, 1_{18}\rangle$	2401.91	3.984	3.982	0.04	98.85	91.69	0.978
$\vert 1_{26}\rangle \rightarrow \vert 2_{26}\, 1_{25}\rangle$	2640.00	3.264	3.262	0.07	98.85	92.88	0.952
$\vert 1_{27}\rangle \rightarrow \vert 2_{27}\, 1_{8}\rangle$	1932.45	1.921	1.921	0.01	98.78	92.92	0.923
$\vert 1_{27}\rangle \rightarrow \vert 2_{27}\, 1_{12}\rangle$	2180.21	2.128	2.128	0.01	98.78	94.07	0.923
$\vert 1_{27}\rangle \rightarrow \vert 2_{27}\, 1_{17}\rangle$	2387.42	1.970	1.969	0.08	98.78	93.90	0.923
$\vert 1_{27}\rangle \rightarrow \vert 2_{27}\, 1_{18}\rangle$	2427.35	1.713	1.712	0.04	98.78	92.06	0.923
$\vert 1_{27}\rangle \rightarrow \vert 2_{27}\, 1_{25}\rangle$	2665.44	1.618	1.619	0.01	98.78	91.88	0.923
$\vert 1_{32}\rangle \rightarrow \vert 2_{32}\, 1_{8}\rangle$	2124.32	1.126	1.123	0.21	98.64	90.63	0.788
$\vert 1_{32}\rangle \rightarrow \vert 2_{32}\, 1_{12}\rangle$	2372.09	1.762	1.761	0.05	98.64	92.61	0.788
$\vert 1_{32}\rangle \rightarrow \vert 2_{32}\, 1_{17}\rangle$	2579.30	1.331	1.328	0.25	98.64	92.66	0.788
$\vert 1_{33}\rangle \rightarrow \vert 2_{33}\, 1_{8}\rangle$	2243.79	1.044	1.044	0.02	98.50	89.30	0.444
$\vert 1_{33}\rangle \rightarrow \vert 2_{33}\, 1_{17}\rangle$	2698.76	1.038	1.037	0.06	98.50	91.16	0.444
$\vert 1_{6}\rangle \rightarrow \vert 1_{8}\, 2_{6}\rangle$	1023.82	1.709	1.709	0.04	99.01	96.15	0.974
$\vert 1_{6}\rangle \rightarrow \vert 1_{12}\, 2_{6}\rangle$	1271.59	1.334	1.331	0.22	99.01	96.61	0.974
$\vert 1_{7}\rangle \rightarrow \vert 1_{10}\, 2_{7}\rangle$	1248.18	1.297	1.292	0.39	96.86	86.63	0.500
$\vert 1_{8}\rangle \rightarrow \vert 1_{12}\, 2_{8}\rangle$	1383.39	8.344	8.355	0.13	99.00	96.38	0.994
$\vert 1_{8}\rangle \rightarrow \vert 1_{17}\, 2_{8}\rangle$	1590.60	7.662	7.654	0.10	99.00	96.29	0.991
$\vert 1_{8}\rangle \rightarrow \vert 1_{18}\, 2_{8}\rangle$	1630.53	6.698	6.697	0.01	99.00	94.76	0.989
$\vert 1_{8}\rangle \rightarrow \vert 1_{25}\, 2_{8}\rangle$	1868.62	3.367	3.364	0.11	99.00	91.96	0.952
$\vert 1_{8}\rangle \rightarrow \vert 1_{26}\, 2_{8}\rangle$	1907.01	5.825	5.824	0.03	99.00	94.37	0.978
$\vert 1_{8}\rangle \rightarrow \vert 1_{27}\, 2_{8}\rangle$	1932.45	1.572	1.576	0.25	99.00	90.95	0.923
$\vert 1_{9}\rangle \rightarrow \vert 2_{9}\, 1_{8}\rangle$	1201.68	1.040	1.035	0.42	99.02	97.10	0.800
$\vert 1_{9}\rangle \rightarrow \vert 1_{12}\, 2_{9}\rangle$	1449.45	1.251	1.256	0.36	99.02	95.85	0.800
$\vert 1_{9}\rangle \rightarrow \vert 1_{17}\, 2_{9}\rangle$	1656.66	1.229	1.230	0.05	99.02	95.75	0.800
$\vert 1_{9}\rangle \rightarrow \vert 1_{18}\, 2_{9}\rangle$	1696.59	1.070	1.073	0.30	99.02	92.01	0.800
$\vert 1_{9}\rangle \rightarrow \vert 1_{26}\, 2_{9}\rangle$	1973.07	1.144	1.147	0.22	99.02	93.37	0.800
			$1_i \rightarrow 1_i 2_j$				
$\vert 1_{10}\rangle \rightarrow \vert 2_{12}\, 1_{10}\rangle$	1631.16	3.853	3.823	0.79	96.27	82.84	0.500
$\vert 1_{10}\rangle \rightarrow \vert 2_{13}\, 1_{10}\rangle$	1705.89	4.306	4.258	1.12	96.27	73.72	0.500
$\vert 1_{11}\rangle \rightarrow \vert 1_{11}\, 2_{8}\rangle$	1135.63	4.106	4.060	1.11	94.72	76.59	0.824
$\vert 1_{11}\rangle \rightarrow \vert 2_{12}\, 1_{11}\rangle$	1631.16	3.820	3.675	3.94	94.72	84.55	0.824

Continued on next page

Table S5: Full anisole default-intense OPA/RR validation data (continued).

	Transition	ν	I^{TD}	I^{TI}	Δ	η_i^{def}	η_f^{def}	$\max_k(\mathrm{J}_{ik}^2)$
	$\vert 1_{11}\rangle \to \vert 2_{13}\,1_{11}\rangle$	1705.89	4.680	4.685	0.09	94.72	71.53	0.824
	$\vert 1_{13}\rangle \to \vert 1_{13}\,2_{12}\rangle$	1631.16	4.007	3.825	4.75	95.25	82.07	0.848
	$\vert 1_{14}\rangle \to \vert 1_{14}\,2_{8}\rangle$	1135.63	3.997	4.027	0.76	96.54	77.03	0.667
	$\vert 1_{14}\rangle \to \vert 1_{14}\,2_{12}\rangle$	1631.16	4.048	3.944	2.64	96.54	84.90	0.667
	$\vert 1_{14}\rangle \to \vert 1_{14}\,2_{13}\rangle$	1705.89	4.688	4.660	0.58	96.54	79.29	0.667
	$\vert 1_{15}\rangle \to \vert 1_{15}\,2_{12}\rangle$	1631.16	4.176	3.969	5.22	94.80	82.39	0.758
	$\vert 1_{15}\rangle \to \vert 1_{15}\,2_{13}\rangle$	1705.89	4.134	4.039	2.36	94.80	78.22	0.758
	$\vert 1_{15}\rangle \to \vert 2_{17}\,1_{15}\rangle$	2045.58	3.543	3.261	8.66	94.80	83.70	0.758
	$\vert 1_{16}\rangle \to \vert 1_{16}\,2_{12}\rangle$	1631.16	4.065	3.901	4.20	95.91	84.52	0.480
	$\vert 1_{16}\rangle \to \vert 1_{16}\,2_{13}\rangle$	1705.89	4.500	4.445	1.24	95.91	72.74	0.480
	$\vert 1_{19}\rangle \to \vert 1_{19}\,2_{5}\rangle$	862.53	3.513	3.430	2.39	98.77	87.17	0.650
	$\vert 1_{19}\rangle \to \vert 1_{19}\,2_{8}\rangle$	1135.63	4.315	4.305	0.25	98.77	89.08	0.650
	$\vert 1_{19}\rangle \to \vert 1_{19}\,2_{12}\rangle$	1631.16	3.562	3.542	0.57	98.77	93.53	0.650
	$\vert 1_{19}\rangle \to \vert 1_{19}\,2_{13}\rangle$	1705.89	4.724	4.734	0.21	98.77	87.86	0.650
	$\vert 1_{1}\rangle \to \vert 2_{5}\,1_{1}\rangle$	862.53	3.512	3.475	1.07	97.82	82.29	0.596
	$\vert 1_{1}\rangle \to \vert 2_{8}\,1_{1}\rangle$	1135.63	4.123	4.123	0.01	97.82	85.53	0.596
	$\vert 1_{1}\rangle \to \vert 2_{13}\,1_{1}\rangle$	1705.89	4.627	4.651	0.52	97.82	79.67	0.596
	$\vert 1_{20}\rangle \to \vert 1_{20}\,2_{8}\rangle$	1135.63	4.215	4.198	0.40	98.50	88.94	0.782
	$\vert 1_{20}\rangle \to \vert 1_{20}\,2_{13}\rangle$	1705.89	4.625	4.635	0.23	98.50	87.89	0.782
	$\vert 1_{21}\rangle \to \vert 1_{21}\,2_{8}\rangle$	1135.63	3.692	3.682	0.26	98.55	88.98	0.793
	$\vert 1_{21}\rangle \to \vert 1_{21}\,2_{13}\rangle$	1705.89	4.206	4.237	0.72	98.55	87.69	0.793
	$\vert 1_{22}\rangle \to \vert 1_{22}\,2_{5}\rangle$	862.53	3.596	3.501	2.70	97.21	75.78	0.770
	$\vert 1_{22}\rangle \to \vert 1_{22}\,2_{8}\rangle$	1135.63	4.311	4.338	0.62	97.21	81.48	0.996
	$\vert 1_{22}\rangle \to \vert 1_{22}\,2_{13}\rangle$	1705.89	4.713	4.753	0.85	97.21	77.49	0.848
	$\vert 1_{23}\rangle \to \vert 1_{23}\,2_{5}\rangle$	862.53	3.569	3.436	3.86	97.82	79.03	0.770
	$\vert 1_{23}\rangle \to \vert 1_{23}\,2_{8}\rangle$	1135.63	4.321	4.299	0.49	97.82	84.77	0.940
*	$\vert 1_{23}\rangle \to \vert 1_{23}\,2_{12}\rangle$	1631.16	3.513	3.463	1.43	97.82	91.00	0.940
	$\vert 1_{23}\rangle \to \vert 1_{23}\,2_{13}\rangle$	1705.89	4.731	4.789	1.23	97.82	82.64	0.848
	$\vert 1_{24}\rangle \to \vert 1_{24}\,2_{8}\rangle$	1135.63	4.148	4.135	0.32	98.53	89.02	0.776
	$\vert 1_{24}\rangle \to \vert 1_{24}\,2_{13}\rangle$	1705.89	4.523	4.545	0.47	98.53	87.89	0.776
	$\vert 1_{27}\rangle \to \vert 1_{27}\,2_{13}\rangle$	1705.89	3.850	3.867	0.44	98.78	91.05	0.848
	$\vert 1_{28}\rangle \to \vert 1_{28}\,2_{5}\rangle$	862.53	3.577	3.471	3.06	97.82	78.66	0.729
	$\vert 1_{28}\rangle \to \vert 1_{28}\,2_{8}\rangle$	1135.63	4.279	4.260	0.44	97.82	86.68	0.729
	$\vert 1_{28}\rangle \to \vert 1_{28}\,2_{13}\rangle$	1705.89	4.701	4.769	1.42	97.82	82.35	0.729
	$\vert 1_{29}\rangle \to \vert 1_{29}\,2_{5}\rangle$	862.53	3.603	3.509	2.67	97.53	82.25	0.770
	$\vert 1_{29}\rangle \to \vert 1_{29}\,2_{8}\rangle$	1135.63	4.295	4.286	0.23	97.53	80.67	0.996
	$\vert 1_{29}\rangle \to \vert 1_{29}\,2_{13}\rangle$	1705.89	4.705	4.755	1.07	97.53	81.88	0.848

Continued on next page

Table S5: Full anisole default-intense OPA/RR validation data (continued).

	Transition	ν	I^{TD}	I^{TI}	Δ	η_i^{def}	η_f^{def}	$\max_k(\text{J}_{ik}^2)$
	$\|1_2\rangle \rightarrow \|2_5\, 1_2\rangle$	862.53	3.487	3.359	3.81	94.49	75.73	0.598
	$\|1_2\rangle \rightarrow \|2_8\, 1_2\rangle$	1135.63	4.281	4.224	1.36	94.49	74.74	0.598
	$\|1_2\rangle \rightarrow \|2_{12}\, 1_2\rangle$	1631.16	3.551	3.401	4.41	94.49	74.33	0.598
	$\|1_2\rangle \rightarrow \|2_{13}\, 1_2\rangle$	1705.89	4.712	4.787	1.56	94.49	80.99	0.598
	$\|1_{30}\rangle \rightarrow \|1_{30}\, 2_5\rangle$	862.53	3.516	3.430	2.50	98.46	86.87	0.265
	$\|1_{30}\rangle \rightarrow \|1_{30}\, 2_8\rangle$	1135.63	4.269	4.251	0.42	98.46	88.30	0.265
	$\|1_{30}\rangle \rightarrow \|1_{30}\, 2_{12}\rangle$	1631.16	3.533	3.502	0.89	98.46	94.01	0.265
	$\|1_{30}\rangle \rightarrow \|1_{30}\, 2_{13}\rangle$	1705.89	4.726	4.756	0.62	98.46	87.51	0.265
	$\|1_{31}\rangle \rightarrow \|1_{31}\, 2_5\rangle$	862.53	3.524	3.426	2.86	98.36	83.00	0.309
	$\|1_{31}\rangle \rightarrow \|1_{31}\, 2_8\rangle$	1135.63	4.268	4.258	0.24	98.36	88.88	0.309
	$\|1_{31}\rangle \rightarrow \|1_{31}\, 2_{12}\rangle$	1631.16	3.509	3.475	0.97	98.36	93.49	0.309
	$\|1_{31}\rangle \rightarrow \|1_{31}\, 2_{13}\rangle$	1705.89	4.723	4.758	0.75	98.36	87.56	0.309
	$\|1_{32}\rangle \rightarrow \|1_{32}\, 2_8\rangle$	1135.63	4.046	4.028	0.44	98.64	88.90	0.788
	$\|1_{32}\rangle \rightarrow \|1_{32}\, 2_{13}\rangle$	1705.89	4.560	4.563	0.08	98.64	87.84	0.788
	$\|1_{33}\rangle \rightarrow \|1_{33}\, 2_8\rangle$	1135.63	3.874	3.837	0.96	98.50	88.10	0.444
*	$\|1_{33}\rangle \rightarrow \|1_{33}\, 2_{12}\rangle$	1631.16	4.135	4.081	1.31	98.50	93.43	0.444
	$\|1_{33}\rangle \rightarrow \|1_{33}\, 2_{13}\rangle$	1705.89	4.697	4.667	0.64	98.50	87.26	0.444
*	$\|1_{33}\rangle \rightarrow \|1_{33}\, 2_{17}\rangle$	2045.58	3.470	3.435	1.01	98.50	92.36	0.444
	$\|1_{34}\rangle \rightarrow \|1_{34}\, 2_8\rangle$	1135.63	4.124	4.098	0.63	98.45	88.57	0.705
*	$\|1_{34}\rangle \rightarrow \|1_{34}\, 2_{12}\rangle$	1631.16	3.761	3.720	1.10	98.45	93.89	0.705
	$\|1_{34}\rangle \rightarrow \|1_{34}\, 2_{13}\rangle$	1705.89	4.665	4.662	0.07	98.45	87.20	0.705
	$\|1_{35}\rangle \rightarrow \|1_{35}\, 2_5\rangle$	862.53	3.549	3.518	0.86	98.34	86.95	0.770
	$\|1_{35}\rangle \rightarrow \|1_{35}\, 2_8\rangle$	1135.63	4.116	4.108	0.20	98.34	88.45	0.903
	$\|1_{35}\rangle \rightarrow \|1_{35}\, 2_{13}\rangle$	1705.89	4.557	4.590	0.72	98.34	87.65	0.848
	$\|1_{36}\rangle \rightarrow \|1_{36}\, 2_5\rangle$	862.53	3.534	3.512	0.61	95.70	73.88	0.770
	$\|1_{36}\rangle \rightarrow \|1_{36}\, 2_8\rangle$	1135.63	4.073	4.069	0.10	95.70	85.81	0.996
	$\|1_{36}\rangle \rightarrow \|1_{36}\, 2_{13}\rangle$	1705.89	4.519	4.630	2.39	95.70	79.76	0.848
	$\|1_{37}\rangle \rightarrow \|1_{37}\, 2_5\rangle$	862.53	3.563	3.512	1.46	97.96	78.42	0.770
	$\|1_{37}\rangle \rightarrow \|1_{37}\, 2_8\rangle$	1135.63	4.181	4.170	0.27	97.96	88.47	0.991
	$\|1_{37}\rangle \rightarrow \|1_{37}\, 2_{13}\rangle$	1705.89	4.601	4.678	1.65	97.96	82.02	0.848
	$\|1_{38}\rangle \rightarrow \|1_{38}\, 2_5\rangle$	862.53	3.540	3.505	0.99	98.26	86.09	0.470
	$\|1_{38}\rangle \rightarrow \|1_{38}\, 2_8\rangle$	1135.63	4.122	4.113	0.23	98.26	87.81	0.470
	$\|1_{38}\rangle \rightarrow \|1_{38}\, 2_{13}\rangle$	1705.89	4.559	4.593	0.74	98.26	87.33	0.470
	$\|1_{39}\rangle \rightarrow \|1_{39}\, 2_5\rangle$	862.53	3.555	3.520	0.98	98.20	78.68	0.770
	$\|1_{39}\rangle \rightarrow \|1_{39}\, 2_8\rangle$	1135.63	4.134	4.129	0.13	98.20	85.28	0.981
	$\|1_{39}\rangle \rightarrow \|1_{39}\, 2_{13}\rangle$	1705.89	4.565	4.628	1.36	98.20	81.98	0.848
	$\|1_3\rangle \rightarrow \|2_8\, 1_3\rangle$	1135.63	4.013	4.008	0.11	98.92	93.73	0.987

Continued on next page

Table S5: Full anisole default-intense OPA/RR validation data (continued).

Transition	ν	I^{TD}	I^{TI}	Δ	η_i^{def}	η_f^{def}	$\max_k(\mathrm{J}_{ik}^2)$
$\|1_3\rangle \to \|2_{13}\,1_3\rangle$	1705.89	4.541	4.556	0.32	98.92	91.34	0.848
$\|1_{40}\rangle \to \|1_{40}\,2_5\rangle$	862.53	3.562	3.517	1.28	98.34	82.29	0.451
$\|1_{40}\rangle \to \|1_{40}\,2_8\rangle$	1135.63	4.181	4.176	0.13	98.34	87.55	0.451
$\|1_{40}\rangle \to \|1_{40}\,2_{13}\rangle$	1705.89	4.610	4.666	1.20	98.34	86.85	0.451
$\|1_{41}\rangle \to \|1_{41}\,2_5\rangle$	862.53	3.536	3.491	1.29	98.31	86.52	0.565
$\|1_{41}\rangle \to \|1_{41}\,2_8\rangle$	1135.63	4.144	4.132	0.30	98.31	88.40	0.565
$\|1_{41}\rangle \to \|1_{41}\,2_{13}\rangle$	1705.89	4.573	4.630	1.24	98.31	87.34	0.565
$\|1_{42}\rangle \to \|1_{42}\,2_5\rangle$	862.53	3.525	3.491	0.96	98.35	86.19	0.721
$\|1_{42}\rangle \to \|1_{42}\,2_8\rangle$	1135.63	4.101	4.092	0.24	98.35	88.40	0.721
$\|1_{42}\rangle \to \|1_{42}\,2_{13}\rangle$	1705.89	4.531	4.565	0.75	98.35	87.07	0.721
$\|1_4\rangle \to \|2_8\,1_4\rangle$	1135.63	4.198	4.231	0.76	96.89	77.85	0.756
$\|1_4\rangle \to \|2_{12}\,1_4\rangle$	1631.16	3.661	3.600	1.71	96.89	88.88	0.756
$\|1_4\rangle \to \|2_{13}\,1_4\rangle$	1705.89	4.682	4.692	0.22	96.89	80.62	0.756
$\|1_5\rangle \to \|2_{12}\,1_5\rangle$	1631.16	3.773	3.432	9.94	93.75	75.77	0.770
$\|1_5\rangle \to \|2_{13}\,1_5\rangle$	1705.89	4.259	4.213	1.09	93.75	74.44	0.770
$\|1_6\rangle \to \|2_8\,1_6\rangle$	1135.63	3.901	3.891	0.25	99.01	95.38	0.974
$\|1_6\rangle \to \|2_{13}\,1_6\rangle$	1705.89	4.527	4.539	0.25	99.01	91.54	0.848
$\|1_7\rangle \to \|2_8\,1_7\rangle$	1135.63	3.875	3.901	0.65	96.86	78.30	0.514
$\|1_7\rangle \to \|2_{13}\,1_7\rangle$	1705.89	4.522	4.543	0.47	96.86	82.55	0.514
$\|1_9\rangle \to \|1_9\,2_8\rangle$	1135.63	4.354	4.351	0.06	99.02	97.31	0.800
$\|1_9\rangle \to \|2_{12}\,1_9\rangle$	1631.16	3.874	3.848	0.67	99.02	95.30	0.800
$\|1_9\rangle \to \|2_{13}\,1_9\rangle$	1705.89	4.790	4.785	0.10	99.02	89.56	0.800

Ten problematic transitions reached $\eta \geq 95\%$ with the L endpoint OPA calculation. These cases were recomputed with L and XL RR and OPA prescreening settings, as summarized in Table S6. For all ten transitions, the TD/TI RR relative difference is reduced below 1%, supporting that the larger default-prescreening deviations arise from incomplete prescreening convergence.

Table S6: Anisole problematic-transition subset. The transitions are those with default-prescreening TD/TI RR relative differences larger than 1% and L-level endpoint OPA convergence $\eta^{\mathrm{L}} \geq 95\%$. For each prescreening level, η is the smaller endpoint OPA convergence and Δ is the TD/TI RR relative difference, in percent. L uses $C_1^{\max} = 40$, $C_2^{\max} = 30$, and $N_I^{\max} = 10^9$ integrals; XL uses $C_1^{\max} = 80$, $C_2^{\max} = 60$, and $N_I^{\max} = 2 \times 10^9$ integrals.

Transition	def		L		XL		$\max_k(\mathsf{J}_{ik}^2)$
	η^{def}	Δ^{def}	η^{L}	Δ^{L}	η^{XL}	Δ^{XL}	
$\lvert 1_{13}\rangle \to \lvert 2_{13}\rangle$	95.25	2.00	98.18	0.65	98.83	0.42	0.847
$\lvert 1_{34}\rangle \to \lvert 2_{34}\rangle$	97.08	1.82	98.84	0.58	99.18	0.34	0.822
$\lvert 1_{39}\rangle \to \lvert 2_{39}\rangle$	97.78	1.56	99.24	0.50	99.46	0.37	0.987
$\lvert 1_{36}\rangle \to \lvert 1_{36}\, 1_8\rangle$	90.74	1.51	96.46	0.36	97.85	0.37	0.680
$\lvert 1_{23}\rangle \to \lvert 1_{23}\, 2_{12}\rangle$	91.00	1.43	96.89	0.49	98.22	0.44	0.948
$\lvert 1_{33}\rangle \to \lvert 1_{33}\, 2_{12}\rangle$	93.43	1.31	97.71	0.46	98.51	0.32	0.404
$\lvert 1_{16}\rangle \to \lvert 1_{16}\, 1_{12}\rangle$	92.50	1.22	96.61	0.60	97.76	0.34	0.480
$\lvert 1_{34}\rangle \to \lvert 1_{34}\, 2_{12}\rangle$	93.89	1.10	97.82	0.52	98.51	0.36	0.822
$\lvert 1_{33}\rangle \to \lvert 3_{33}\rangle$	90.41	1.06	95.71	0.33	96.73	0.29	0.404
$\lvert 1_{33}\rangle \to \lvert 1_{33}\, 2_{17}\rangle$	92.36	1.01	97.29	0.42	97.73	0.28	0.404

2.1 High mode-mixing prescreening test

A set of 100 anisole transitions was selected to stress the treatment of Duschinsky mode mixing. The selection was restricted to transitions with $I^{\mathrm{TD}} > 10^{-4}$ cm^3 mol^{-1} sr^{-1} and sorted by increasing degree of mode mixing, corresponding to decreasing values of $\max_k(\mathsf{J}_{ik}^2)$. The L calculation reduced the mean TD/TI relative error from 1.43% to 0.49%, and the maximum error from 5.83% to 2.81%.

Table S7: Anisole high mode-mixing TD/TI validation set. The default TI calculation used the standard GAUSSIAN prescreening, while the L calculation used $C_1^{\max} = 40$, $C_2^{\max} = 30$, and $N_I^{\max} = 10^9$ integrals. TD intensities are reported in units of 10^{-4} cm^3 mol^{-1} sr^{-1}; relative differences are reported in percent.

Transition	ν	I^{TD}	Δ^{def}	Δ^{L}	$\max_k(\mathsf{J}_{ik}^2)$
$\lvert 1_{30}\rangle \to \lvert 1_{30}\, 1_8\rangle$	567.81	37.400	0.16	0.00	0.360
$\lvert 1_{30}\rangle \to \lvert 1_{30}\, 1_{12}\rangle$	815.58	33.090	0.09	0.09	0.360

Continued on next page

Table S7: Anisole high mode-mixing TD/TI validation set (continued).

Transition	ν	I^{TD}	Δ^{def}	Δ^{L}	$\max_k(\mathsf{J}^2_{ik})$
$\|1_{30}\rangle \rightarrow \|1_{30}\,1_{17}\rangle$	1022.79	32.931	0.03	0.06	0.360
$\|1_{30}\rangle \rightarrow \|1_{30}\,1_{26}\rangle$	1339.20	31.288	0.05	0.01	0.360
$\|1_{30}\rangle \rightarrow \|1_{30}\,1_{18}\rangle$	1062.72	26.096	0.01	0.06	0.360
$\|1_{30}\rangle \rightarrow \|1_{30}\,1_{25}\rangle$	1300.81	17.223	0.07	0.05	0.360
$\|1_{30}\rangle \rightarrow \|1_{30}\,1_{27}\rangle$	1364.63	8.336	0.13	0.02	0.360
$\|1_{30}\rangle \rightarrow \|1_{30}\,1_{9}\rangle$	633.87	7.643	1.65	0.53	0.360
$\|1_{30}\rangle \rightarrow \|1_{33}\,1_{30}\rangle$	1675.97	6.427	1.06	0.33	0.360
$\|1_{30}\rangle \rightarrow \|1_{32}\,1_{30}\rangle$	1556.51	5.327	0.31	0.11	0.360
$\|1_{30}\rangle \rightarrow \|1_{30}\,2_{13}\rangle$	1705.89	4.726	0.62	0.10	0.360
$\|1_{30}\rangle \rightarrow \|1_{30}\,2_{8}\rangle$	1135.63	4.269	0.42	0.12	0.360
$\|1_{30}\rangle \rightarrow \|1_{30}\,1_{6}\rangle$	456.00	3.688	0.25	0.03	0.360
$\|1_{30}\rangle \rightarrow \|1_{30}\,2_{12}\rangle$	1631.16	3.533	0.89	0.47	0.360
$\|1_{30}\rangle \rightarrow \|1_{30}\,2_{5}\rangle$	862.53	3.516	2.50	1.17	0.360
$\|1_{30}\rangle \rightarrow \|1_{30}\,2_{15}\rangle$	1990.39	3.199	1.12	0.33	0.360
$\|1_{30}\rangle \rightarrow \|1_{34}\,1_{30}\rangle$	1695.04	3.159	1.61	0.58	0.360
$\|1_{30}\rangle \rightarrow \|1_{30}\,2_{17}\rangle$	2045.58	3.068	0.70	0.47	0.360
$\|1_{30}\rangle \rightarrow \|1_{30}\,2_{10}\rangle$	1435.68	2.812	0.98	0.12	0.360
$\|1_{30}\rangle \rightarrow \|1_{30}\,1_{20}\rangle$	1118.27	2.379	0.24	0.12	0.360
$\|1_{30}\rangle \rightarrow \|1_{30}\,2_{18}\rangle$	2125.44	2.351	0.16	0.21	0.360
$\|1_{30}\rangle \rightarrow \|1_{30}\,1_{21}\rangle$	1177.07	2.069	0.32	0.03	0.360
$\|1_{30}\rangle \rightarrow \|1_{30}\,1_{24}\rangle$	1215.54	1.595	0.51	0.22	0.360
$\|1_{30}\rangle \rightarrow \|1_{30}\,2_{16}\rangle$	2011.11	1.519	3.89	1.11	0.360
$\|1_{30}\rangle \rightarrow \|1_{30}\,1_{3}\rangle$	269.99	1.405	3.87	1.42	0.360
$\|1_{30}\rangle \rightarrow \|1_{30}\,2_{26}\rangle$	2678.39	1.236	0.04	0.01	0.360
$\|1_{31}\rangle \rightarrow \|1_{31}\,1_{8}\rangle$	567.81	37.507	0.20	0.01	0.383
$\|1_{31}\rangle \rightarrow \|1_{31}\,1_{12}\rangle$	815.58	33.078	0.08	0.09	0.383
$\|1_{31}\rangle \rightarrow \|1_{31}\,1_{17}\rangle$	1022.79	32.896	0.02	0.06	0.383
$\|1_{31}\rangle \rightarrow \|1_{31}\,1_{26}\rangle$	1339.20	31.303	0.08	0.01	0.383
$\|1_{31}\rangle \rightarrow \|1_{31}\,1_{18}\rangle$	1062.72	26.034	0.03	0.05	0.383
$\|1_{31}\rangle \rightarrow \|1_{31}\,1_{25}\rangle$	1300.81	17.235	0.10	0.06	0.383
$\|1_{31}\rangle \rightarrow \|1_{31}\,1_{27}\rangle$	1364.63	8.352	0.12	0.02	0.383
$\|1_{31}\rangle \rightarrow \|1_{31}\,1_{9}\rangle$	633.87	7.633	1.78	0.53	0.383
$\|1_{31}\rangle \rightarrow \|1_{33}\,1_{31}\rangle$	1675.97	6.447	0.99	0.34	0.383
$\|1_{31}\rangle \rightarrow \|1_{32}\,1_{31}\rangle$	1556.51	5.337	0.50	0.18	0.383
$\|1_{31}\rangle \rightarrow \|1_{31}\,2_{13}\rangle$	1705.89	4.723	0.75	0.11	0.383
$\|1_{31}\rangle \rightarrow \|1_{31}\,2_{8}\rangle$	1135.63	4.268	0.24	0.13	0.383
$\|1_{31}\rangle \rightarrow \|1_{31}\,1_{6}\rangle$	456.00	3.704	0.29	0.03	0.383

Continued on next page

Table S7: Anisole high mode-mixing TD/TI validation set (continued).

Transition	ν	I^{TD}	Δ^{def}	Δ^{L}	$\max_k(\mathsf{J}^2_{ik})$
$\|1_{31}\rangle \rightarrow \|1_{31}\,2_{5}\rangle$	862.53	3.524	2.86	1.12	0.383
$\|1_{31}\rangle \rightarrow \|1_{31}\,2_{12}\rangle$	1631.16	3.509	0.97	0.46	0.383
$\|1_{31}\rangle \rightarrow \|1_{31}\,2_{15}\rangle$	1990.39	3.197	1.17	0.34	0.383
$\|1_{31}\rangle \rightarrow \|1_{34}\,1_{31}\rangle$	1695.04	3.150	1.67	0.58	0.383
$\|1_{31}\rangle \rightarrow \|1_{31}\,2_{17}\rangle$	2045.58	3.057	0.71	0.46	0.383
$\|1_{31}\rangle \rightarrow \|1_{31}\,2_{10}\rangle$	1435.68	2.809	1.06	0.14	0.383
$\|1_{31}\rangle \rightarrow \|1_{31}\,1_{20}\rangle$	1118.27	2.375	0.30	0.13	0.383
$\|1_{31}\rangle \rightarrow \|1_{31}\,2_{18}\rangle$	2125.44	2.343	0.12	0.22	0.383
$\|1_{31}\rangle \rightarrow \|1_{31}\,1_{21}\rangle$	1177.07	2.073	0.14	0.02	0.383
$\|1_{31}\rangle \rightarrow \|1_{31}\,1_{24}\rangle$	1215.54	1.595	0.49	0.20	0.383
$\|1_{31}\rangle \rightarrow \|1_{31}\,2_{16}\rangle$	2011.11	1.519	4.09	1.19	0.383
$\|1_{31}\rangle \rightarrow \|1_{31}\,1_{3}\rangle$	269.99	1.417	4.04	1.39	0.383
$\|1_{31}\rangle \rightarrow \|1_{31}\,2_{26}\rangle$	2678.39	1.234	0.09	0.01	0.383
$\|1_{33}\rangle \rightarrow \|1_{33}\,1_{17}\rangle$	1022.79	33.202	0.17	0.10	0.404
$\|1_{33}\rangle \rightarrow \|1_{33}\,1_{12}\rangle$	815.58	32.401	0.46	0.20	0.404
$\|1_{33}\rangle \rightarrow \|1_{33}\,1_{26}\rangle$	1339.20	30.492	0.06	0.06	0.404
$\|1_{33}\rangle \rightarrow \|1_{33}\,1_{8}\rangle$	567.81	30.390	0.24	0.14	0.404
$\|1_{33}\rangle \rightarrow \|1_{33}\,1_{18}\rangle$	1062.72	27.349	0.51	0.22	0.404
$\|1_{33}\rangle \rightarrow \|1_{33}\,1_{25}\rangle$	1300.81	16.721	0.12	0.07	0.404
$\|1_{33}\rangle \rightarrow \|2_{33}\rangle$	1675.97	12.795	0.90	0.30	0.404
$\|1_{33}\rangle \rightarrow \|1_{33}\,1_{27}\rangle$	1364.63	8.120	0.00	0.04	0.404
$\|1_{33}\rangle \rightarrow \|1_{33}\,1_{9}\rangle$	633.87	7.951	1.65	0.50	0.404
$\|1_{34}\rangle \rightarrow \|1_{34}\,1_{33}\rangle$	1675.97	6.489	0.96	0.37	0.404
$\|1_{29}\rangle \rightarrow \|1_{33}\,1_{29}\rangle$	1675.97	6.469	1.60	0.39	0.404
$\|0\rangle \rightarrow \|1_{33}\rangle$	1675.97	6.467	0.11	0.05	0.404
$\|1_{22}\rangle \rightarrow \|1_{33}\,1_{22}\rangle$	1675.97	6.465	2.34	0.49	0.404
$\|1_{28}\rangle \rightarrow \|1_{33}\,1_{28}\rangle$	1675.97	6.455	1.49	0.53	0.404
$\|1_{23}\rangle \rightarrow \|1_{33}\,1_{23}\rangle$	1675.97	6.448	1.58	0.54	0.404
$\|1_{37}\rangle \rightarrow \|1_{37}\,1_{33}\rangle$	1675.97	6.442	1.47	0.54	0.404
$\|1_{40}\rangle \rightarrow \|1_{40}\,1_{33}\rangle$	1675.97	6.441	1.31	0.47	0.404
$\|1_{35}\rangle \rightarrow \|1_{35}\,1_{33}\rangle$	1675.97	6.433	2.81	0.79	0.404
$\|1_{36}\rangle \rightarrow \|1_{36}\,1_{33}\rangle$	1675.97	6.431	2.29	0.69	0.404
$\|1_{38}\rangle \rightarrow \|1_{38}\,1_{33}\rangle$	1675.97	6.426	2.76	0.78	0.404
$\|1_{19}\rangle \rightarrow \|1_{33}\,1_{19}\rangle$	1675.97	6.422	0.56	0.12	0.404
$\|1_{39}\rangle \rightarrow \|1_{39}\,1_{33}\rangle$	1675.97	6.421	1.22	0.52	0.404
$\|1_{42}\rangle \rightarrow \|1_{42}\,1_{33}\rangle$	1675.97	6.419	2.38	0.73	0.404
$\|1_{2}\rangle \rightarrow \|1_{33}\,1_{2}\rangle$	1675.97	6.415	4.61	2.68	0.404

Continued on next page

Table S7: Anisole high mode-mixing TD/TI validation set (continued).

Transition	ν	I^{TD}	Δ^{def}	Δ^{L}	$\max_k(\mathsf{J}^2_{ik})$
$\vert 1_{20}\rangle \rightarrow \vert 1_{33}\, 1_{20}\rangle$	1675.97	6.411	0.98	0.33	0.404
$\vert 1_{1}\rangle \rightarrow \vert 1_{33}\, 1_{1}\rangle$	1675.97	6.410	4.54	1.24	0.404
$\vert 1_{24}\rangle \rightarrow \vert 1_{33}\, 1_{24}\rangle$	1675.97	6.405	0.82	0.34	0.404
$\vert 1_{3}\rangle \rightarrow \vert 1_{33}\, 1_{3}\rangle$	1675.97	6.398	0.81	0.14	0.404
$\vert 1_{41}\rangle \rightarrow \vert 1_{41}\, 1_{33}\rangle$	1675.97	6.397	1.36	0.75	0.404
$\vert 1_{6}\rangle \rightarrow \vert 1_{33}\, 1_{6}\rangle$	1675.97	6.387	0.74	0.23	0.404
$\vert 1_{4}\rangle \rightarrow \vert 1_{33}\, 1_{4}\rangle$	1675.97	6.351	3.91	1.10	0.404
$\vert 1_{9}\rangle \rightarrow \vert 1_{33}\, 1_{9}\rangle$	1675.97	6.339	1.59	0.48	0.404
$\vert 1_{7}\rangle \rightarrow \vert 1_{33}\, 1_{7}\rangle$	1675.97	6.314	3.94	2.23	0.404
$\vert 1_{11}\rangle \rightarrow \vert 1_{33}\, 1_{11}\rangle$	1675.97	6.311	5.15	1.53	0.404
$\vert 1_{32}\rangle \rightarrow \vert 1_{33}\, 1_{32}\rangle$	1675.97	6.291	0.86	0.28	0.404
$\vert 1_{14}\rangle \rightarrow \vert 1_{33}\, 1_{14}\rangle$	1675.97	6.255	5.16	1.40	0.404
$\vert 1_{16}\rangle \rightarrow \vert 1_{33}\, 1_{16}\rangle$	1675.97	6.177	5.23	1.49	0.404
$\vert 1_{21}\rangle \rightarrow \vert 1_{33}\, 1_{21}\rangle$	1675.97	6.164	1.02	0.35	0.404
$\vert 1_{5}\rangle \rightarrow \vert 1_{33}\, 1_{5}\rangle$	1675.97	6.163	5.83	2.22	0.404
$\vert 1_{10}\rangle \rightarrow \vert 1_{33}\, 1_{10}\rangle$	1675.97	6.087	5.00	2.81	0.404
$\vert 1_{15}\rangle \rightarrow \vert 1_{33}\, 1_{15}\rangle$	1675.97	6.059	5.77	1.16	0.404
$\vert 1_{13}\rangle \rightarrow \vert 1_{33}\, 1_{13}\rangle$	1675.97	6.016	5.47	1.28	0.404
$\vert 1_{27}\rangle \rightarrow \vert 1_{33}\, 1_{27}\rangle$	1675.97	5.884	0.93	0.34	0.404
$\vert 1_{8}\rangle \rightarrow \vert 1_{33}\, 1_{8}\rangle$	1675.97	5.738	1.67	0.38	0.404
$\vert 1_{25}\rangle \rightarrow \vert 1_{33}\, 1_{25}\rangle$	1675.97	5.670	1.81	0.56	0.404
$\vert 1_{26}\rangle \rightarrow \vert 1_{33}\, 1_{26}\rangle$	1675.97	5.118	2.51	0.75	0.404
$\vert 1_{33}\rangle \rightarrow \vert 1_{33}\, 1_{32}\rangle$	1556.51	5.063	0.18	0.06	0.404
$\vert 1_{18}\rangle \rightarrow \vert 1_{33}\, 1_{18}\rangle$	1675.97	5.029	1.99	0.57	0.404

3 Additional data for fluorobenzene

3.1 Vibrational modes of fluorobenzene

Table S8: Ground-state (S_0) harmonic vibrational wavenumbers (ω, in cm^{-1}) and irreducible representation of the normal modes of fluorobenzene at the CAM-B3LYP/def2-TZVP level.

Mode	irrep	ω	Mode	irrep	ω
1	$B1$	242.8	16	$B2$	1103.3
2	$B2$	412.3	17	$B2$	1184.9
3	$A2$	432.1	18	$A1$	1186.3
4	$B1$	520.2	19	$A1$	1275.0
5	$A1$	532.7	20	$B2$	1326.2
6	$B2$	635.3	21	$B2$	1354.7
7	$B1$	709.0	22	$B2$	1512.7
8	$B1$	784.5	23	$A1$	1552.4
9	$A1$	838.9	24	$A1$	1677.7
10	$A2$	852.5	25	$B2$	1683.6
11	$B1$	934.3	26	$A1$	3196.4
12	$A2$	989.9	27	$B2$	3204.9
13	$B1$	995.3	28	$A1$	3217.2
14	$A1$	1037.3	29	$B2$	3225.7
15	$A1$	1055.3	30	$A1$	3228.0

3.2 Duschinsky transformation for the $S_0 \rightarrow S_1$ transition

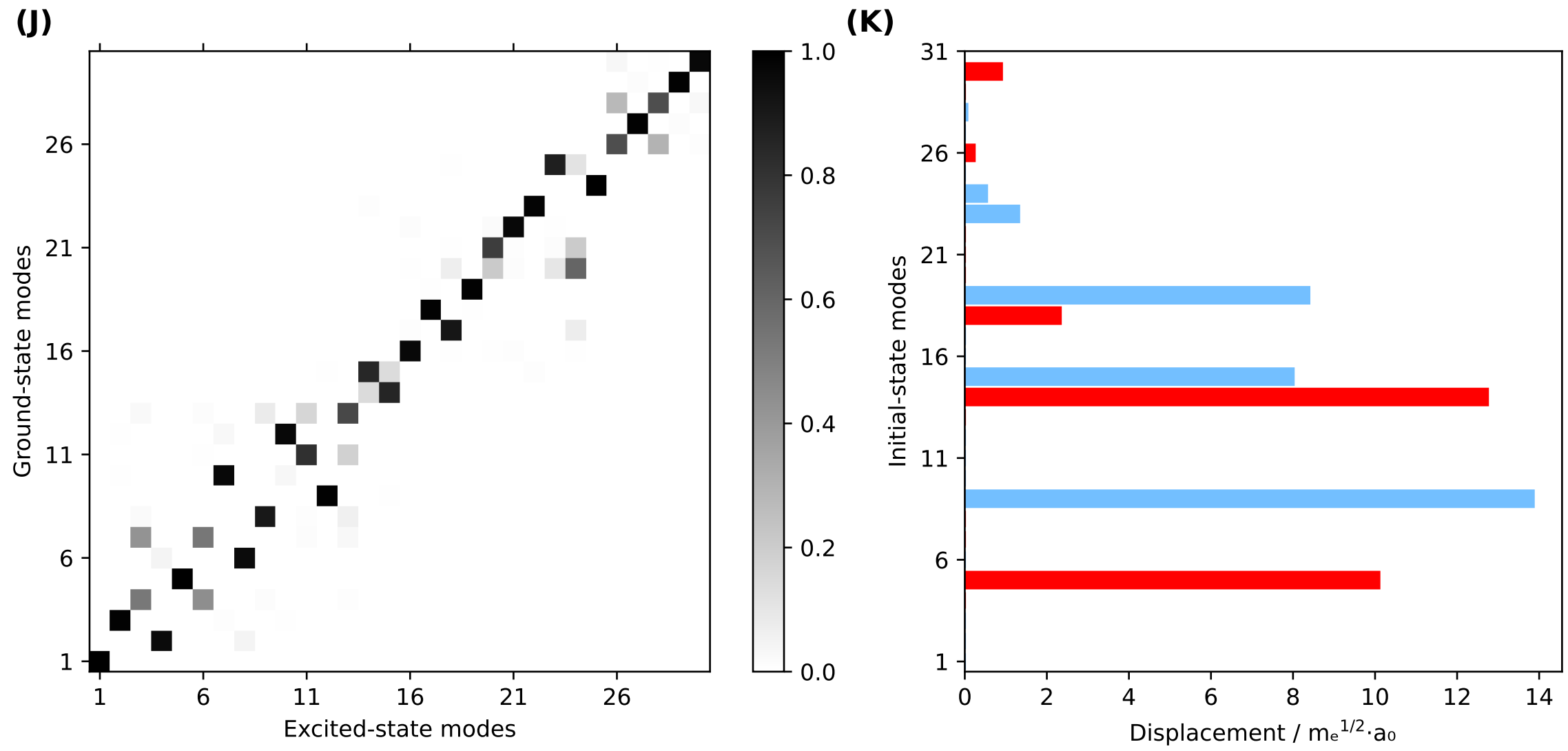

Figure S6: Duschinsky matrix (**J**, left panel) and shift vector (**K**, right panel) associated to the $S_0 \rightarrow S_1$ transition of fluorobenzene within the adiabatic Hessian model. Each matrix element has been squared and a shade of gray assigned based on the value (1: black, 0: white). The shift vector is reported in atomic units. Red bars indicate negative values, blue for positive ones.

3.3 Influence of temperature effects at different resonance conditions

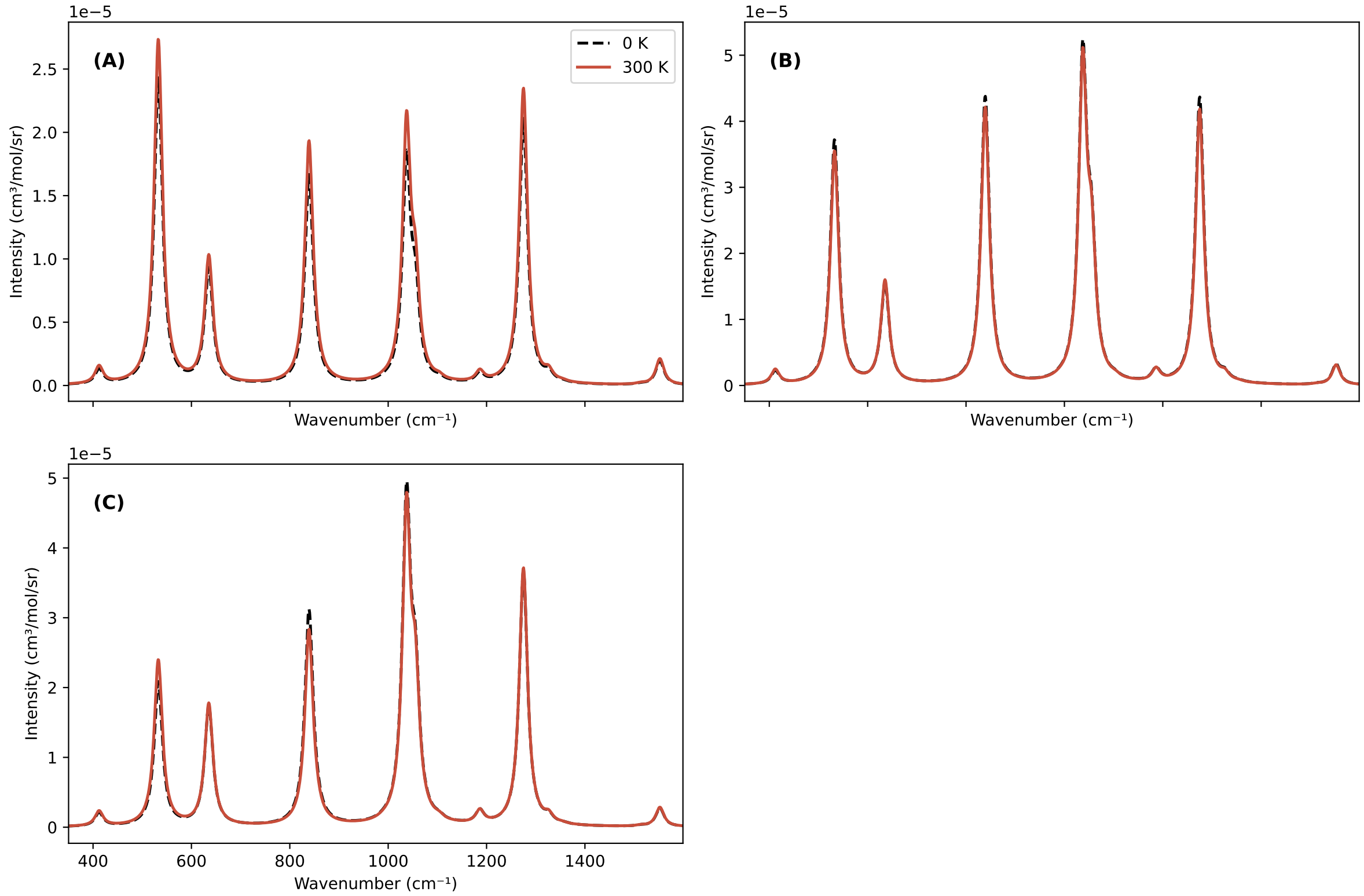

Figure S7: Comparison of the RR spectra of fluorobenzene at 0 K and 300 K spectra with damping factor $\gamma = 500\,\mathrm{cm}^{-1}$, using a 1% population cutoff for the thermal ensemble. (A) Incident frequency shifted by $-500\,\mathrm{cm}^{-1}$ relative to the 0-0 resonance. (B) Incident frequency equal to the 0-0 resonance.(C) Incident frequency shifted by $+500\,\mathrm{cm}^{-1}$ relative to the 0-0 resonance.

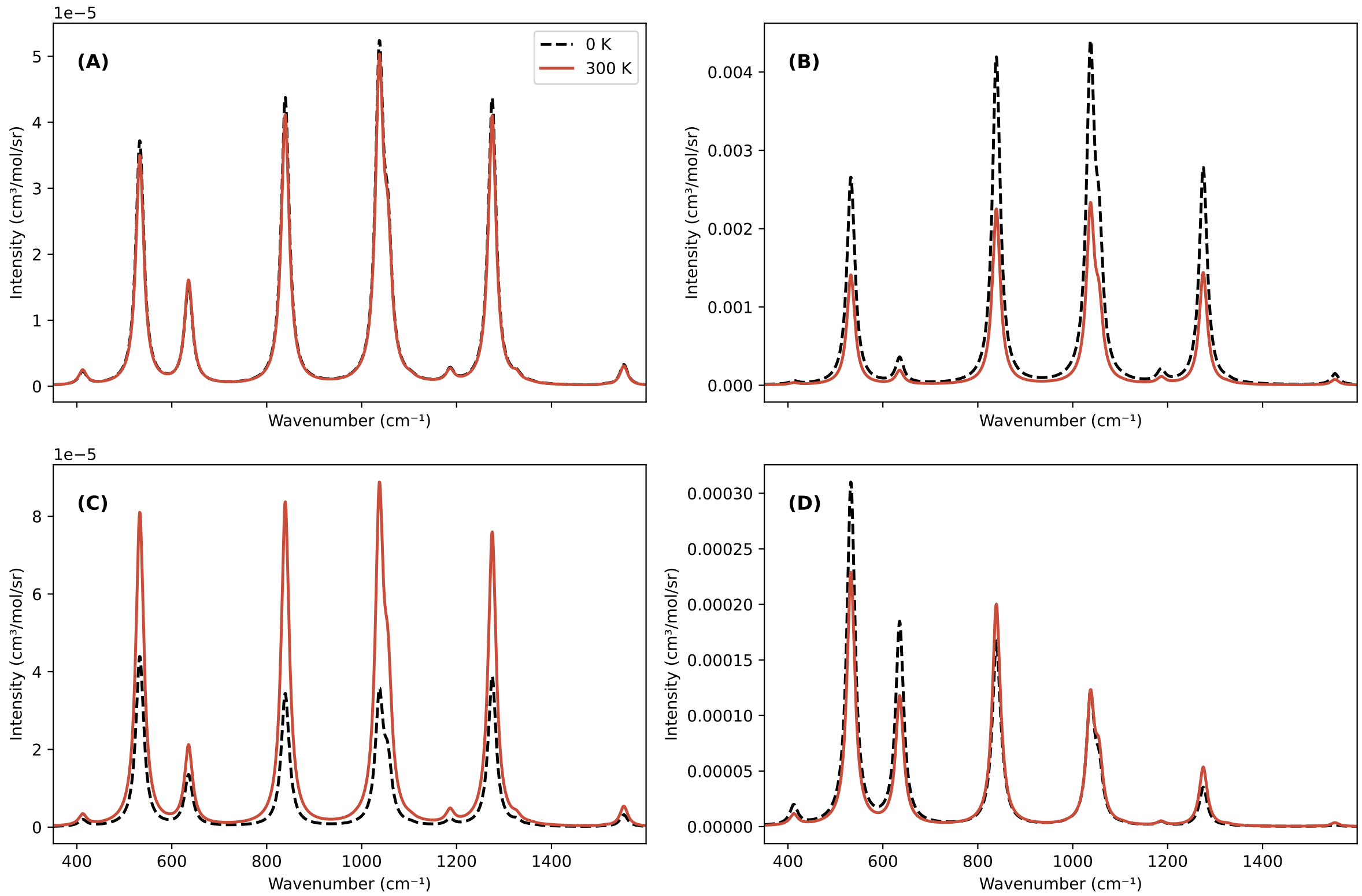

Figure S8: Comparison of the RR spectra of fluorobenzene at 0 K and 300 K using all thermally populated initial states up to order 2 available in the transition list, without applying the 1% population cutoff used in the main text. This expanded thermal ensemble accounts for 92.00% of the canonical partition function, compared with 81.76% for the 1% cutoff. The same qualitative dependence on resonance conditions is retained. (A) $\gamma = 500\,\mathrm{cm}^{-1}$, incident frequency at the 0-0 resonance. (B) $\gamma = 50\,\mathrm{cm}^{-1}$, incident frequency at the 0-0 resonance. (C) $\gamma = 50\,\mathrm{cm}^{-1}$, incident frequency shifted by $-500\,\mathrm{cm}^{-1}$. (D) $\gamma = 50\,\mathrm{cm}^{-1}$, incident frequency shifted by $+500\,\mathrm{cm}^{-1}$.

4 Additional data for (*S*)-(+)-naproxen

4.1 Electronic one-photon absorption spectrum

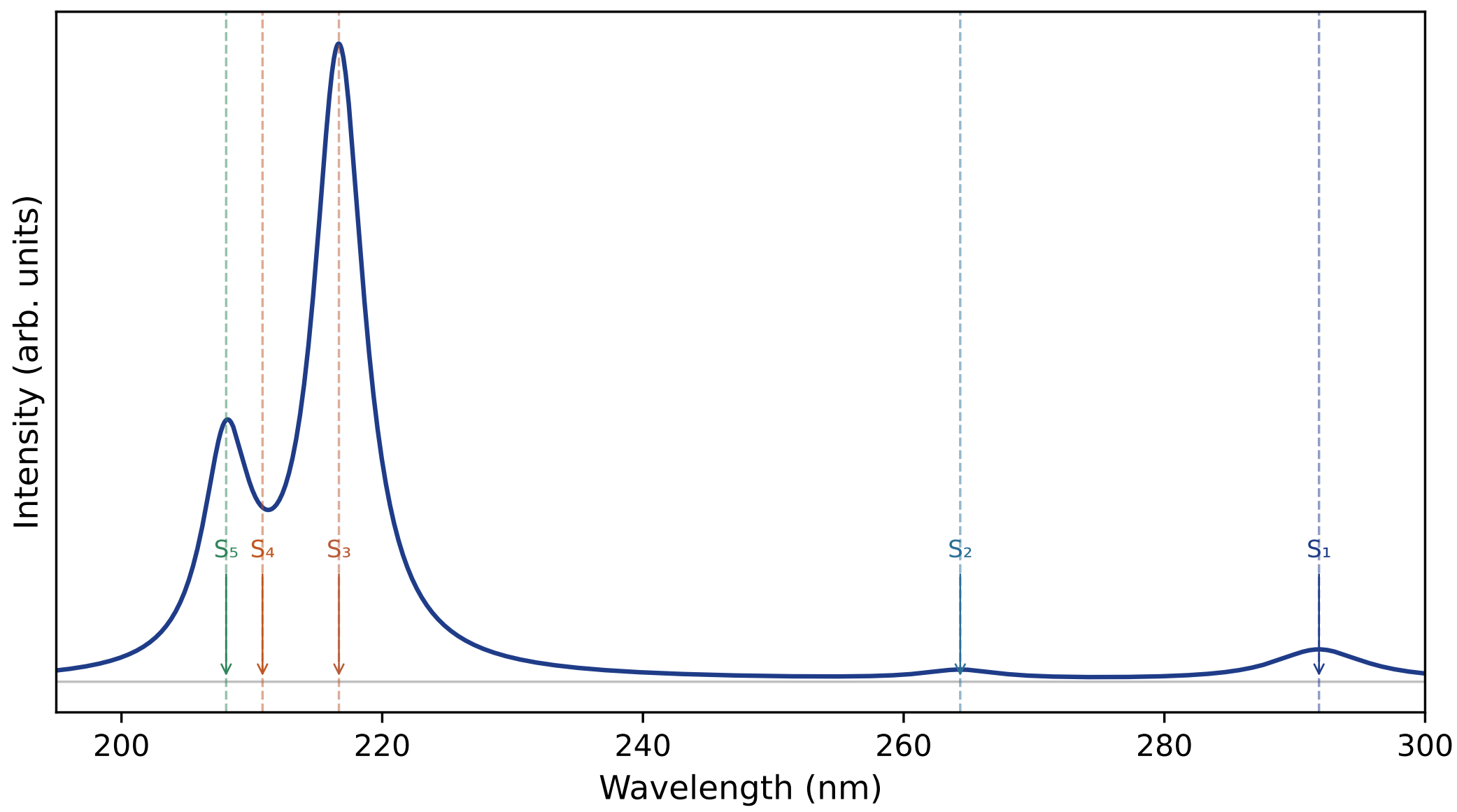

Figure S9: Naproxen electronic absorption spectrum computed at the CAM-B3LYP/def2-TZVP level. The stick heights are proportional to the oscillator strengths, and a Lorentzian broadening of 500 cm^{-1} HWHM has been applied.

4.2 Duschinsky transformations for first two excited states

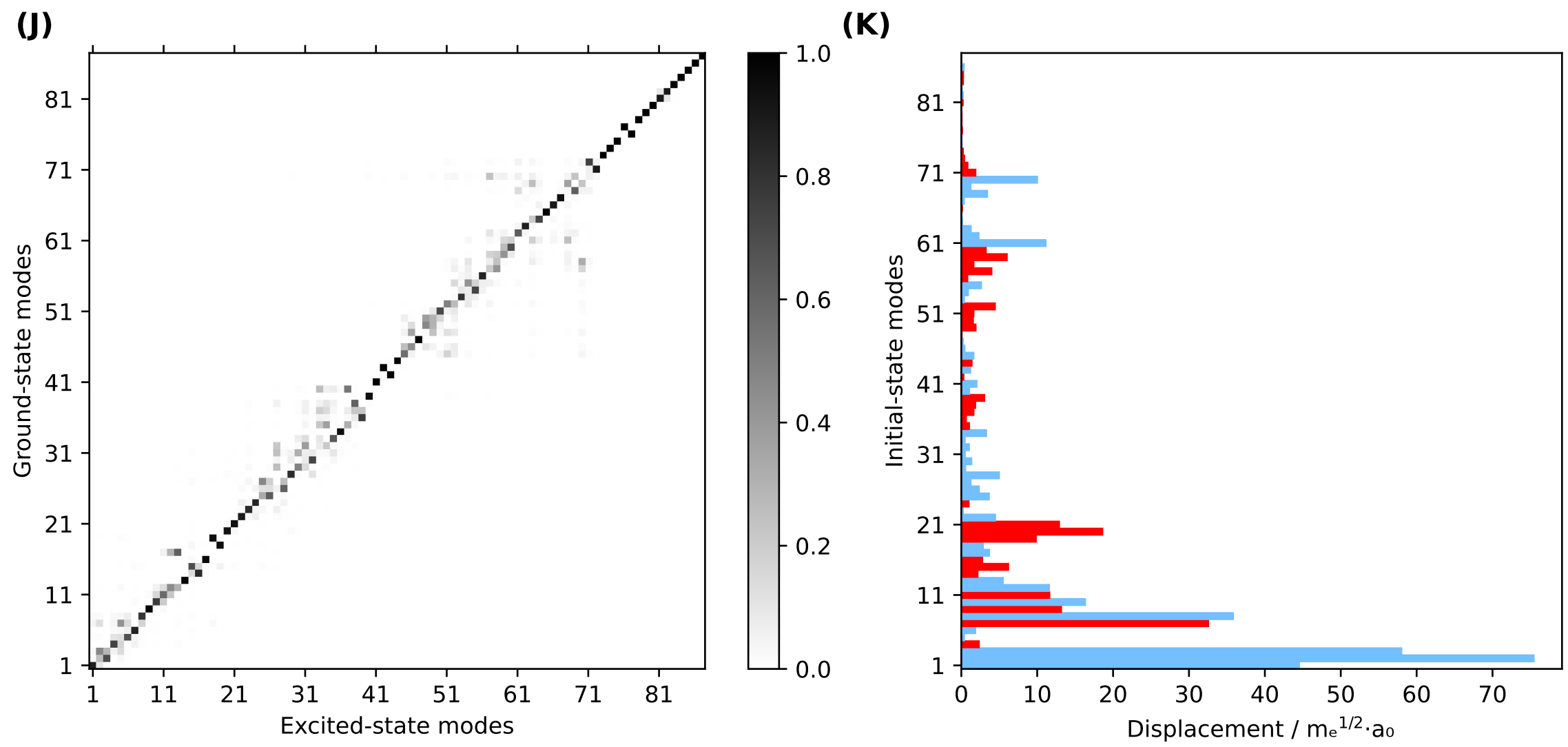

Figure S10: Duschinsky matrix (**J**, left panel) and shift vector (**K**, right panel) associated to the $S_0 \rightarrow S_1$ transition of (S)-(+)-naproxen within the vertical Hessian model. Each matrix element has been squared and a shade of gray assigned based on the value (1: black, 0: white). The shift vector is reported in atomic units. Red bars indicate negative values, blue for positive ones.

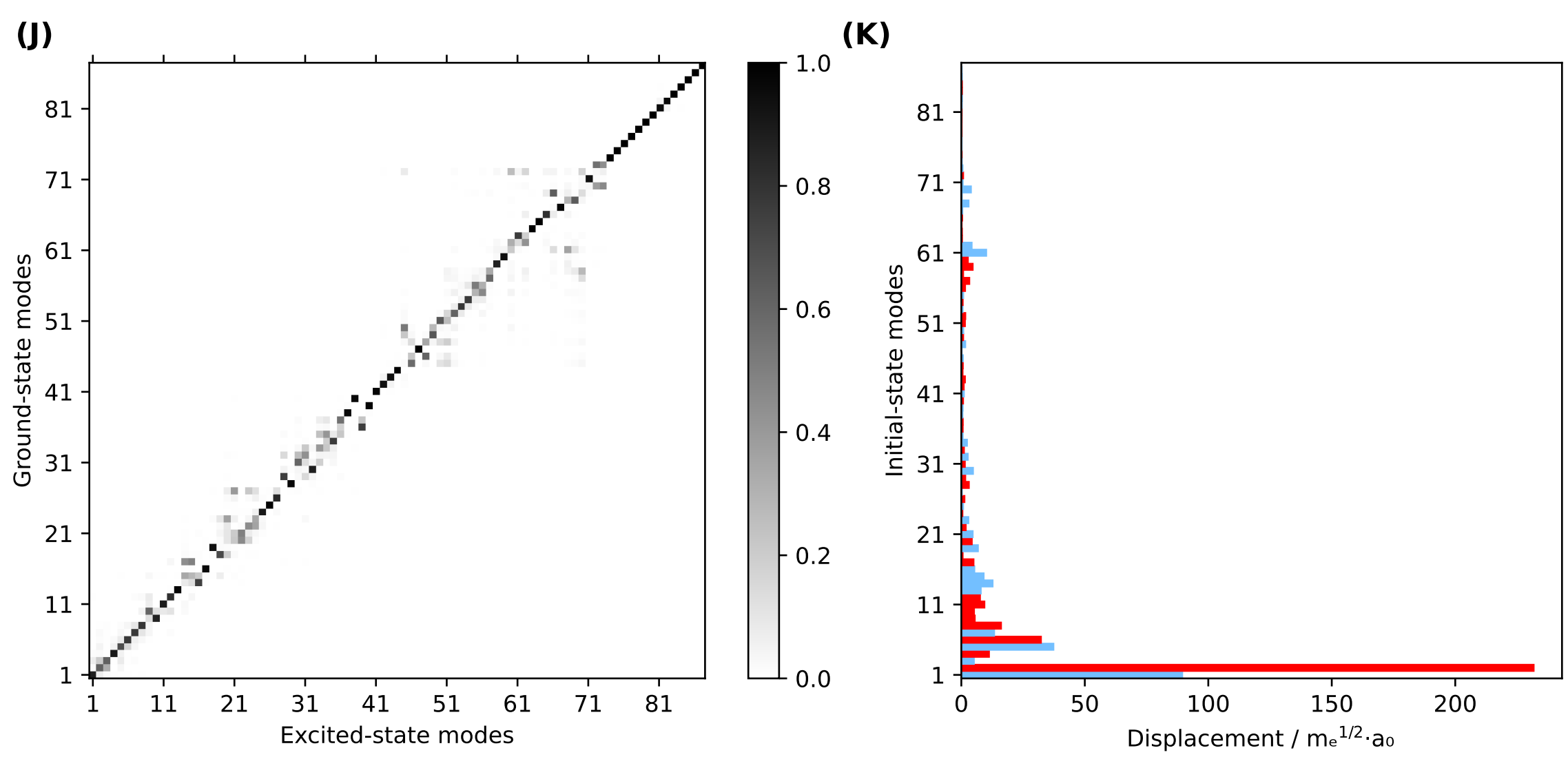

Figure S11: Duschinsky matrix (**J**, left panel) and shift vector (**K**, right panel) associated to the $S_0 \rightarrow S_2$ transition of (S)-(+)-naproxen within the vertical Hessian model. Each matrix element has been squared and a shade of gray assigned based on the value (1: black, 0: white). The shift vector is reported in atomic units. Red bars indicate negative values, blue for positive ones.

4.3 Resonance ROA spectra

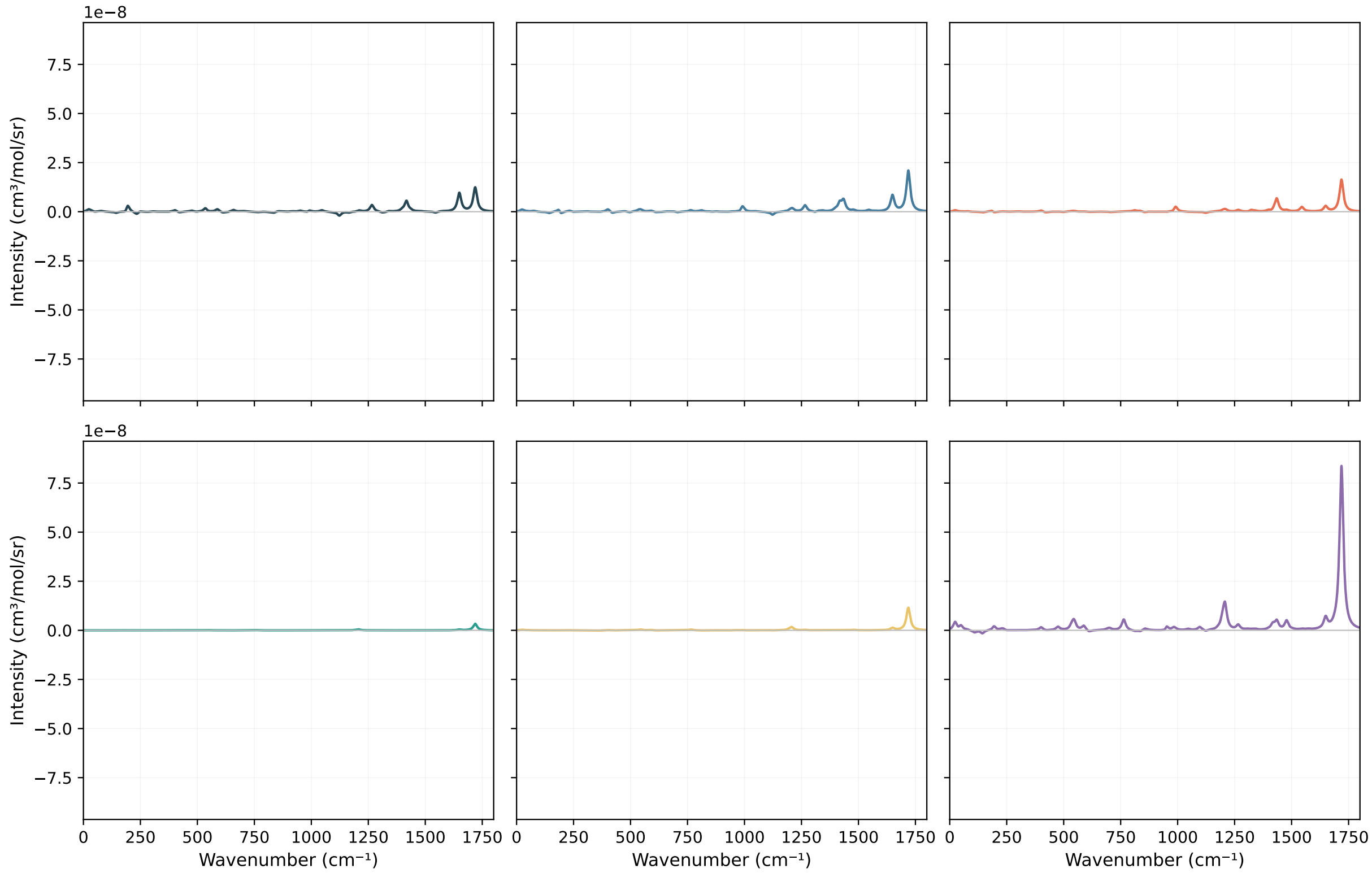

Figure S12: Single electronic state RROA spectra of (S)-(+)-naproxen with intermediate states S_1 (top row) and S_2 (bottom row). Three incident frequencies were considered, in resonance with S_1 (at 30865.10 cm^{-1}, left column) at the midpoint between the two electronic states (33296.87 cm^{-1}, middle column), and in resonance with S_2 (35728.73 cm^{-1}, right column). Lorentzian functions with half-widths at half-maximum of 10 cm^{-1} were used to simulate the broadening.

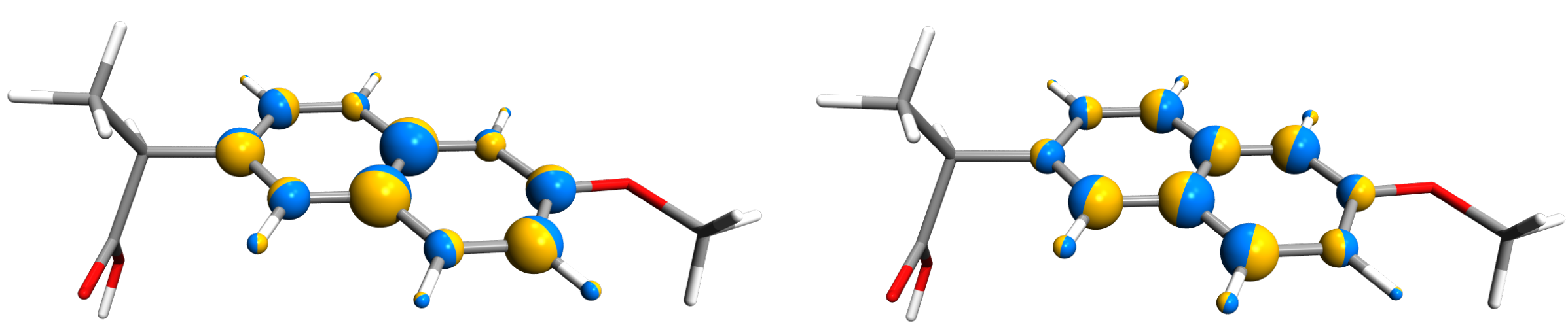

Figure S13: Graphical representation of modes 70 (left) and 72 (right) of (S)-(+)-naproxen. Nuclear displacements are perpendicular to the separation between each half-sphere and their magnitude proportional to the diameter of the spheres.

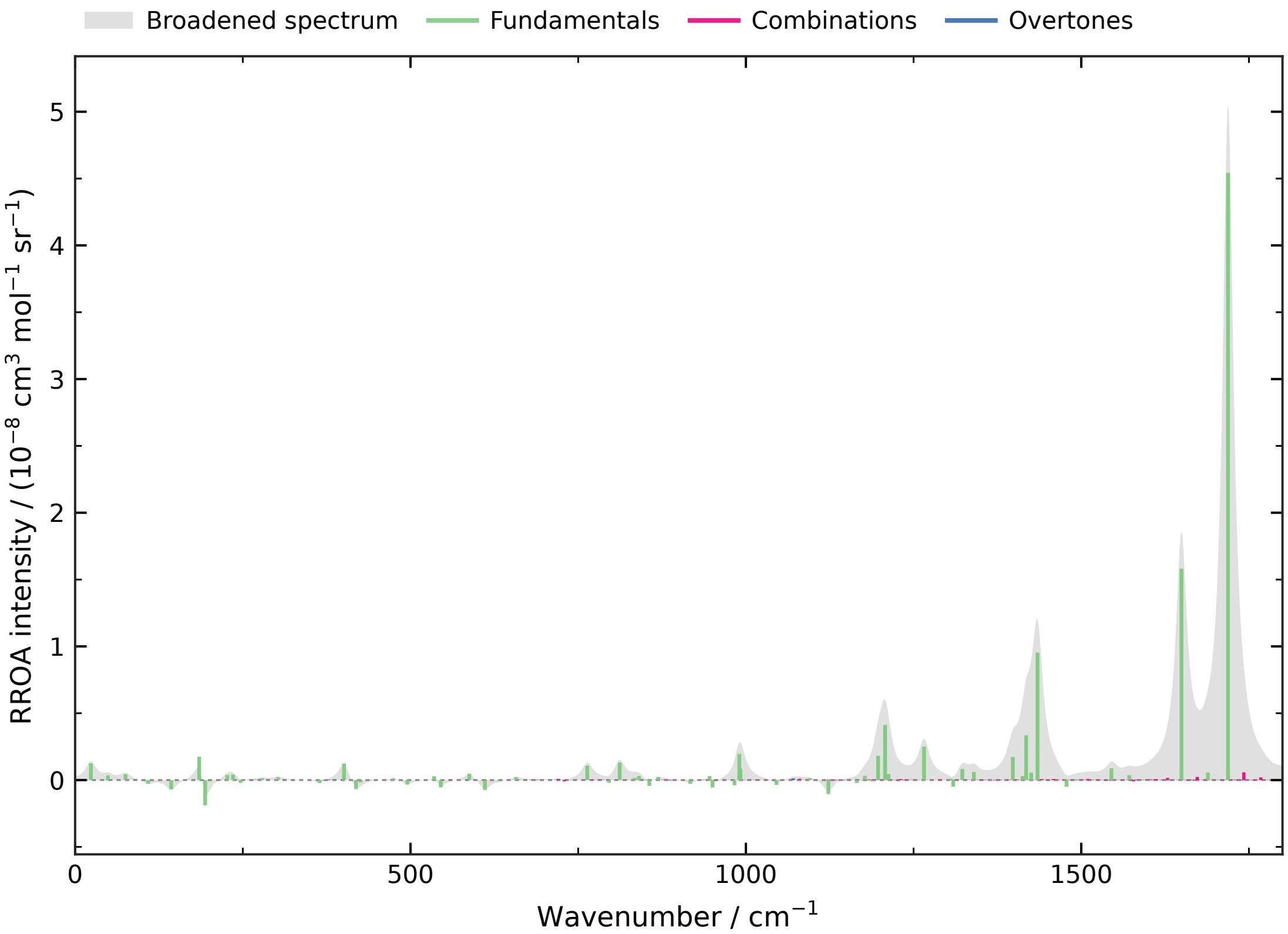

Figure S14: Fundamental region (0–1800 cm^{-1}) of the RROA spectrum of (S)-(+)-naproxen including two intermediate states S_1 and S_2 with the incident energy set at mid-point with energy $\omega_I = 33\,296.87\,\mathrm{cm}^{-1}$ (≈323 nm). Contributions to the RROA spectrum are overlaid, showing the relative intensities of fundamentals, '1+1' combinations, and first overtones against the total broadened spectrum.
